# In situ structures of plant photosystem supercomplexes

**DOI:** 10.1038/s41586-026-10847-3

**Published:** 2026-07-29

**Authors:** Jiao Li, Eduard Elias, Kai Zhang, Roberta Croce, Jiapeng Zhu

**Affiliations:** 1 Department of Cardiology, The First Affiliated Hospital of USTC, MOE Key Laboratory for Cellular Dynamics, Center for Advanced Interdisciplinary Science and Biomedicine of IHM, Division of Life Sciences and Medicine, Hefei National Research Center for Interdisciplinary Sciences at the Microscale, University of Science and Technology of China, Hefei, China; 2 School of Medicine, Nanjing University of Chinese Medicine, Nanjing, China; 3 Department of Physics and Astronomy, Faculty of Science, Vrije Universiteit Amsterdam, Amsterdam, The Netherlands; 4 Institute for Lasers, Life and Biophotonics, Faculty of Science, Vrije Universiteit Amsterdam, Amsterdam, The Netherlands; 5 Department of Hepatobiliary Surgery, The First Affiliated Hospital of Anhui Medical University, Hefei, China; 6 MOE Innovation Center for Basic Research in Tumor Immunotherapy, Hefei, China; 7 Anhui Province Key Laboratory of Tumor Immune Microenvironment and Immunotherapy, Hefei, China; 8 Anhui Provincial Innovation Institute for Pharmaceutical Basic Research, Hefei, Anhui China

**Keywords:** Photosystem II, Photosystem I, Antenna complex, Cryoelectron microscopy, Protein analysis

## Abstract

Photosynthesis sustains life on Earth by converting light to chemical energy through the coordinated action of photosystem I (PSI) and photosystem II (PSII) within thylakoid membranes^1–4^. Although structures of isolated photosystems are available, their native organization in chloroplasts remains unknown. Here, using in situ cryo-electron microscopy, we directly imaged *Oryza sativa* (rice) chloroplasts and determined structures of photosystem supercomplexes in their native membrane environment. We resolved a C_2_S_2_M_2_L_4_-type PSII–light harvesting complex II (LHCII) supercomplex, including four LHCII antenna trimers that were not retained in purified preparations. Excitation energy transfer calculations based on this architecture closely reproduce in vivo measurements, indicating its physiological relevance. We also resolved asymmetric PSII–LHCII dimers, including side-by-side, trans-lumenal and trans-stromal architectures, and higher-order assemblies of trimers and tetramers. On the basis of these observations, we propose that PSII forms a trans-lumenal and trans-stromal ‘skeleton’ that shapes thylakoid morphology and supports grana stacking. In addition, we obtained high-resolution structures of PSI–LHCI–LHCII and PSI–LHCI supercomplexes. Together, these structures reveal extensive networks of lipids, pigments and cofactors, providing the first molecular framework for understanding how the native architecture of plant photosystem supports the exceptional photon-to-electron efficiency of photosynthesis.

## Abstract (web summary)

The cryo‑EM structure of the *Oryza sativa* photosystem II–light harvesting complex II supercomplex in its native membrane reveals the intact plant photosystem architecture, retaining physiologically relevant proteins and lipids that are otherwise lost during purification.

## Main

Chloroplasts, the photosynthetic organelles of plants, convert light to chemical energy through specialized pigment–protein complexes embedded in the thylakoid membrane. PSII and PSI operate in series to drive electron transport from water to NADP^+^ alongside the cytochrome *b*_6_*f* complex. These megadalton assemblies contain conserved core modules housing reaction centres for charge separation, and their light-harvesting antennae, which are diversified across taxa and encoded in plants and green algae by the light-harvesting complex (LHC) multigene family^5^, increase photon capture and deliver excitation energy to reaction centres, sustaining Earth’s primary productivity.

Cryo-electron microscopy (cryo-EM) and serial femtosecond crystallography structural studies using purified complexes have resolved the supramolecular organization of PSII and PSI from various plants^2,3,6,7^, algae^8–13^ and photosynthetic bacteria^14–20^. Although these structural analyses have revealed the subunit compositions and pigment arrangements of the supercomplexes, substantial discrepancies persist between these in vitro structures and physiological evidence. Biochemical and functional data demonstrate that native PSI and PSII antenna systems are substantially larger than their purified counterparts, as indicated by a higher LHC-to-core ratio and pigment stoichiometry per reaction centre in vivo (for example, in ref. ^21^). Time-resolved fluorescence measurements further corroborate this conclusion, showing longer excited state lifetimes in vivo compared with purified samples (for example, refs. ^22,23^), consistent with an expanded antenna system in which increased chlorophyll content prolongs excitation energy transfer (EET) pathways^1^.

These differences are likely to originate from inherent limitations of conventional in vitro approaches: (1) loss of native environmental context; (2) potential failure to preserve physiologically relevant functional assemblies, particularly due to disruption of weak or transient interactions during purification procedures; and (3) consequent inability to isolate native complex populations. Moreover, detergent solubilization can induce conformational changes in flexible regions of membrane proteins, which may adopt non-native conformations. These limitations are especially critical in highly organized membrane regions such as grana thylakoids, where the native 3D architecture may influence photosystem arrangement, stability and regulatory interactions^24–27^, posing fundamental challenges in elucidating physiological assemblies and molecular mechanisms.

**Fig. 1:**
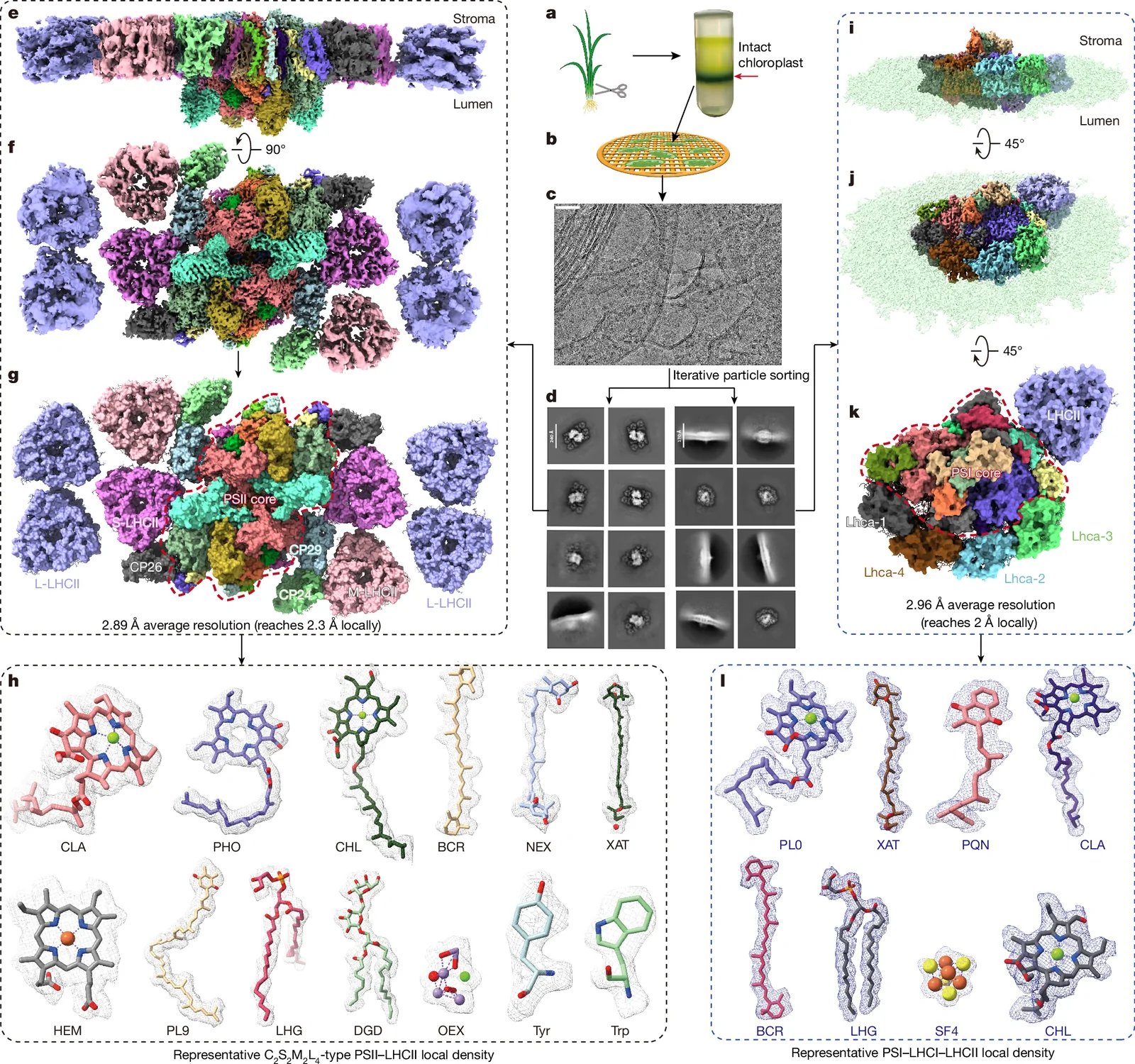
In situ single-particle cryo-EM analysis of photosynthetic supercomplexes in thylakoid membranes from *O. sativa* chloroplasts. **a**,**b**, Cryo-EM grid preparation of chloroplasts isolated from *O. sativa* (**a**) and directly frozen onto cryo-EM grids (**b**). **c**, Representative cryo-EM micrograph of chloroplasts for single-particle analysis. Similar micrograph quality was observed across the entire dataset (*n* = 22,785 micrographs). Scale bar, 50 nm. **d**, Representative 2D class averages showing different types of photosynthetic supercomplex. **e**,**f**, Side (**e**) and lumenal (**f**) views of a representative high-resolution map of the C_2_S_2_M_2_L_4_-type PSII–LHCII supercomplex (Electron Microscopy Data Bank (EMDB) EMD-68060). **g**, Surface representation of the C_2_S_2_M_2_L_4_ complex (Protein Data Bank (PDB) 21WV), comprising the core (outlined by the red dashed box) and peripheral antenna proteins: CP29, CP24, CP26, S-LHCII, M-LHCII and L-LHCII. The L-LHCII regions were fitted with the LHCII models from the PSI–LHCI–LHCII complex according to their distinct triangular densities. **h**, High-resolution features of the C_2_S_2_M_2_L_4_ complex shown by representative density of amino acid residues and endogenous ligands. CLA, chlorophyll *a*; PHO, pheophytin *a*; CHL, chlorophyll *b*; BCR, β-carotene; NEX, neoxanthin; XAT, violaxanthin; HEM, heme; PL9, plastoquinone-9; LHG, 1,2-dipalmitoyl-phosphatidyl-glycerole; DGD, digalactosyl diacylglycerol. **i**,**j**, Side (**i**) and stromal (**j**) views of a representative high-resolution map of the PSI–LHCI–LHCII supercomplex (EMDB EMD-66932) in the native thylakoid membrane. **k**, Atomic model of PSI–LHCI–LHCII (PDB 9XJ9). **l**, High-resolution features of PSI–LHCI–LHCII shown by representative density of amino acid residues and endogenous ligands. PL0, chlorophyll *a* isomer; PQN, phylloquinone; SF4, iron/sulfur cluster.

**Extended Data Fig. 1:**
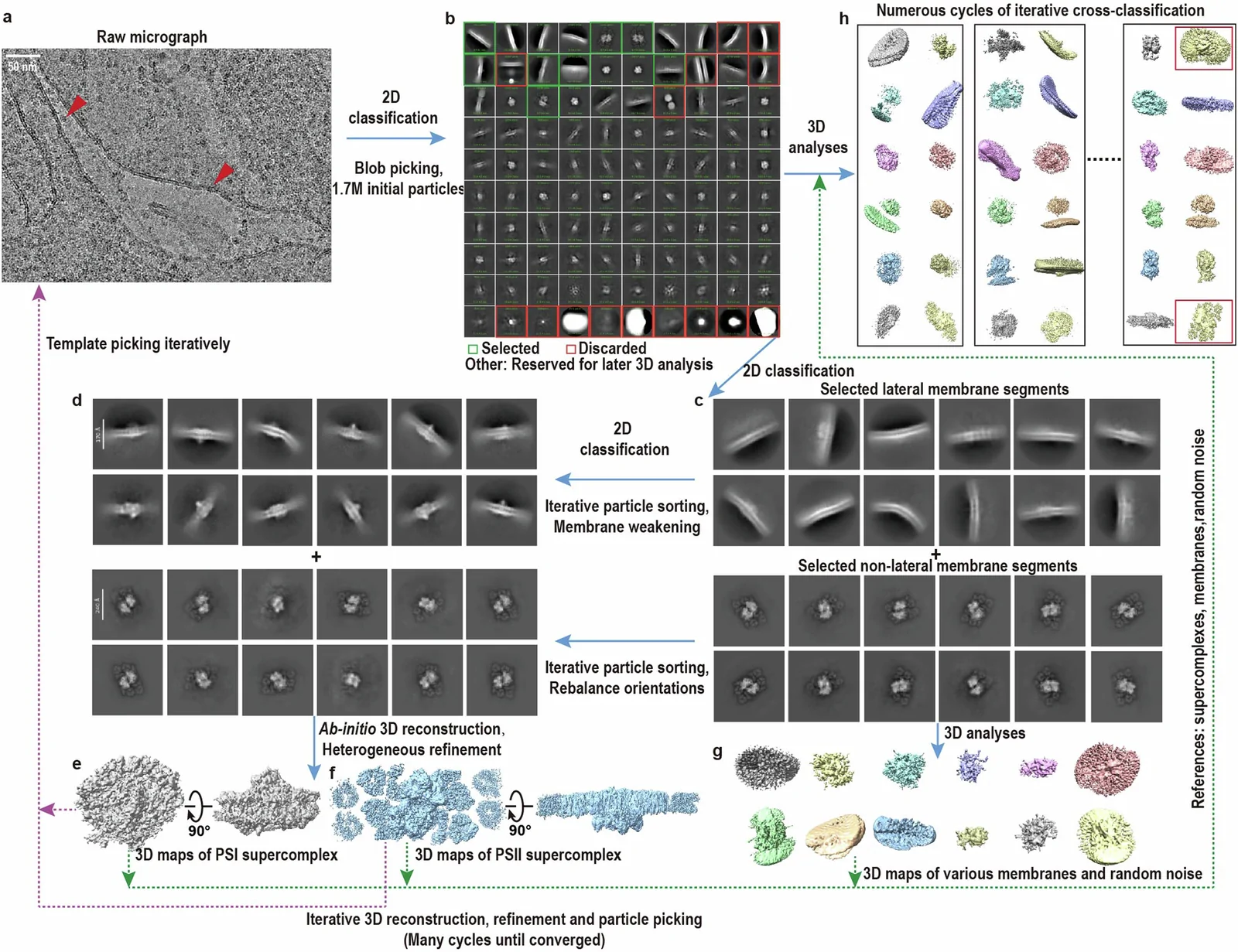
Schematic workflow for initial single-particle cryo-EM analysis of photosynthetic complexes. **a**, High-magnification micrograph of a representative single-particle chloroplast sample. **b**, Initial reference-free 2D classification results. **c**, Representative 2D subclass averages displaying distinct lateral and non-lateral orientations of thylakoid membrane-embedded particles. **d**, Representative 2D class averages of thylakoid membrane side-views following iterative particle sorting (top), alongside orientation-rebalanced representative non-side-view 2D class averages (bottom). **e**-**g**, Respective 3D reconstructions generated using particles selected from the classification steps shown in (**c**) and (**d**), respectively. **h**, Iterative cross-3D classification outcomes. Through multiple cycles of reference map refinement and particle alignment parameter optimization, reliable 3D reference models were obtained for subsequent multi-level refinement and focused classification. All downstream processing was performed separately on merged particle subsets corresponding to PSI and PSII supercomplexes.

**Extended Data Fig. 2:**
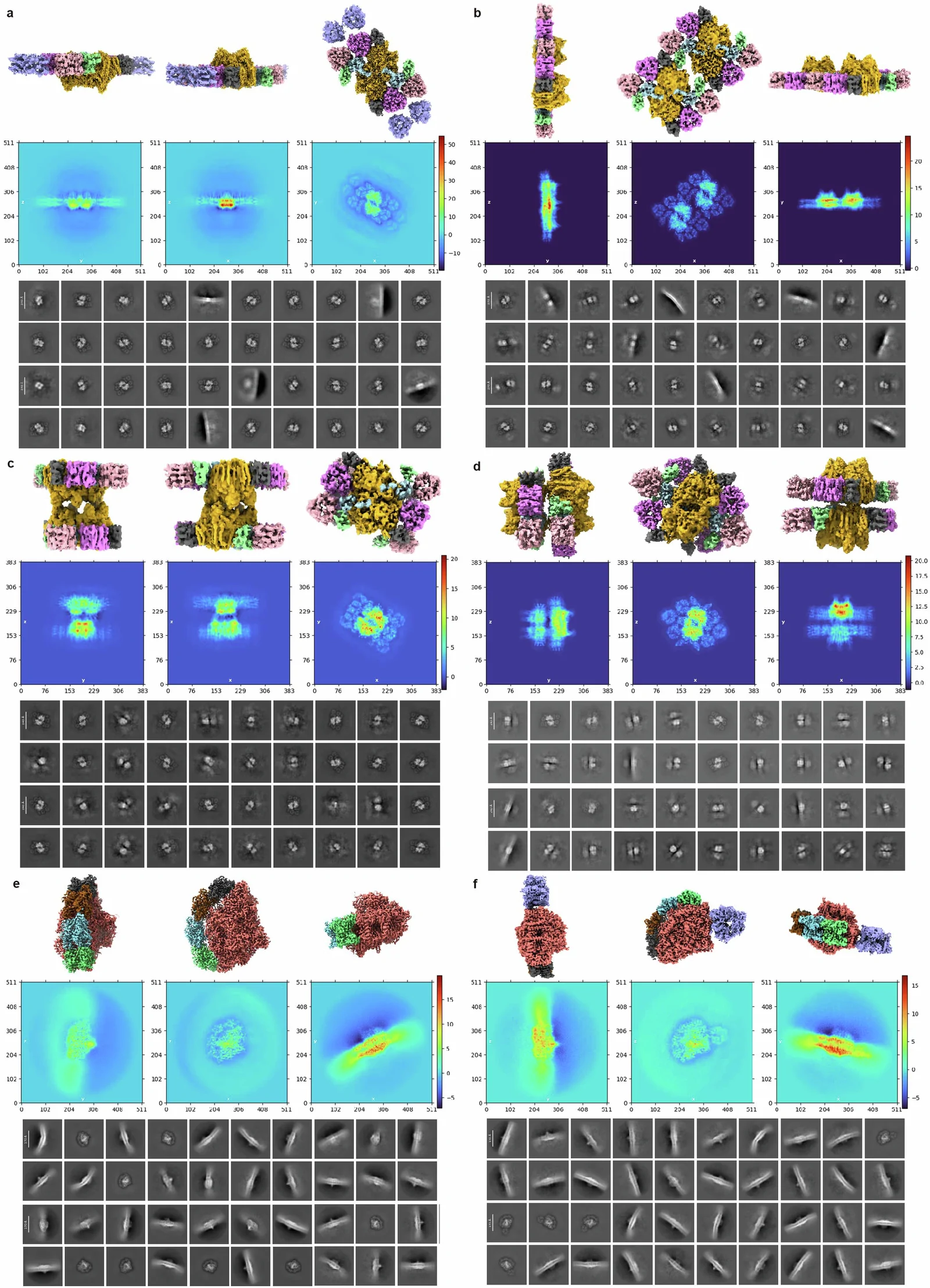
Assessment of partial structural states of PSI and PSII complexes using 2D classification following three-dimensional refinement. **a**-**f**, Density maps (top) shown from three representative views with golden amber and salmon colours representing the densities of reaction centers from PSII and PSI, respectively, the corresponding projections from the same orientations (middle), and representative 2D class averages (bottom) of (**a**) C_2_S_2_M_2_L_4_ PSII-LHCII (EMD-68060), (**b**) side-by-side PSII-LHCII dimer (EMD-81438), (**c**) TL dimer (EMD-81447), (**d**) TS dimer (EMD-68048), (**e**) PSI-LHCI (EMD-66925), and (**f**) PSI-LHCI-LHCII (EMD-66932) complexes following 3D classification and refinement.

To overcome these limitations, we used in situ cryo-EM single-particle analysis^28^ to directly image *O. sativa* chloroplasts and visualize photosystem supercomplexes in their native membrane environment (Fig. 1, Extended Data Figs. 1 and 2, Supplementary Figs. 1–7, Supplementary Videos 1–4 and Supplementary Tables 1 and 2). This approach reveals the native architecture and higher-order organization of PSII and PSI and shows how their spatial arrangement contributes to thylakoid organization and supports efficient energy conversion in vivo.

## C_2_S_2_M_2_L_4_-type PSII–LHCII architecture

**Fig. 2:**
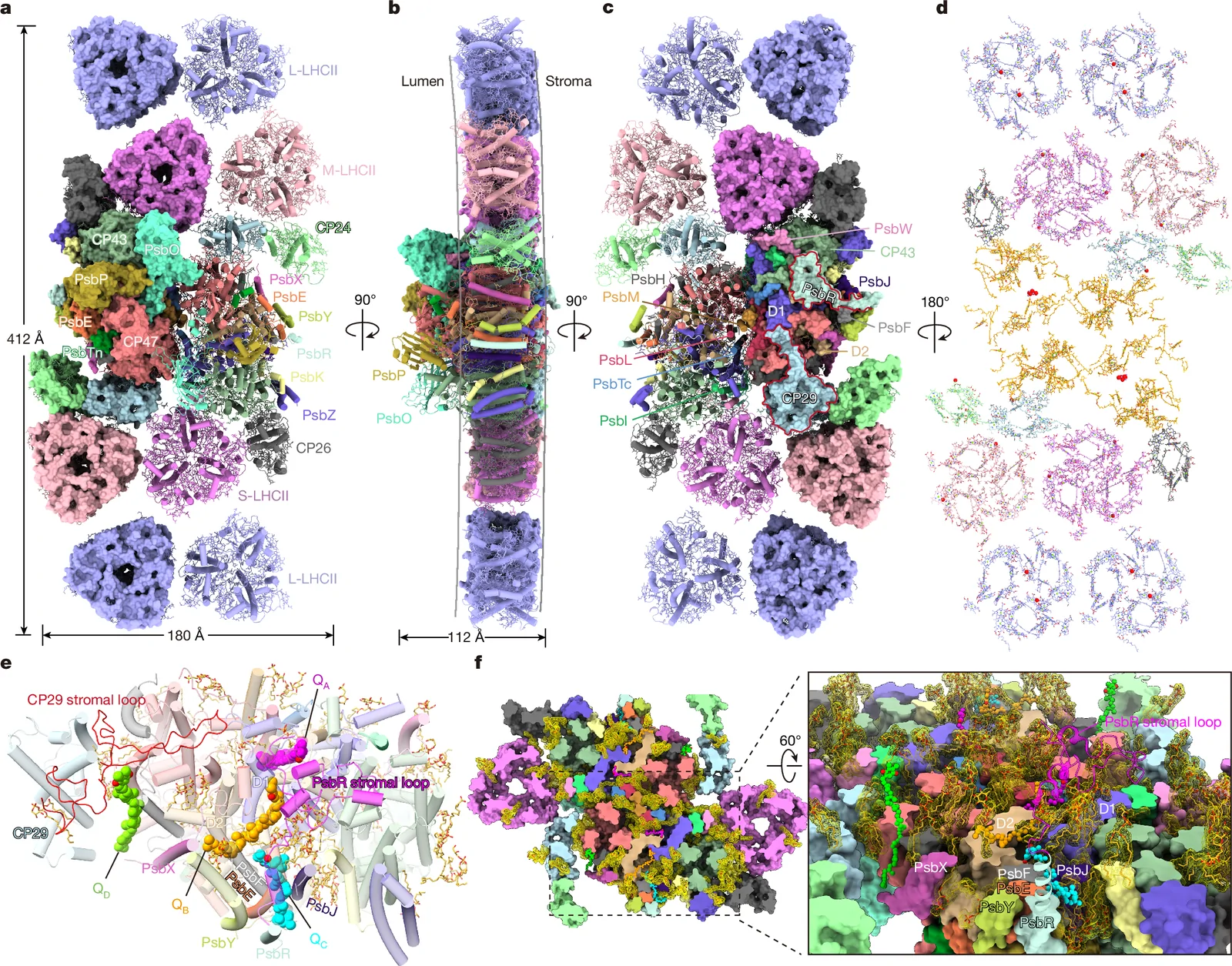
In situ structure of the C_2_S_2_M_2_L_4_-type PSII–LHCII supercomplex. **a**–**c**, Lumenal (**a**), side (**b**) and stromal (**c**) views of the cryo-EM atomic model of the C_2_S_2_M_2_L_4_ supercomplex (PDB 21WV), shown in both surface and cartoon representation, with antenna and core subunits indicated. The L-LHCII regions were fitted with the LHCII models from the PSI–LHCI–LHCII complex according to their distinct triangular densities. Peripheral antenna proteins: CP24 (pale green), CP29 (cyan) and CP26 (dark grey); LHCII trimers are differentially coloured by type: S-trimers (magenta), M-trimers (light pink) and L-trimers (light slate blue). **d**, Lumenal view, highlighting the spatial distribution of the pigments, with the core region rendered in golden amber and other regions maintaining consistent colouring as in **a**–**c**. **e**, Architecture of a promoter core and CP29 from the stromal side, with plastoquinone molecules (Q_A_, Q_B_, Q_C_ and Q_D_) represented as magenta, orange, cyan and green ball sticks, respectively. Protein subunits are depicted as semi-transparent cartoons and coloured as in **a**–**c**. **f**, The interaction interfaces between core subunits and peripheral antennas within the supercomplex, along with lipid molecules occupying the interstitial spaces (viewed from the stromal side). Yellow mesh density maps represent lipid molecules filling the cavities between the subunits.

The in situ structure of the C_2_S_2_M_2_L_4_-type PSII–LHCII supercomplex in *O. sativa* thylakoid is presented in Fig. 2a–c. In addition to the previously characterized core dimer (C_2_) surrounded by 18 peripheral antenna subunits, including two CP26, two CP29, two CP24, two strongly bound LHCII trimers (S-LHCII) and two moderately bound LHCII trimers (M-LHCII)^2^, we identified four loosely bound LHCII trimers (L-LHCII), bringing the total number of peripheral antenna subunits to 30 per supercomplex (Fig. 2a–c and Supplementary Table 1). The local resolution reaches 2.3 Å in the core, 2.6 Å in S-LHCII, 3.0 Å in M-LHCII and 5.0 Å in L-LHCII (Supplementary Fig. 1).

The high-resolution density maps enabled clear visualization of aromatic side chains, chlorophyll rings with central Mg atoms, carotenoids, plastoquinone isoprenoid chains, haem groups, and the Mn and Ca atoms of the Mn_4_CaO_5_ oxygen-evolving cluster (Fig. 1h), enabling us to construct an atomic model of the C_2_S_2_M_2_ complex. For the lower-resolution L-LHCII regions, we fitted the distinct triangular density with the trimeric LHCII model from the PSI–LHCI–LHCII complex, obtaining a complete molecular model of C_2_S_2_M_2_L_4_. This supercomplex measures approximately 412 Å × 180 Å × 112 Å (Fig. 2a,b). In the C_2_S_2_M_2_ region, we resolved 305 chlorophylls, including an additional chlorophyll *a* (Chl *a*) positioned at one side of CP24 proximal to the core region (Supplementary Figs. 8 and 10f), 4 pheophytins, 52 carotenoids and 8 plastoquinone-9 molecules, with 4 on each side (Q_A_, Q_B_, Q_C_ and Q_D_) (Fig. 2d,e and Supplementary Table 1). Additionally, we resolved 111 functionally associated native lipids (66 LHG, 23 sulfoquinovosyl diacylglycerol (SQD), 13 DGD, 3 α-linolenic acid (LNL), 5 monogalactosyl-diacylglycerol (MGE) and one phosphatidic acid (3PH) molecule, depicted in Fig. 2f and Supplementary Fig. 9a, b), which stabilize the supercomplex by mediating protein–lipid–protein interactions, creating hydrophobic environments at PL9-binding sites, and participating in hydrogen-bond networks via their polar headgroups (Supplementary Fig. 9c–f).

The native thylakoid membrane exhibits pronounced lumenal curvature—characterized by a concave core region and lumen-tilted M-LHCII trimer—that is preserved in purified *Pisum sativum* C_2_S_2_M_2_-type supercomplexes (Supplementary Fig. 10a–d). The C_2_S_2_M_2_L_4_ supercomplex displays pseudo-twofold symmetry (Supplementary Fig. 10e), with accompanying asymmetry extending to several ligands (Chl *a*, DGD, SQD and LHG), which adopt distinct conformations or are absent in one protomer (Supplementary Fig. 10f–i and Supplementary Figs. 8 and 9).

**Extended Data Fig. 3:**
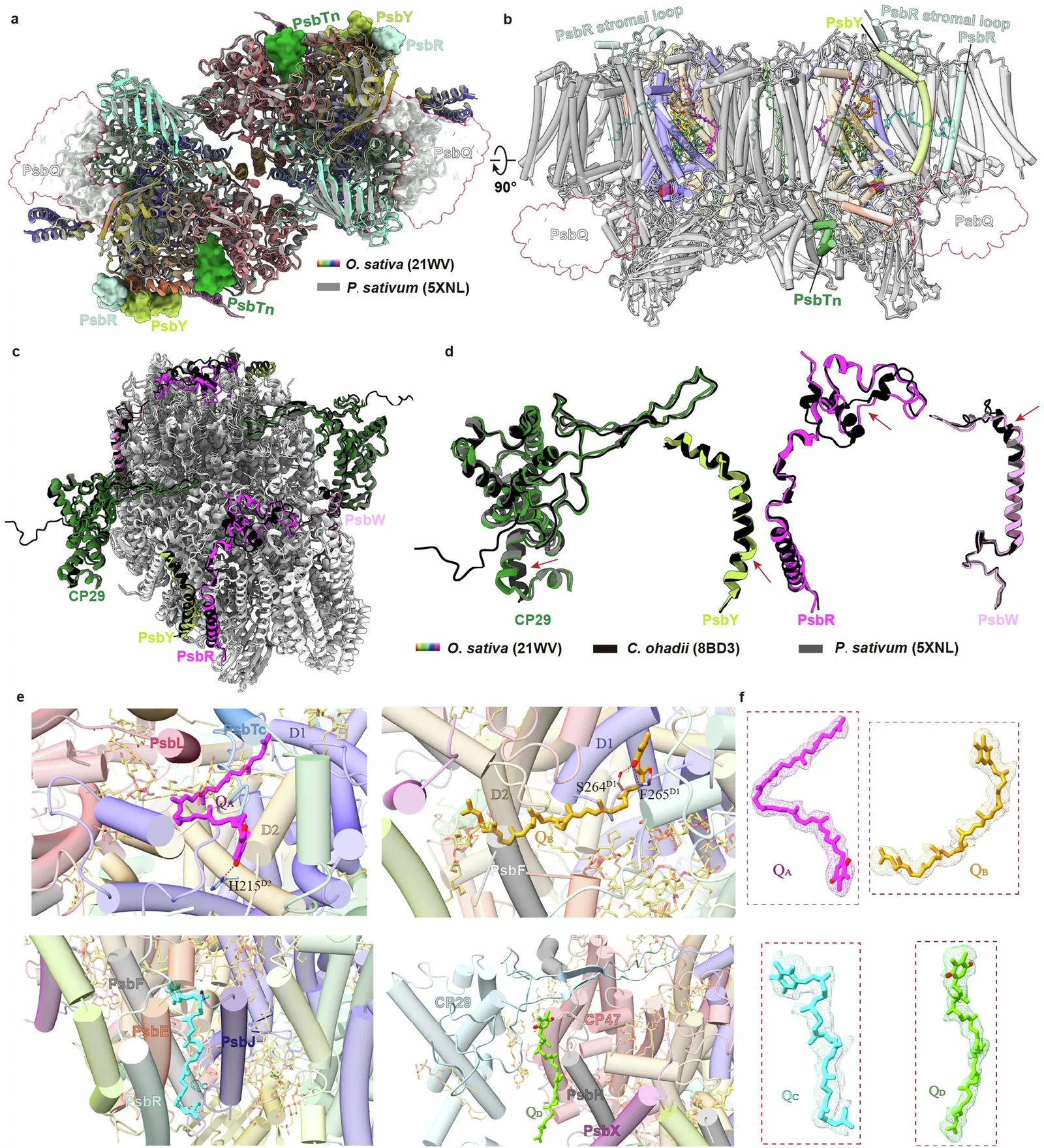
Structural comparison of the core of PSII–LHCII supercomplexes from *O. sativa* and other species. **a**, Luminal view of the structural alignment between the in situ *O. sativa* C_2_S_2_M_2_L_4_ core (PDB 21WV, coloured as in Fig. 2) and the purified *P. sativum* C_2_S_2_M_2_ complex (PDB 5XNL, light gray), aligned on one D1 subunit. PsbQ, present in *P. sativum* but absent in *O. sativa*, is shown as a white transparent surface outlined in red. Extra subunits resolved in *O. sativa* (PsbR, PsbY and PsbTn) are highlighted as solid surfaces. **b**, Side view of the alignment in a, shown as tube helices. In *O. sativa*, only D1, D2, PsbR, PsbY and PsbTn retain colours from a; Other core subunits are shown in dark gray. Features of the *P. sativum* complex are displayed in light gray. **c**, Stromal view of core and CP29 alignment from *O. sativa, C. ohadii* (PDB 8BD3), and *P. sativum*. PsbR and CP29 from *O. sativa* are highlighted in magenta and dark green, respectively; PsbY and PsbW are coloured as in Fig. 2. Other subunits are shown in white cartoon. Corresponding subunits from *C. ohadii* and *P. sativum* are displayed in black and dark gray, respectively, with remaining subunits in white. The *O. sativa* and *C. ohadii* structures were aligned using PsbR. Due to the absence of PsbR in *P. sativum*, its structure was aligned using the D1 subunit from one promoter of *O. sativa*. **d**, Close-up comparison of PsbR, PsbY, CP29, and PsbW from the three species. **e**, **f**, Close-up views of the plastoquinone-binding sites and their corresponding density maps. Density meshes are coloured to match each plastoquinone. Both Q_B_ and Q_C_ sites connect to an internal cavity lined with lipids.

Comparative analysis of the core regions of the in situ *O. sativa* C_2_S_2_M_2_L_4_-type supercomplex and its purified *P. sativum* counterpart reveals clear differences in subunit composition (Extended Data Fig. 3a,b). The *O. sativa* core lacks the oxygen-evolving complex (OEC) extrinsic subunit PsbQ but contains PsbTn^6^ embedded in a groove formed by CP47, D2 and PsbE. Furthermore, the in situ architecture resolves the detergent-labile transmembrane subunits PsbY and PsbR, localized adjacent to cytochrome *b*_559_ (PsbE–PsbF) (Fig. 2). On the stromal side, the N-terminal loop of PsbR forms a shield-like structure spanning D1, D2 and CP43, arching over the stromal side of the plastoquinone exchange channel (Fig. 2e,f and Extended Data Fig. 3b–d). We also identified additional densities interacting with the PsbR loop (Supplementary Fig. 4a), suggesting stromal contacts that may contribute to grana stacking. CP29 loop conformations are conserved between native *O. sativa* and detergent-isolated *P. sativum* structures (PDB 5XNL) (Extended Data Fig. 3c,d). Below the PsbR stromal loop (Fig. 2e,f), a lipid-rich region demarcated by PsbJ—a subunit that is absent in many detergent-isolated structures (for example, PDB 5MDX, 6YP7 and 6KAD)—highlights the structural dependence on a native lipid environment for complex stability.

The electron transfer chain architecture is conserved between the structures of the *O. sativa* and *P. sativum* complexes, with no major changes in the arrangement of the D1–D2 cofactors, and the Q_A_ and Q_B_ sites (Supplementary Fig. 11). However, the *O. sativa* structure also contains a Q_C_ molecule within a lipid-rich channel formed by PsbR, PsbJ, PsbE and PsbF (Fig. 2e,f and Extended Data Fig. 3e,f). Its density is weaker than that of the other plastoquinone molecules, indicating substantial local flexibility or lower occupancy. In addition, we identify Q_D_, a previously uncharacterized transmembrane density. This density was tentatively attributed to a plastoquinone molecule on the basis of its nearly membrane-spanning length, its lack of polypeptide chain characteristics and the excellent fit of a plastoquinone model. It occupies the cleft between CP29 and the core subunits CP47 and PsbH—a region previously proposed as the binding site for PsbS, an essential non-photochemical quenching (NPQ) protein^29^ (Fig. 2e,f and Extended Data Fig. 3e,f). These features are absent from detergent-purified structures and are likely to reflect native quinone-binding and regulatory states in stacked thylakoids.

## Three types of PSII–LHCII dimer in situ

**Fig. 3:**
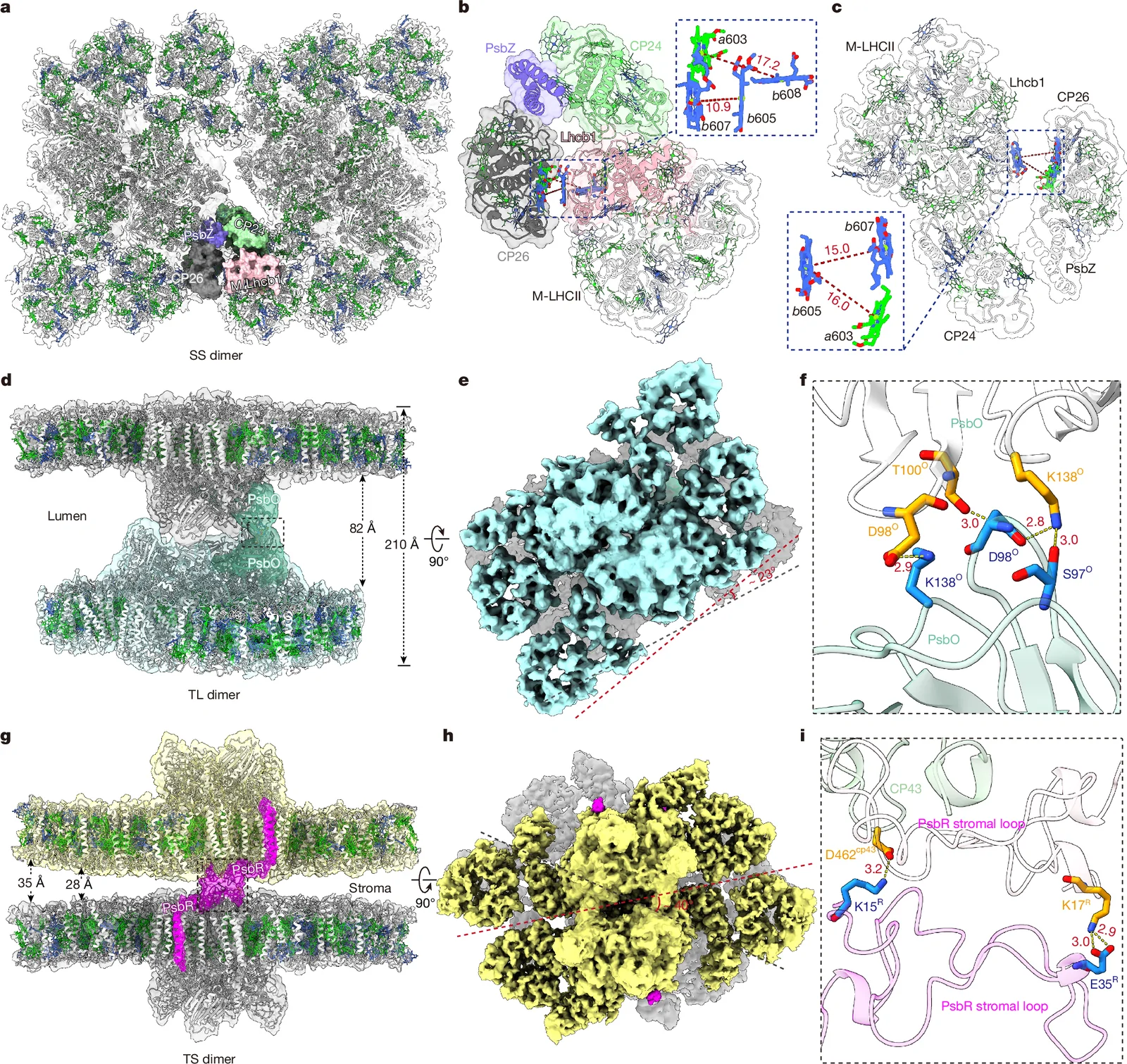
Diversity of native higher-order PSII–LHCII dimeric organization in *O. sativa* thylakoid membranes. **a**, An SS dimer (PDB 27UR, EMDB EMD-81438) forming extended pseudo-*C*_2_ symmetric arrays through lateral contacts between CP26, PsbZ, CP24 and M-Lhcb1. Cryo-EM density shown as white transparent surface. **b**,**c**, SS dimer interfaces. Mg–Mg distances (in Å) between chlorophylls (Chl *a*, green; Chl *b*, blue) in the two PSII units are highlighted, showing contrasting tight (**b**) and loose (**c**) associations between CP26–PsbZ of one monomer and M/Lhcb1 of the adjacent monomer. **d**, A TL dimer (PDB 27UT, EMDB EMD-81447) with OECs from opposing membranes forming PsbO–PsbO (turquoise) bridging interactions at one side. Cryo-EM densities for each PSII are shown as grey and cyan transparent surfaces. **e**, The PSII units within the TL dimer stack co-facially with long axes inclined approximately 23°. **f**, The TL dimer interface. Electrostatic interactions and hydrogen bonds (yellow dashes) between PsbO subunits (turquoise; indicated by superscript O) mediate close interaction, representing a favourable physicochemical interface rather than uniquely determined atomic coordinates. The opposite PsbO–PsbO pair remains spatially separated. Distances indicated in Å. **g**, A TS dimer (PDB 21WH, EMDB EMD-68048) mediated by PsbR (magenta) stromal loops, forming a curved stromal interface with uneven gaps (approximately 35 Å between M-LHCII trimers; approximately 28 Å between CP24 and S-LHCII). Cryo-EM densities for each PSII shown as yellow and grey transparent surfaces. **h**, The two PSII complexes within the TS dimer stack co-facially with long axes inclined about 39°. **i**, The TS dimer interface. PsbR (indicated by superscript R) stromal loops form a face-to-face interface stabilized by electrostatic interactions (yellow dashes), representing a favourable physicochemical interface rather than uniquely determined atomic coordinates. Opposing PsbR loops exhibit spatial offset with no obvious interactions. Distances indicated in Å.

**Extended Data Fig. 4:**
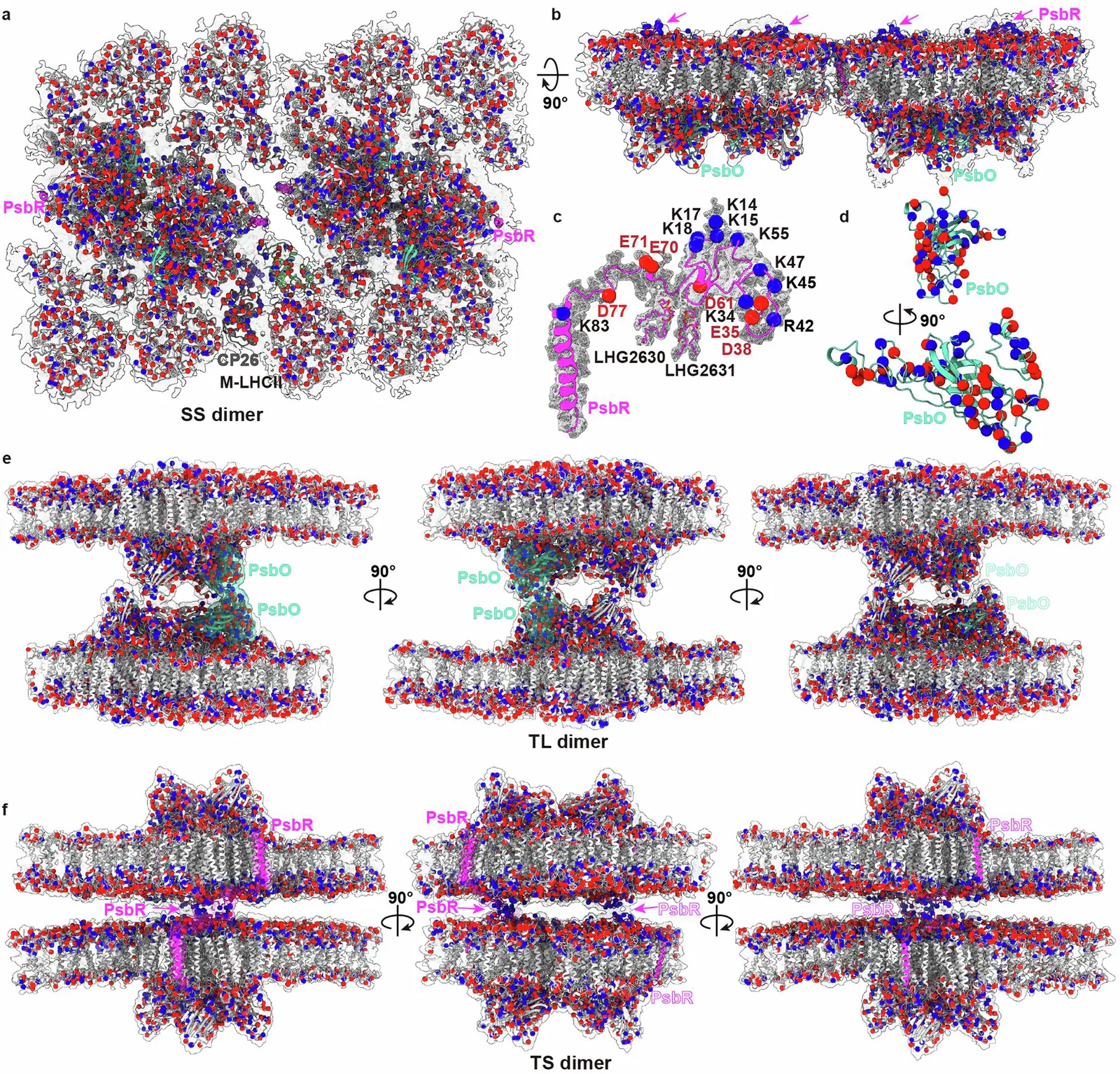
Architectural patterns and distribution of charged residues in diverse higher-order PSII-LHCII dimers of thylakoid membranes. **a**,**b**, The SS dimer architecture and charged residue distribution (PDB 27UR, EMD-81438). Top view from the stromal side (a) and side view from the membrane plane (b) of the lateral dimer. Cα atoms of positively charged basic residues (lysine and arginine) and negatively charged acidic residues (aspartate and glutamate) are shown as blue and red spheres, respectively. Subunits involved in dimer interface formation are labeled. The protruding stromal loops of PsbR forms a positively charged planar projection, indicated by a magenta arrow. **c**,**d**, Spatial architecture and charged residue distribution of the PsbR (c) and the PsbO (d) subunits involved in dimer stacking. The density map (gray mesh) is shown for PsbR. **e**, Architecture and charged residue distribution of the TL dimer (PDB 27UT, EMD-81447). **f**, Architecture and charged residue distribution of the TS dimer (PDB 21WH, EMD-68048).

In addition to the C_2_S_2_M_2_L_4_ supercomplex, we resolved three distinct dimeric configurations of PSII–LHCII (Fig. 3, Extended Data Fig. 4 and Supplementary Figs. 2, 3 and 4a–p): (1) a side-by-side (SS) dimer; (2) a trans-lumenal (TL) dimer; and (3) a trans-stromal (TS) dimer.

The SS configuration (Fig. 3a), determined at 3.5–5.5 Å resolution (Supplementary Fig. 2), forms extended pseudo-*C*_2_ symmetric arrays through lateral association^30^. The interface is asymmetric (Fig. 3a–c and Extended Data Fig. 4a,b): On one side, tight association occurs between CP26–PsbZ of one monomer and CP24–Lhcb1 within M-LHCII (M/Lhcb1) of the adjacent monomer (Fig. 3b), with chlorophyll pair Chl *b*607 and Chl *a*603 of CP26 within 10.9 Å and 17.2 Å of Chl *b*605 and Chl *b*608 of M/Lhcb1, respectively. The opposite interface maintains only loose spatial proximity between the same subunits, with distances of 15 Å and 16 Å between Chl *b*607 and Chl *a*603 of CP26 and Chl *b*605 of M/Lhcb1 (Fig. 3c). However, our calculations indicate that this organization does not facilitate EET between complexes (see below).

The TL dimer exhibits a novel organization in which PsbO subunits of the OEC from opposing membranes form bridging interactions within the lumen (Fig. 3d). This OEC-mediated dimer stacks two PSII–LHCII supercomplexes co-facially, with their planes parallel but their long axes offset by around 23° (Fig. 3e and Extended Data Fig. 4e). The resulting lumenal gap (approximately 82 Å) corresponds to the gap observed for light-adapted plants^31^. The distance between outer surfaces of the PSII–LHCII complexes is about 210 Å, consistent with the thickness of the thylakoid membrane determined by cryo-electron tomography (cryo-ET)^4^.

In the TS dimer configuration, PSII–LHCII supercomplexes form stromal-side contacts mediated by PsbR loop regions (Fig. 3g), as suggested by cross-linking experiments^32^. One pair of loops creates a bridging interaction while a second pair approaches each other without direct contact (Fig. 3g–i and Extended Data Fig. 4f). The non-planar configuration of individual PSII monomers contributes to the formation of a curved stromal interface with uneven gap distances of about 35 Å between M-LHCII trimers, and about 28 Å between CP24 and S-LHCII, consistent with the intermembrane spacing previously measured by cryo-ET^4^. The long axes of the two supercomplexes are inclined at an angle of approximately 40° (Fig. 3h). This is clearly different from the results based on purified complexes, where PSII–LHCII always appears as a perfectly overlapping sandwich.

All of these dimer types show substantial variability in angles and distances between the PSII–LHCII supercomplexes (Supplementary Fig. 12). Consistently, focus refinement results show that when the focus is placed on one PSII–LHCII complex, the other appears blurred (Supplementary Figs. 2h,i, 3f and 4f).

## PSII–LHCII-based stacking network

**Fig. 4:**
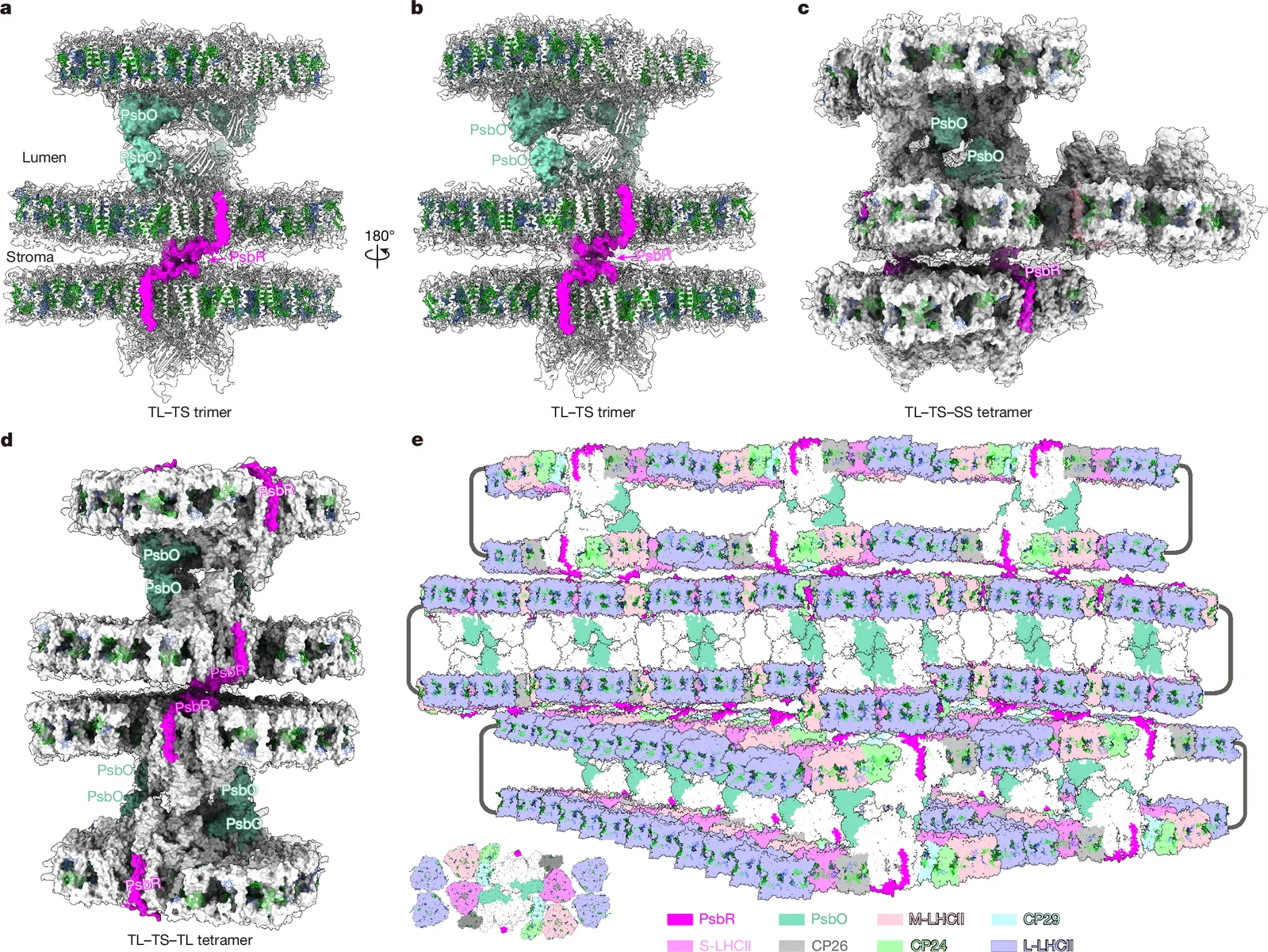
Higher-order assembly patterns of PSII–LHCII supercomplexes into a macroscopic network within thylakoid membranes. **a**,**b**, Front and back views of the TL–TS trimer (PDB 21WI, EMDB EMD-68049) spanning both lumen and stromal spaces, assembled via unilateral PsbR–PsbR stromal interactions and PsbO–PsbO lumenal bridges. The cryo-EM density map is displayed as a white surface with 80% transparency. **c**, The TL–TS–SS tetramer (PDB 21WG, EMDB EMD-68047) is formed through both vertical (inter-layer) and horizontal (intra-layer) connections. **d**, The TL–TS–TL tetramer (PDB 21WD, EMDB EMD-68043) is constructed by linking two TS dimers via a trans-stromal interaction. **e**, Proposed schematic model of grana architecture shaped by C_2_S_2_M_2_L_4_ supercomplexes. Their distinct assemblies modulate local membrane curvature and lumenal spacing.

Our structures reveal that PSII–LHCII supercomplexes within stacked grana membranes can assemble into higher-order structures, which may form a PSII–LHCII-based stacking network and thereby contribute to grana stacking. This network comprises at least three distinct classes of assemblies that bridge adjacent membrane layers and extend laterally within the membrane plane. These include: (1) an asymmetric trans-lumenal/trans-stromal trimer (TL–TS trimer) that extends a TL dimer through a stromal interaction (Fig. 4a,b and Supplementary Fig. 4q–y); (2) a mixed trans-lumenal/trans-stromal and side-by-side tetramer (TL–TS–SS tetramer), in which a PSII unit is laterally associated with the TL–TS trimer (Fig. 4c and Supplementary Fig. 5); and (3) A bis-trans-lumenal/trans-stromal tetramer (TL–TS–TL tetramer) in which two TL dimers are connected through stromal contacts (Fig. 4d and Supplementary Fig. 6). Crucially, all these structures—dimers, trimers and tetramers—bridge adjacent membrane layers and extend laterally, representing not conventional oligomers but local patterns within a larger ordered architecture.

The structures of these higher-order assemblies were determined at an average resolution of around 4 Å (reaching 3.5 Å locally) (Supplementary Figs. 4w,x, 5p,q and 6o,p). Using our atomic model of PSII–LHCII, we built molecular models for these higher-order complexes and elucidated the key interactions that stabilize each assembly node. A dual-interface architecture forms between adjacent thylakoid membranes. This architecture comprises two asymmetric contact modules: (1) of the two pairs of stromal loops from PsbR subunits, only one pair engages in tight interactions; (2) similarly, of the two pairs of PsbO subunits, only one pair forms tight contacts, while the other remains relatively loose. This specific ‘one-tight–one-loose’ asymmetric arrangement aligns with our initial observation that extra density features on both the lumenal and stromal sides of the C_2_S_2_M_2_L_4_-type PSII–LHCII are predominantly attached to a single PSII protomer (Supplementary Fig. 4a and Supplementary Video 1). We propose that such an asymmetric interface could provide a structural basis for the reversible dynamic rearrangement of the thylakoid network in response to varying environmental conditions (for example, light changes), thereby potentially facilitating the modulation of photosynthetic efficiency.

These structural observations strongly suggest that PSII–LHCII supercomplexes are likely to serve as repetitive structural nodes. Through specific, asymmetric interactions, they are poised to assemble into a continuous, tessellated 3D network (Fig. 4e and Supplementary Video 4). This architecture would be competent in transducing mechanical forces across membrane layers and is probably central to providing the structural basis for thylakoid stacking, curvature and long-range order. These findings point to a model in which local protein–protein interactions directly govern the establishment and regulation of global membrane morphology.

## C_2_S_2_M_2_L_4_ PSII EET pathways

Using the C_2_S_2_M_2_L_4_ PSII structure, we calculated excitation energy trapping with a combined modified Redfield and generalized Förster approach^33,34^ (Methods).

**Fig. 5:**
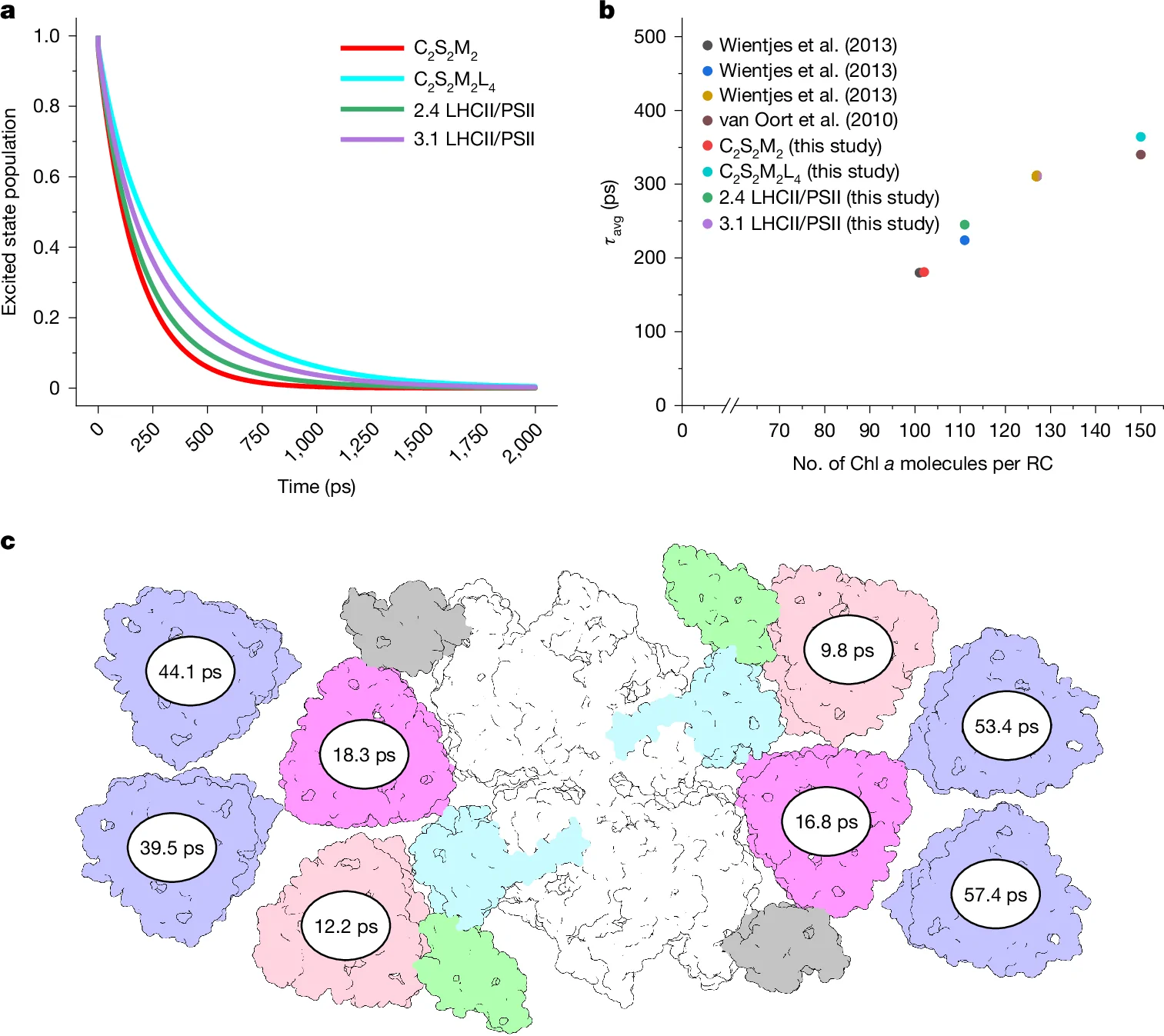
Excitation energy trapping calculations. **a**, Simulated excited state decay curves for the C_2_S_2_M_2_ and C_2_S_2_M_2_L_4_ complexes, and for PSII dimers with on average 2.4 and 3.1 LHCII trimers per PSII reaction centre. **b**, Comparison of experimentally determined PSII lifetimes (*τ*_avg_) for *A. thaliana* PSII particles of various sizes (van Oort et al.^40^) and the current calculation results for the *O. sativa* PSII particles. The datapoint for 3.1 LHCII/PSII (this study) coincides with the data from Wientjes et al.^22^. RC, reaction centre. **c**, Average thermalized depopulation lifetimes for the LHCII complexes in C_2_S_2_M_2_L_4_, coloured as in Fig. 4e.

For the C_2_S_2_M_2_L_4_ complex (Fig. 5a), we obtained an average lifetime of 364.0 ps, corresponding to a quantum efficiency (*Φ*_PSII_) of 83%, which is consistent with experimental values for higher plants^35–37^, including *O. sativa*^38^.

For the C_2_S_2_M_2_ complex, the calculated lifetime was 180.9 ps (Fig. 5a), which matches closely the experimentally determined value for both the isolated complex^39^ and PSII in vivo with approximately two LHCII trimers per reaction centre^22^ (Fig. 5a,b).

As PSII antenna size varies with growth light conditions, comparison with in vivo lifetime measurements across light conditions enables us to relate the calculated lifetimes to specific antenna sizes. The lifetimes for complexes ranging from C_2_S_2_M_2_ to C_2_S_2_M_2_L_4_ closely match those measured in vivo in functional membranes containing from two to four LHCIIs per reaction centre (Fig. 5a,b), spanning high-light- to low-light-adapted states^22,40^. In particular, agreement with lifetimes reported for membranes containing approximately four LHCII trimers per reaction centre^40^ supports C_2_S_2_M_2_L_4_ as representative of a low-light-adapted PSII organization that is conserved across higher plants. Furthermore, our results rationalize the role of L-LHCII trimers in antenna size regulation.

Consistent with in vivo spectroscopic data^22,40^, our calculated thermalized excitation energy depopulation times (Fig. 5c and Methods) show that M-LHCII has the fastest depopulation rate (*τ*_out_ = 9.8–12.2 ps), reflecting a strong connection, especially to CP29^41^, followed by S-LHCII (*τ*_out_ = 16.8–18.3 ps), whereas L-LHCII is much slower (*τ*_out_ = 39.5 to 57.4 ps), indicating weaker connectivity. This weak connectivity of L-LHCII may facilitate association–dissociation with the C_2_S_2_M_2_ complex, enabling modulation of the antenna size under changing light conditions, albeit at the cost of a reduced energy transfer efficiency.

**Extended Data Fig. 5:**
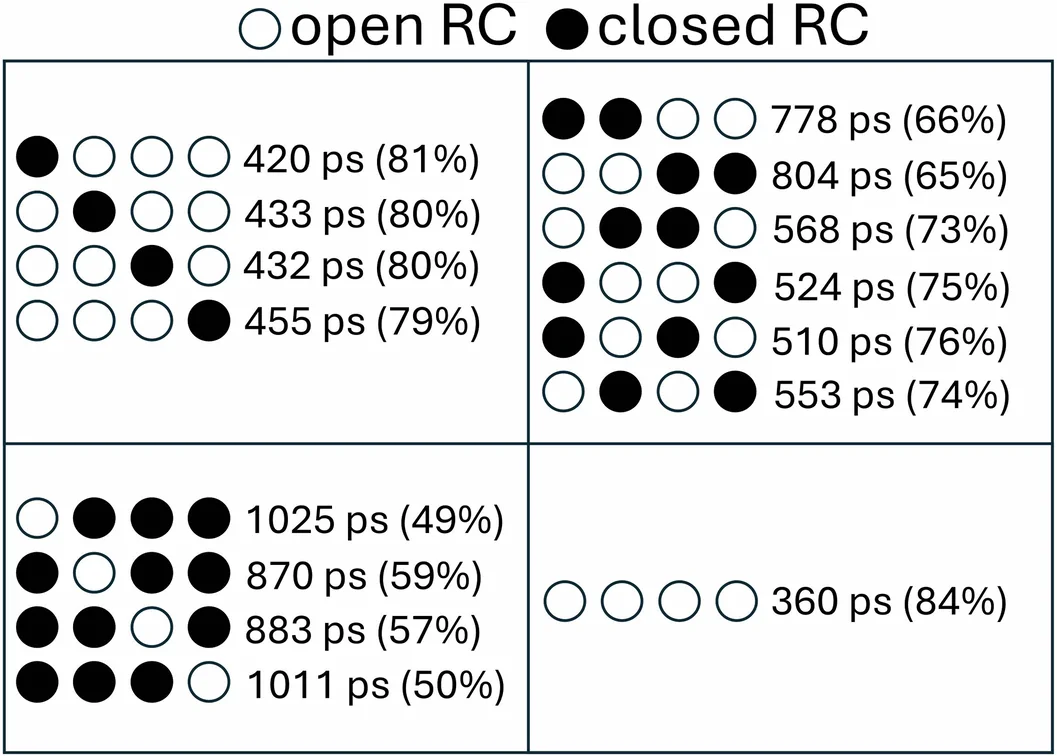
Average PSII lifetimes for the PSII side-by-side configuration in various configurations of open/closed RC’s. The open circles indicate that a perfect photochemical trap was connected to all of the RC pigments of the corresponding PSII monomer, whereas a closed circle indicates that access to the trap is blocked from that RC. Average excited state lifetimes are reported for each of the configurations with their associated Φ_PSII_ in brackets. Closing of one of the four RCs in C_4_S_4_M_4_L_8_ increased the lifetime to ~430 ps, yielding a Φ_PSII_ of around 80%. Closing two RC rises the lifetime to ~550 ps, where there is one open RC in each dimer, and to ~800 ps when the two closed RCs are on the same dimer, illustrating that it is far more unlikely for an excitation to cross over to another PSII dimer. When three out of four RCs are closed, then the lifetime increases to around 1 ns and the Φ_PSII_ drops to 0.5–0.6.

In addition, the PSII SS dimer structure (Fig. 3a) enabled us to evaluate the energetic connectivity between neighbouring PSII complexes. The trapping time of the SS structure (360.0 ps) was nearly identical to that of the C_2_S_2_M_2_L_4_ particle (364.0 ps), indicating that under ‘open’ reaction centre conditions the side-by-side organization does not enhance trapping. Nonetheless, the side-by-side organization could, in principle, allow excitation energy to reach an open reaction centre when others are closed. To test this possibility, we simulated scenarios in which access to specific reaction centres was selectively blocked (Extended Data Fig. 5). The results show that excitations are readily shared within a PSII dimer but rarely cross into a neighbouring one, indicating that the side-by-side organization does not support efficient inter-dimer EET.

In the TS assembly, the minimal distance between chlorophylls across layers is approximately 52 Å, too large to support EET between layers, consistent with experimental observations^42^. The TL assembly shows even greater separation. Thus, both TS and TL assemblies are unlikely to mediate inter-layer energy transfer, suggesting that their primary role is architectural, supporting membrane organization and grana stacking rather than facilitating excitation exchange between layers.

## PSI–LHCI and PSI–LHCI–LHCII in situ

**Fig. 6:**
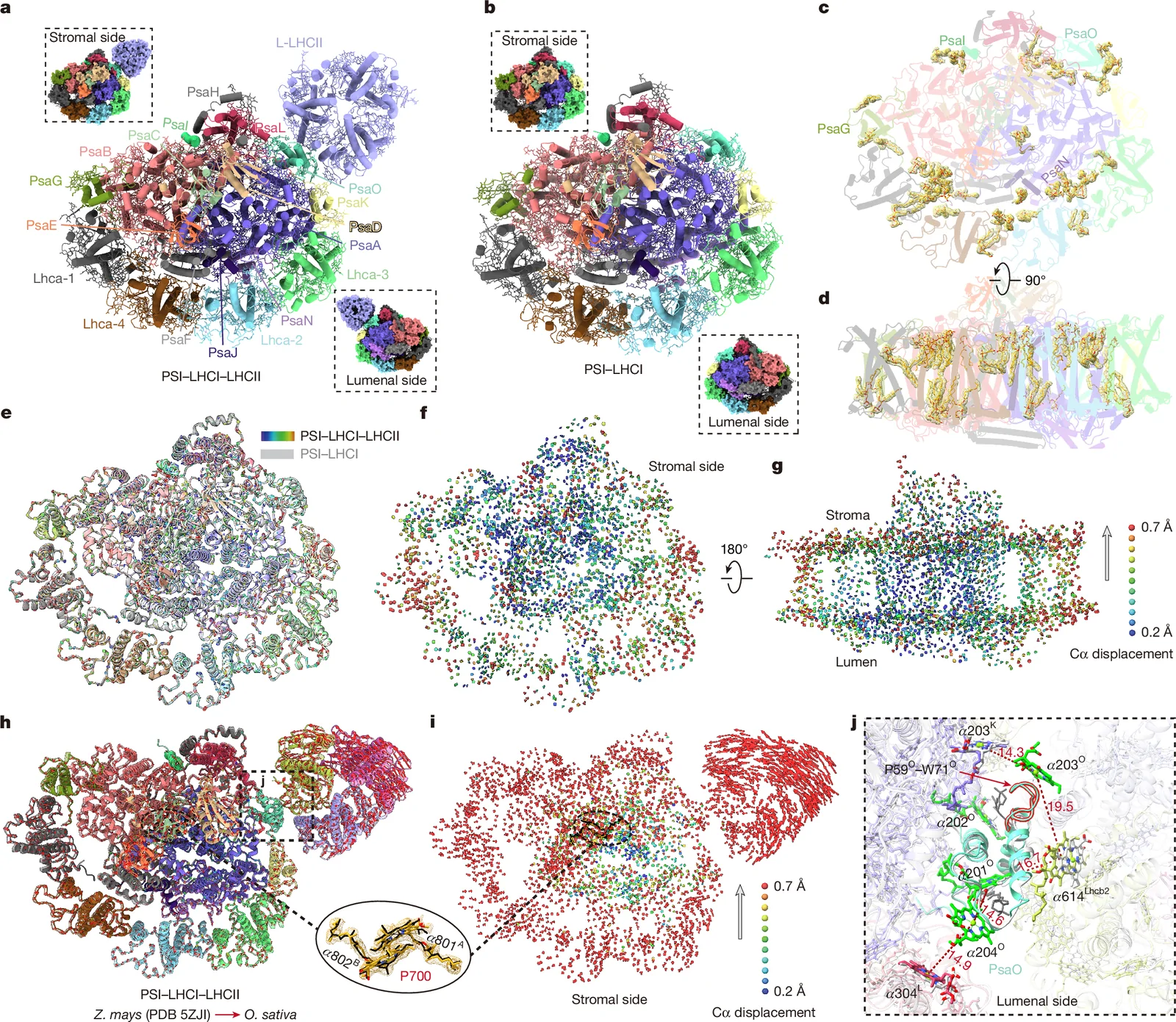
In situ architectures of PSI–LHCI–LHCI and PSI–LHCI reveal LHCII-induced conformational dynamics and features distinct from purified PSI–LHCI–LHCII. **a**, PSI–LHCI–LHCII (PDB 9XJ9) from the stromal side. Core, LHCI and LHCII shown as coloured cartoons. Surface views (dashed outlines) show stromal (top left) and lumenal (bottom right) features. **b**, PSI–LHCI (PDB 9XJ1) from the stromal side using the same scheme. **c**,**d**, The native lipid network at protein–protein interfaces. Lipids (orange sticks) with density maps (yellow mesh), viewed from stromal (**c**) and perpendicular (**d**) sides. **e**–**g**, Asymmetric conformational shifts upon LHCII association comparing in situ PSI–LHCI–LHCII (coloured) and PSI–LHCI (light grey), aligned on PsaA, viewed from stromal (**e**,**f**) and perpendicular (**g**) sides. **f**,**g**, Dot colours indicate displacement magnitudes on Cα atoms. **h**,**i**, Comparison of in situ *O. sativa* (coloured as in **a**) and purified *Z. mays* (PDB 5ZJI, semi-transparent) complexes, aligned on P_700_ (PsaA Chl *a*801 and PsaB Chl *a*802). **h**, P_700_ shown as sticks: orange (*O. sativa*), black (*Z. mays*), enclosed by black dashed ellipse (enlarged in inset). *O. sativa* P_700_ density shown as orange mesh. **i**, Displacement (colour scale as in **f**,**g**) from stromal side. **j**, PsaO at PSI core–LHCII interface, as indicated by dashed outline in **h**. In situ *O. sativa* (coloured) versus purified *Z. mays* (white) complexes. *O. sativa* PsaO contains a lumenal loop (Pro59–Trp71); *Z. mays* adopts a helical turn. In addition to Chl *a*201 and 202 in *Z. mays*, there are two chlorophylls (Chl *a*203 and 204) in *O. sativa* PsaO near the loop and adjacent to PsaL. Red dashed arrows indicate potential energy transfer.

**Extended Data Fig. 6:**
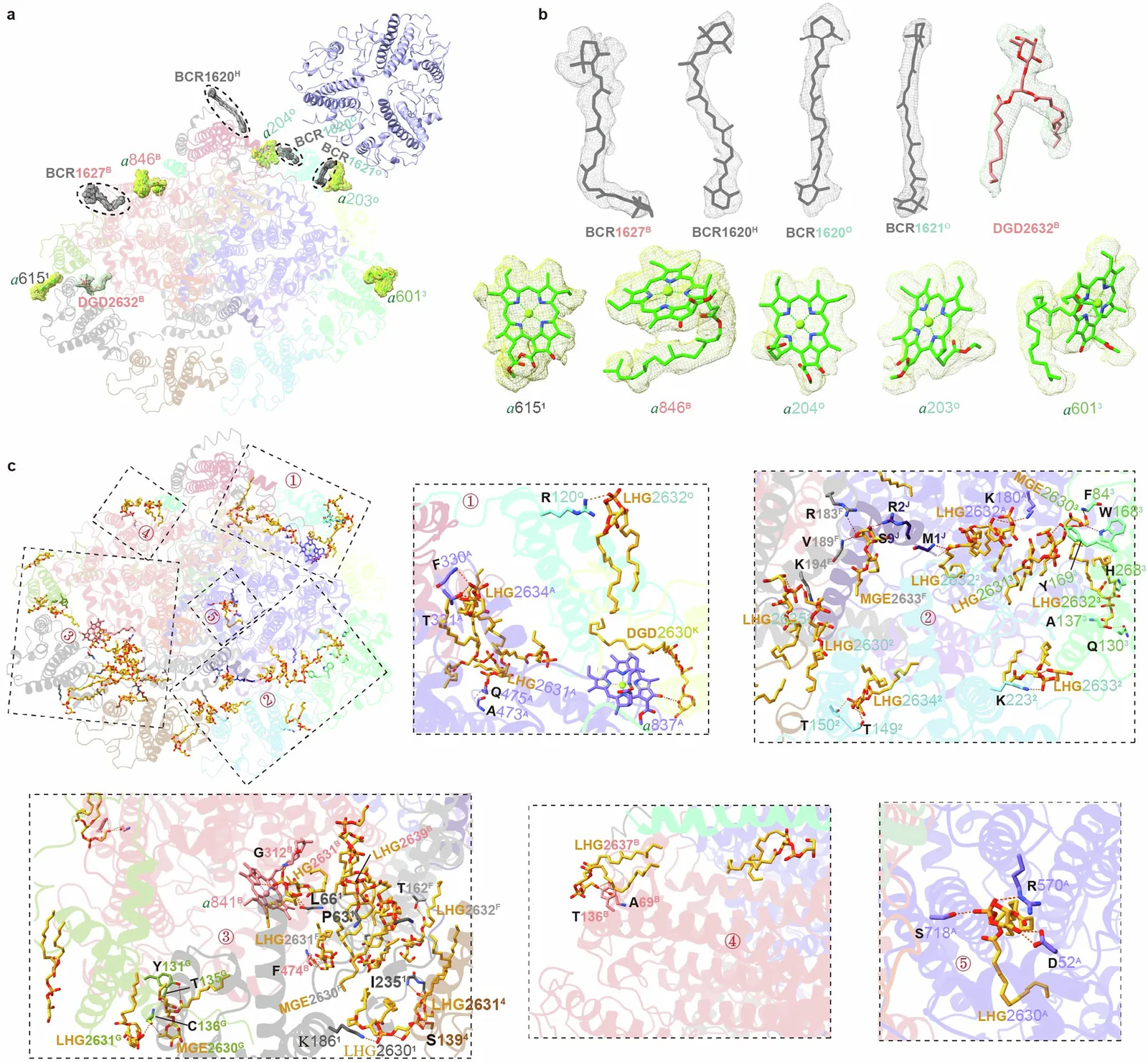
Structural localization of newly identified cofactors and the functional lipid network in the *O. sativa* PSI-LHCI-LHCII supercomplex. **a**, Overall view of the supercomplex (PDB 9XJ9) from the stromal side with protein subunits rendered as cartoons at 60% transparency. Colouring of the PSI core, LHCI, and LHCII is consistent with Fig. 4. Newly identified cofactors are highlighted against the previously published *Zea mays* PSI-LHCI-LHCII structure (PDB 5ZJI): 4 BCR molecules (grey sticks) with their corresponding density map (grey mesh), 5 Chl *a* molecules (green sticks) with density map (yellow mesh), and 1 DGD molecule (salmon sticks) with density map (medium aquamarine mesh). Dashed circles indicate the locations of the newly modelled BCRs. **b**, Detailed view of the newly resolved cofactors, showing 4 BCRs, 5 Chl *a* molecules, and 1 DGD, alongside their respective density maps. **c**, Close-up of the lipid network at protein–protein interfaces. Lipids are shown as orange sticks, key interacting residues are depicted as sticks coloured by their parent chain, and hydrogen-bonding interactions are indicated by vivid pink dashed lines.

Our in situ structural analysis reveals the coexistence of PSI–LHCI and PSI–LHCI–LHCII supercomplexes within the *O. sativa* thylakoid membrane. The structures retain all key subunits, including PsaG, PsaI, PsaN and PsaO (Fig. 6a,b), which are frequently lost in purified preparations^43^, and contain additional cofactors compared with the *Zea mays* PSI–LHCI–LHCII structure (PDB 5ZJI), including four β-carotenes, five Chl *a* molecules and one DGD, all of which are located at the periphery of the complex and are typically removed during detergent solubilization, suggesting a comparatively weak association (Extended Data Fig. 6a,b). Consequently, the final model of PSI–LHCI contains 164 chlorophylls and 40 carotenoids, whereas the PSI–LHCI–LHCII supercomplex contains 43 additional chlorophylls (Supplementary Table 2). Notably, we identified a network of native lipids enriched at the interfaces between PSI core and antennae, between adjacent antennae, and around the easily dissociable subunits PsaG, PsaI, PsaN and PsaO (Fig. 6c,d), that act as ‘molecular glue’ by forming extensive hydrophobic interactions with protein subunits (Extended Data Fig. 6c). They not only stabilize the binding interface between the PSI core and the LHCI and LHCII antennas but also reinforce the association of LHCII trimers. This finding provides a mechanistic explanation for the loss of specific subunits and additional LHCII antennae in conventionally purified samples, in which these structural lipids are displaced.

Comparative analysis of our in situ structures of PSI–LHCI–LHCII and PSI–LHCI supercomplexes reveals asymmetric dynamic movements of the LHCI belt and peripheral subunits upon LHCII association (Fig. 6e–g). In addition, the in situ PSI–LHCI–LHCII supercomplex is markedly more compact than the purified *Z. mays* counterpart (PDB 5ZJI), with inward displacement toward the P_700_ reaction centre of all subunits and cofactors (Fig. 6h,i). At the PSI core–LHCII interface, structural comparison identifies a lumenal loop (P59–W71) and two additional chlorophylls (Chl *a*203 and 204) in *O. sativa* PsaO, a feature that is absent in the corresponding helical region of *Z. mays* PsaO (Fig. 6j). Given the high sequence conservation between species (Supplementary Fig. 13), the observed compaction is likely to represent a functional, native configuration that is perturbed upon biochemical isolation.

## PSI–LHCI and PSI–LHCI–LHCII EET pathways

To assess whether the observed movement of the LHCI belt influences excitation energy exchange with the PSI core, we performed Förster resonance energy transfer (FRET) calculations using the framework presented in ref. ^1^ (Methods). There are four chlorophyll connection zones between LHCI and the PSI core, one for each Lhca subunit. For the Lhca1–Lhc4 dimer, the main pathways have been identified as *a*606(1) → *a*1233(B), *a*603(1) → *a*1802(B) and *a*616(1) → *a*1701(F), with calculated energy transfer time constants of 7.2 ps, 2.7 ps and 0.7 ps, respectively^44^. Our FRET calculations yield similar time constants (Extended Data Table 1), and do not vary substantially between the two extreme positions of the LHCI belt.

For the Lhca2–Lhca3 dimer, connections to the core involve *a*601(2) → *a*201(N), *a*603(2) → *a*201(N), *a*607(3) → *a*817(A) and *a*603(3) → *a*811(A). The calculated FRET rates for the two extreme LHCI belt positions do not differ substantially (Extended Data Table 1), indicating that excitation energy exchange between the LHCI and the PSI core is robust to thermal fluctuations of LHCI, similar to observations for the IsiA → PSI core transfer in *Synechocystis*^45,46^.

The resolved PSI–LHCI–LHCII structure enables us to evaluate the EET pathways between LHCII and the PSI core. The most likely routes involve PsaO chlorophylls: either from the LHCII Chl*a* 610–611–612 cluster to PsaO Chl *a*201, or from the LHCII Chl *a* 613–614 exciton to the newly resolved PsaO Chl *a*204. For optimal site energies of the two PsbO chlorophylls, transfer to PsaO Chl *a*201 is relatively fast (10.0 ps; Supplementary Fig. 14a), whereas transfer to PsaO Chl *a*204 is slow (110 ps; Supplementary Fig. 14b). The effective overall transfer rate from LHCII to the PSI core can thus be approximated from the rate of LHCII Chl *a *610–611–612 → PsaO Chl *a*201 (0.1 ps^−1^) multiplied by the population of this cluster after energy equilibration within the LHCII trimer (13%), yielding 13.4 ns^−1^. This rate is comparable to the rate obtained from time-resolved measurements^47^, confirming that this is the main pathway.

## Discussion

### Native plant photosystem supercomplexes

In vascular plants, PSII and PSI assemble into modular supercomplexes comprising a core, containing the reaction centre, surrounded by multiple LHC antenna proteins, as reported by biochemical and spectroscopic studies^21,40,48^. However, structures obtained from purified plant PSII have predominantly resolved C_2_S_2_M_2_-type supercomplexes, and lack several LHCII trimers inferred from in vivo measurements, suggesting the loss of weakly associated antenna subunits during purification. Using our in situ approach, we resolved a C_2_S_2_M_2_L_4_-type PSII–LHCII supercomplex at high resolution, identifying four previously uncharacterized loosely bound LHCII trimers and thereby revealing a substantially larger antenna than observed in purified samples. Of note, these L-LHCII trimers exhibit substantial flexibility, as reflected by the weaker density and lower local resolution in these regions relative to the PSII core, as well as the S-LHCII and M-LHCII trimers. Under physiological conditions, L-LHCII is inherently dynamic and participates in state transitions^49^ and light adaptation processes^21,22,40^ to regulate excitation energy distribution. Although an average position for these LHCII trimers was defined from the composite maps, their precise orientations and distances are expected to vary in the native membrane. Under the in situ sample conditions used here, a detailed characterization of this conformational heterogeneity is inherently limited—their association is relatively weak and highly dynamic, making it difficult to capture a stable, single conformation. However, EET calculations based on this supercomplex closely match in vivo measured trapping times (Fig. 5), which indicates that although some heterogeneity can be expected in the L-LHCII trimers regions, the current C_2_S_2_M_2_L_4_ organization is a genuine representation of PSII in the membrane. PSI similarly exists in at least two native forms: PSI–LHCI and PSI–LHCI–LHCII.

### Higher-order PSII assembly in thylakoid

Beyond individual photosystems, our in situ experiments reveal how these supercomplexes associate within and between thylakoid layers. In the resolved maps, additional densities adjacent to PSII–LHCII indicate the presence of interacting partner proteins. Extensive cross-classification identifies multiple dimeric, trimeric and tetrameric assemblies formed through side-by-side, trans-lumenal and trans-stromal interactions. On the basis of these observations, we propose that PSII–LHCII supercomplexes form a large-scale skeleton structure, which shapes thylakoid architecture through lumenal and lateral contacts, and stromal contacts mediate the stacking of multiple thylakoids to form grana. These interactions are weak and are therefore disrupted during isolation, highlighting the power of the in situ approach to capture native organizations. The side-by-side, trans-lumenal and trans-stromal organizations identified here provide insight into how photosystem assemblies contribute to thylakoid membrane architecture. Higher-order PSII–LHCII associations are likely to influence local membrane curvature and lumenal spacing, and the native geometry of the thylakoid system in turn constrains the relative orientation, distribution and conformations of individual supercomplexes. SS dimers, which lack strong intermolecular contacts, may primarily optimize packing within the crowded membrane. By contrast, TL and TS dimers adopt ‘close yet separate’ geometries stabilized by electrostatic interaction and localized hydrogen-bond networks involving PsbO, CP43 and PsbR (Fig. 3f,i, Extended Data Fig. 4e,f and Supplementary Fig. 15). We propose that these defined spatial arrangements contribute to the emergence and maintenance of thylakoid membrane morphology.

Some regions of the reconstructed higher-order PSII–LHCII supercomplexes exhibit anisotropic resolution, probably arising from preferential particle orientation inherent to the sample preparation, relative movements among different components and the intrinsic flexibility of the lipid membrane. Nonetheless, the well-resolved overall architectures, particularly in their core regions, provide strict positional constraints for subsequent rigid-body docking, enabling the establishment of reliable models for analysing the stacking modes of PSII–LHCII supercomplexes within the native thylakoid membrane layers.

The molecular basis of grana stacking has long been debated, with LHCII and other antenna complexes proposed to have central roles^50,51^. However, the in situ structures reported here show no direct interactions across the stromal gap involving the N-terminal regions or stromal loops of LHCII or CP29. Notably, these stromal loops adopt conformations similar to those observed in purified complexes^2^. By contrast, the stromal-side interactions observed here involve the stromal loop of the PsbR subunit and are compatible with cross-linking experiments performed in native membranes^32^. Together, these observations indicate that the stacking interactions do not involve direct contacts between stromal antenna regions but instead involve interactions between PSII–LHCII supercomplex cores.

### Functional implications for EET

The calculated trapping times for the C_2_S_2_M_2_ and C_2_S_2_M_2_L_4_ complexes closely match in vivo measurements from *Arabidopsis thaliana* grown under different light conditions, including those in which four LHCII trimers are associated per PSII^22^. This agreement indicates that the C_2_S_2_M_2_L_4_ arrangement resolved in *O. sativa* is likely to represent a general PSII organization in higher plants. The slow excitation exchange involving L-LHCII trimers is consistent with their peripheral position and with spectroscopic data showing that they slow down excitation migration more strongly than S-LHCII and M-LHCII^22,40^. Together with the observation that L-LHCII trimers are also structurally only weakly associated with C_2_S_2_M_2_ and that plants regulate the expression and degradation of Lhcb1 and Lhcb2 during light acclimatization^52^, these findings suggest that L-LHCII trimers are the primary components that are modulated during light acclimation.

Furthermore, the weak connectivity that we observe between adjacent PSII dimers indicates that excitation sharing is efficient only within individual supercomplexes. Moreover, the large stromal separations between trans-stromal complexes preclude energy transfer across grana layers, in agreement with spectroscopic observations^42^. Together, these results highlight how structural organization at different hierarchical levels shapes the functional antenna size and overall photochemical efficiency.

### Perspectives

As anticipated in our previous work on the in situ structures of mitochondrial respiratory supercomplexes^28^, the in situ approach can be extended to structural studies of native membrane protein complexes in various organelles, including chloroplasts. The present study strongly reinforces the capability of in situ cryo-EM for determining high-resolution structures within organellar membranes.

Notably, the mitochondrial respiratory supercomplexes have a molecular mass of approximately 1.8 MDa, whereas in this study, the C_2_S_2_M_2_L_4_ PSII supercomplex is about 2.1 MDa and the PSI–LHCI supercomplex is only about 600 kDa. We anticipate that with continued improvements in algorithms and other cryo-EM techniques, it will become feasible to resolve even smaller and less abundant proteins using this in situ approach.

## Methods

### Preparation of *O. sativa* chloroplasts and cryo-EM grids

Young, nonwilted *O. sativa* leaves were collected and washed briefly in cold water. Chloroplasts from *O. sativa* leaves were prepared as described previously^53^. The isolated chloroplasts were then resuspended in a solution containing 150 mM NaCl. The optical density of the suspension was adjusted to 0.8 at 600 nm for subsequent cryo-EM grid preparation.

For cryo-EM sample preparation, 3 μl of the chloroplast suspension was applied to a freshly glow-discharged 300-mesh Quantifoil R 2/1 gold grid and blotted for 6–8 s at 4 °C and 95% humidity in a Leica GP2 plunge freezer. Grids were flash-frozen in liquid ethane and stored in liquid nitrogen until data collection.

### Single-particle cryo-EM data collection

During data collection we manually selected grid regions with ice of intermediate thickness: sufficiently thin to provide adequate contrast for subsequent analysis, yet not so thin that the chloroplasts, particularly the grana membrane stacks, might become overly compressed. These regions preserved the chloroplast ultrastructure as much as possible, thereby maintaining the native local membrane organization and microenvironment of the grana. Automated data collection was performed on a Titan Krios microscope (Thermo Fisher), equipped with a Gatan K3 Summit direct electron detector operating at 300 kV at 0.41 Å per pixel in superresolution mode, using EPU software (v2.7; Thermo Fisher Scientific). Each image was composed of 40 individual frames with an exposure time of 1.9 s and a dose rate of 28.9 electrons per second per Å^2^. Defocus parameters ranged −1.4 to −2.4 μm. Details of the data collection are summarized in Supplementary Tables 3–6.

### Cryo-EM data preprocessing, particle selection and sorting strategy

All cryo-EM datasets were subjected to motion correction and contrast transfer function (CTF) estimation in cryoSPARC. To overcome the challenges of low signal-to-noise ratios and a densely crowded macromolecular environment, we utilized an iterative particle selection and sorting strategy, following an approach previously established for in situ analysis of mammalian mitochondrial supercomplexes^28^ (Extended Data Fig. 1). Further procedural details are described in the Supplementary Information.

### Initial 3D reconstruction of PSI–LHCI and PSII–LHCII

Through comprehensive 2D analysis, we identified regions exhibiting unique local features indicative of PSII–LHCII or PSI–LHCI. Specifically, the PSII supercomplex appears in 2D classification as parallelogram-shaped particles, characterized by an electron-dense central domain and lateral ring densities assigned to LHCII (Extended Data Fig. 1c,d,f). Similarly, fan-shaped signals observed in other regions suggest the likely presence of a core PSI–LHCI supercomplex (Extended Data Fig. 1c–e). Particles displaying PSII- or PSI-like features were separately merged and subjected to initial 3D reconstruction using ab initio reconstruction in cryoSPARC, generating four initial maps. Among these, one map exhibited clear structural features of either PSI or PSII, whereas the remaining maps showed no discernible protein-like features. These initial maps were used as references for subsequent 3D classification, followed by local refinement and focused classification. Following this, the best maps of PSI–LHCI and PSII–LHCII were selected to guide template-based particle picking for further classification (Extended Data Fig. 1h) and refinement, including global and local CTF refinements using the default parameter settings in cryoSPARC. Further details regarding PSII orientation constraints and heterogeneity handling are provided in the Supplementary Information.

### Cross-classification of multiple supercomplexes

Additional circular densities corresponding to LHCII were observed around the reconstructed PSI–LHCI and PSII–LHCII maps, suggesting the presence of further bound LHCs. We subsequently expanded the particle boxes and carried out additional 3D classification. To enhance classification accuracy and facilitate high-resolution refinement, cross-classification was performed as described in ref. ^28^. Further details regarding the identified supercomplexes, validation strategies, PSII dimer heterogeneity quantification and model building are provided in the Supplementary Information.

### Energy transfer calculations

EET dynamics were calculated using a combined modified Redfield and generalized Förster (mR/gF) approach^34^ based on semi-empirical Hamiltonians, the TrESP (transition charges from electrostatic potentials) method, and the resolved cryo-EM structures. Detailed procedures for the energy transfer calculations—including those for C_2_S_2_M_2_L_4_, C_2_S_2_M_2_-C_2_S_2_M_2_L_4_ Intermediates, LHCII-PsaO, PSI-LHCI, and LHCII thermalized depopulation rates—are provided in the Supplementary Information.

### Reporting summary

Further information on research design is available in the Nature Portfolio Reporting Summary linked to this article.

## Online content

Any methods, additional references, Nature Portfolio reporting summaries, source data, extended data, supplementary information, acknowledgements, peer review information; details of author contributions and competing interests; and statements of data and code availability are available at https://doi.org/10.1038/s41586-026-10847-3.

## Supplementary information

Supplementary Information Supplementary Methods, Supplementary Figs. 1–15, Supplementary Tables 1–8 and legends to the Supplementary Video files

Reporting Summary

Peer Review file

Supplementary Video 1 In situ structure of the C_2_S_2_M_2_L_4_-type PSII–LHCII supercomplex.

Supplementary Video 2 In situ structure of the PSI–LHCI–LHCII supercomplex.

Supplementary Video 3 Structural characterizations of three distinct in situ PSII–LHCII dimers.

Supplementary Video 4 Proposed schematic model of grana architecture shaped by C_2_S_2_M_2_L_4_ supercomplexes.

## Data Availability

The coordinates and cryo-EM density maps generated in this study have been deposited in the Protein Data Bank (PDB) and Electron Microscopy Data Bank (EMDB), respectively. Accession codes are as follows. In situ structure of the C_2_S_2_M_2_L_4_-type PSII–LHCII supercomplex (composite): PDB 21WV, EMDB EMD-68060; C_2_S_2_M_2_L_4_-type PSII–LHCII core (protomer 1 and 2): PDB 9XK3 and 9XK4, EMDB EMD-66946 and EMD-66947, respectively; S-LHCII trimer with CP26 (protomer 1 and 2): PDB 9XK6 and 9XK7, EMDB EMD-66949 and EMD-66950, respectively; M-LHCII trimer with CP29/CP24 (protomer 1 and 2): PDB 9XK8 and 9XK9, EMDB EMD-66951 and EMD-66952, respectively; L-LHCII trimers (one side, protomer 1 and 2): EMDB EMD-66971 and EMD-66974, respectively; in situ structure of side-by-side PSII–LHCII dimer (SS dimer): PDB 27UR, EMDB EMD-81438; in situ structure of the trans-lumenal PSII–LHCII dimer (TL dimer): PDB 27UT, EMDB EMD-81447; in situ structure of the trans-stromal PSII–LHCII dimer (TS dimer): PDB 21WH, EMDB EMD-68048; in situ structure of the trans-lumenal/trans-stromal PSII–LHCII trimer (TL–TS trimer): PDB 21WI, EMDB EMD-68049; in situ structure of the mixed trans-lumenal/trans-stromal and side-by-side PSII–LHCII tetramer (TL–TS–SS tetramer): PDB 21WG, EMDB EMD-68047; in situ structure of the bis-trans-lumenal/trans-stromal PSII–LHCII tetramer (TL–TS–TL tetramer): PDB 21WD, EMDB EMD-68043; in situ structure of PSI–LHCI–LHCII supercomplex: PDB 9XJ9, EMDB EMD-66932; and in situ structure of PSI–LHCI supercomplex: PDB 9XJ1, EMDB EMD-66925. All structural models used as initial templates for model building and for comparison with our in situ structures were obtained from the PDB. The following accession codes were used: *P. sativum* C_2_S_2_M_2_ PSII–LHCII (PDB 5XNL), *Lathyrus oleraceus* PSI–LHCI (PDB 6YEZ), *Picea abies* C_2_S_2_ PSII–LHCII (PDB 8C29), *Chlorella ohadii* PSII (PDB 8BD3) and *Z. mays* PSI–LHCI–LHCII (PDB 5ZJI).

## Extended data figures and tables

**Extended Data Table 1:** FRET time constant between the LHCI belt and the PSI core evaluated at the two extremes of the LHCI movement

Chl – Chl transfer	FRET time constant most proximal arrangment	FRET time constant In furthest arrangement
*a*616(1) – *a*1701(B)	0.76 ps	0.94 ps
*a*603(1) – *a*1802(B)	4.9 ps	5.1 ps
*a*601(1) – *a*1233(F)	8.3 ps	7.8 ps
*a*601(2) – *a*201(N)	1.9 ps	4.0 ps
*a*603(2) – *a*201(N)	0.54 ps	0.47 ps
*a*607(3) – *a*817(A)	0.61 ps	0.54 ps
*a*603(3) – *a*811(A)	5.5 ps	6.3 ps

Energy transfer rates were calculated according to the FRET framework as presented in Croce & Amerongen (2020)^1^.

## Code availability

The codes analyze_refine.py and calcu_angles.py, which were used to quantify structural heterogeneity of PSII dimers, are available at https://github.com/JackZhang-Lab/EM-scripts. The code to calculate the mR/gF EET rates is available at https://zenodo.org/records/13346121 (ref. ^54^). The algorithm to calculate the simplified FRET rates is available at https://zenodo.org/records/13283796 (ref. ^55^).
