# Supplementary material for: In situ structures of plant photosystem supercomplexes
Source: Nature. 2026 Jul 29;657(8133):1094–103. doi: 10.1038/s41586-026-10847-3 (PMC13600941; doi:10.1038/s41586-026-10847-3)
Supplement: Supplementary file 1 [file 41586_2026_10847_MOESM1_ESM.pdf]

nature portfolio

https://doi.org/10.1038/s41586-026-10847-3

Supplementary information

In situ structures of plant photosystem supercomplexes

In the format provided by the authors and unedited

Supplementary Information

In situ structures of *plant* photosystem supercomplexes

Jiao Li (李姣)[a,*], Eduard Elias[a], Kai Zhang (张凯)[*], Roberta Croce[*], Jiapeng Zhu (朱家鹏)[*]

[a] These authors contributed equally to this paper.

[*]Correspondence: zhujiapeng@fy.ahmu.edu.cn; r.croce@vu.nl; jack.zhang@ustc.edu.cn; jiao.li@ustc.edu.cn

The role of PL9 in PSII

Plastoquinone (PLQ) functions as an electron carrier between Photosystem II (PSII) and the cytochrome b6f complex. In the $C_2S_2M_2L_4$ PSII-LHCII supercomplex, four PLQ molecules were identified. The well-characterized Q_A and Q_B sites are asymmetrically arranged around the non-heme iron, with Q_A deeply buried within the core and Q_B positioned peripherally to permit PQL/PQLH2 exchange. A third site, Q_C, was described in an earlier a X-ray structure[56] but was absent in later studies[7]. In our structure reveals a weak, horizontally orientated density at the Q_C position, suggesting that Q_C may indeed be presence. Both Q_B and Q_C sites connect to an internal cavity (Extended Data Fig. 3e) lined with lipids. Two channels link this cavity to the thylakoid membrane: Channel I, containing Q_C's tail between cytochrome b_{559} α (PsbE) and PsbJ (Extended Data Fig. 3e, bottom left panel), and Channel II, containing the Q_B's tail between D_2 and cytochrome b_{559} β (PsbF), more stromal-exposed (Extended Data Fig. 3e, top right panel). The Q_B's headgroup neighbors the non-heme iron and forms hydrogen bonds with residues Ser264 and Phe265 on the D1 subunit, whereas the Q_C's headgroup remains unbound.

A transmembrane density was identified within the cleft among CP24, CP29, and the core, a region previously proposed as the localization site for PsbS, a protein essential for NPQ[29]. This density, absent from all previously reported PSII cryo-EM structures, extends from below the lumenal β-sheet loop of CP29 to the stromal side, displaying well-defined structural features that lack characteristics typical of protein side chains. The overall shape and properties of the density closely match those of the lipid-like

molecule PL9, which we therefore tentatively modeled at this position (Extended Data Fig. 3e,f, bottom right panels). These observations confidently exclude the possibility of PsbS localization in this region.

Supplementary Methods

Cryo-EM data preprocessing, particle selection and sorting strategy

As outlined in the main text, this process involved successive rounds of particle picking, 2D classification, and 3D analysis—including initial reconstruction, 3D classification, and multi-level local refinement. As map quality improved, we utilized Gautomatch and cryoSPARC for template matching, which progressively enhanced the resolution of 3D reconstructions. To maximize the yield of high-quality particles, we merged datasets from classes displaying clear structural features of PSI-LHCI or PSII-LHCII for subsequent focused 3D classification. Further procedural details are provided in the sections below.

Initial 3D Reconstruction of PSII-LHCII

For the PSII-LHCII supercomplex, which is predominantly localized within the tightly appressed, planar regions of the grana. In our chloroplast samples, this rigid, parallel membrane architecture imposes a significant constraint on the Euler angle distribution, as particles are predominantly "locking" in a fixed orientation relative to the membrane normal. Furthermore, during cryo-EM grid preparation, chloroplasts are likely constrained by the thin aqueous layer within the grid holes, causing the grana stacks to preferentially orient parallel to the grid surface. These would cause the grana stacks to

preferentially orient parallel to the grid surface, leading to sampling bias and the observed anisotropy in the final reconstruction. For the peripheral antenna regions of PSII-LHCII, particularly the L-LHCII trimers, substantial flexibility was observed, along with weak but persistent density in our reconstructions using an 800-pixel box size. To address the resulting heterogeneity, mitigate the effects of preferred orientation, and improve map quality, we performed extensive 3D classification on the initially acquired data to enrich homogeneous particle populations. Additionally, we applied the "Rebalance Orientations" tool in cryoSPARC to achieve a more isotropic sampling of the final map and reduce anisotropy in the reconstruction.

Cross-classification of multiple supercomplexes

After multiple rounds of cross-classification and local refinement, we identified several distinct supercomplexes, including not only PSI-LHCI and $C_2S_2M_2L_4$ PSII-LHCII, but also three types of PSII-LHCII dimer (SS, TL and TS), one type of PSII-LHCII trimer (TL-TS), two types of PSII-LHCII tetramer (TL-TS-SS, TL-TS-TL) and one type of PSI-LHCI-LHCII. To validate these structural forms, we conducted reference-free 2D classification following the 3D processing steps. This approach allowed direct visualization of characteristic features from 2D averages without introducing reference bias.

To enhance local resolution and generate composite maps suitable for model building, we applied a hierarchical masking strategy for the local refinement of all PSI and PSII types, as previously described[28].

To minimize the possibility that the particles used for the final reconstructions of higher-order PSII assemblies (dimers, trimers and tetramers) were derived from smaller or incompletely occupied assemblies, we performed independent 3D classification on each constituent of the assemblies. For each assembly type, soft masks were generated around individual PSII positions and 3D classification without alignment was performed to assess the occupancy and conformational state of each position independently. This consistently yielded a major complete class with improved resolution compared to the initial unclassified consensus map, suggesting enrichment for a homogeneous population. To further ensure compositional integrity, we used metadata intersection (particle UIDs) to select only those particles that belonged to the high-resolution, structurally complete classes for every constituent simultaneously. The occupancy analysis results are presented in Supplementary Figs. 2j,k, 3h,i, 4h,i,s, 5f,j,m, 6h-j.

Quantification of structural heterogeneity of PSII dimers

To quantify the conformational heterogeneity of individual PSII monomers within PSII dimeric assemblies, we compared the particle orientations obtained from focused refinement (individual PSII monomer) and consensus refinement (whole PSII dimer). First, a consensus local refinement was performed using a mask covering the entire dimer, with the alignment resolution limited to 4.5 Å. This strategy prevents the alignment from being dominated by one side that achieves high resolution first; by limiting the resolution, we ensured a balanced global alignment for both sides of the dimer based on the overall assembly architecture. Subsequently, independent focused

refinements were performed for each constituent PSII monomer using localized masks to capture their specific, high-resolution orientations.

For each PSII dimer type (SS, TL, TS) and each monomer position, the angular and translational shifts were calculated per particle. Particles were matched by their unique identifiers (UIDs) to ensure that the same physical particle from the consensus refinement was directly compared with its counterpart from the focused refinement. Angular shifts were computed as the geodesic distance between the rotation vectors (stored as alignments3D/pose in cryoSPARC particle files) using the scipy.spatial.transform.Rotation module. Translational shifts were calculated as the Euclidean distance between translation vectors (stored as alignments3D/shift), converted from pixels to angstroms using the corresponding pixel size for each reconstruction. All codes used to quantify structural heterogeneity of PSII dimers (analyze_refine.py and calcu_angles.py) are available at https://github.com/JackZhang-Lab/EM-scripts.

Model building, refinement, and validation

Atomic models were constructed manually in Coot. Initially, high-resolution structures of *Pisum sativum* PSII (PDB 5XNL) and PSI (PDB 6YEZ) were rigid-body fitted into their corresponding density maps using ChimeraX (1.6.1). The fitted models were then subjected to manual mutation, adjustment, and real-space refinement in Coot to correct local errors and optimize the fit to the density. Final models were refined with phenix.real_space_refine under geometric restraints and validated using MolProbity. For the lower-resolution L-LHCII regions in the $C_2S_2M_2L_4$ model, we fitted the distinct

triangular densities with the trimeric LHCII models from the PSI-LHCI-LHCII complex, with side chains and ligands removed. For model building of the PSII-LHCII dimers, trimer and tetramers, we employed a rigid-body docking approach, individually fitting the high-resolution $C_2S_2M_2$ supercomplex model, with side chains and ligands removed, into the corresponding density maps.

Models and density maps were visualized using UCSF ChimeraX[57] and PyMOL (the PyMOL Molecular Graphics System, Schrödinger, LLC) for analysis and figure preparation. Videos were generated using UCSF ChimeraX and edited with Movavi Video Editor (26.17.0).

Energy transfer calculations

$C_2S_2M_2L_4$

To calculate the excitation energy transfer (EET) dynamics within the $C_2S_2M_2L_4$ particle and the side-by-side PSII dimer (2x $C_2S_2M_2L_4$) we have followed the methodology originally developed by Bennett et al.[33] and modified by Leonardo et al.[34]. In short, we have used semi-empirical Hamiltonians from literature[58-63] to describe the site energies and inter-pigment couplings for the chlorins within the different subunits of the $C_2S_2M_2L_4$ particle, and used the TrEsp method[64] to calculate inter-pigment couplings for chlorins belonging to different complexes, based on the resolved structure. Semi-empirical Hamiltonians for the minor complexes CP24 and CP26 are however still missing in literature. We have used the Hamiltonian of LHCII[61] for CP24, and took out Chl *b*605, Chl *a*613 and Chl *a*614, which are absent in this antenna complex. For

CP26, the LHCII semi-empirical Hamiltonian without Chl *b*605 was used. In CP26 Chl 609 is however a Chl *a* instead of Chl *b*. To accommodate for this difference the site energy of Chl 609 was set at the site energy of the Chl *a*609 in CP29 (15,069 cm^{-1})[63]. To be consistent with the methodologies that were employed to construct the LHCII semi-empirical Hamiltonian[61], the electronic couplings to Chl *a*609 and the rest of the Chls in CP26 were calculated using the dipole-dipole approximation using transition dipole moment magnitudes (TDMs) for Chl *a* and *b* as established in Novoderezhkin et al. (2011) (Supplementary Table 7). Finally, Chl *a*616 of CP29 is not described in the published semi-empirical Hamiltonian[63]. The site energy of this Chl was set at 14,971 cm^{-1}, as structure-based charge-density coupling calculations have estimated its energy 50 cm^{-1} below that of Chl *a*602[65] (which is at 15,021 cm^{-1} in [63]). Couplings to this Chl were again computed with the dipole-dipole approximation with Chl *a* and *b* effective TDMs consistent with [63] (Supplementary Table 7).

The atomic transition charges used for calculating the TrEsp electronic couplings were retrieved from [59,64,66] and scaled to match the TDMs that were determined for the different chlorins from different subunits in literature (Supplementary Table 7).

The spectral properties of the pigments were constructed using two different forms of spectral densities (SD). The SD types a and b (Supplementary Table 8) belong to the first SD form ($\chi_1''(\omega)$) and are described by equation 1. The SD types b, c, d, e and f belong to the second SD form ($\chi_2''(\omega)$) and are described by equation 2. Supplementary Table 7 describes which pigments are described with which SD type and Supplementary Table 8 lists the parameters used for each SD type.

$$\chi_1''(\omega) = (\pi\hbar)\frac{S_0}{s_1+s_2}\sum_{i=1,2}\frac{s_i(\omega^5)}{7!2\omega_i^4}e^{-\sqrt{\frac{\omega}{\omega_i}}} \quad (1)$$

$$\chi_2''(\omega) = 2\lambda_0\frac{\omega\gamma_0}{\omega^2+\gamma_0^2} + \sum_{j=1}^{N_{vib}} 2S_j\omega_j^3\frac{\omega\gamma_{vib}}{(\omega_j^2-\omega^2)^2+\omega^2\gamma_{vib}^2} \quad (2)$$

After the construction of the Hamiltonian of the $C_2S_2M_2L_4$ particle we have generated 500 realizations of disorder in pigment site energies with inhomogeneous broadening widths σ_{inhom} as described in Supplementary Table 7 Based on these realizations strongly excitonically interacting pigments were grouped into domains based on their excitonic overlap, following the procedure that is presented in [33]. Excitation energy transfer rates between excitons belonging to the same domain are calculated according to modified Redfield theory, and rates of energy transfer between excitons belonging to different domains are computed using generalized Förster theory, considering a temperature of 300 K. The computation of the excitation energy transfer rates using these procedures was performed with the Matlab script of Yang et al. (2024)[54], which is available at https://zenodo.org/records/13346121.

Equipped with the resulting average energy transfer rate matrix we could calculate the excitation energy trapping dynamics of the $C_2S_2M_2L_4$ particle and the side-by-side PSII dimer. To do so, we added a photochemical trap channel to the lowest exciton states of the RCs, with an infinite rate toward the trap and without back transfer[34,54,67]. In addition, we added a natural decay rate of 0.5 ns^{-1} to each exciton in the complex[68]. Then, we calculated the time-dependent population in the trap channel, starting from the condition in which all excitons are evenly excited. From this curve we could ultimately determine the excitation lifetime of the system.

$C_2S_2M_2$ - $C_2S_2M_2L_4$ Intermediates

The trapping kinetics for the $C_2S_2M_2$ particle were calculated from the $C_2S_2M_2L_4$ rate matrix excluding the L_4 part. This approach is possible because of the relative isolation of the L_4 LHCII trimers from each other and from the rest of the PSII supercomplex, which causes there to be no exciton delocalization over from L_4 to $C_2S_2M_2$. Similarly, to simulate the decay curves of PSII particles with partial occupancies of the L-LHCII trimers, the missing L-LHCII trimers were simply excluded from the energy transfer matrix.

To simulate the fluorescence decay kinetics for PSII ensembles with a fractional average number of n LHCII trimers per PSII, we modelled the ensemble as a stochastic distribution of discrete particles. Assuming that the S and M trimers are constitutively bound, the observed variability in antenna size was attributed to the independent binding of L-LHCII trimers at the four potential sites per PSII dimer. The probability P(k) of a complex containing exactly k L-LHCII trimers was determined using a binomial distribution:

$$P(k) = \binom{4}{k} p^k (1-p)^{4-k} \qquad (3)$$

where p is the occupancy probability of a single L binding site, defined by the average number of L-LHCII trimers per monomer (p = (n-2)/2). The final ensemble-averaged decay curve for a given n was calculated as the linear superposition of the simulated decays for all 16 L-LHCII trimer binding configurations, each weighted by its specific probability.

LHCII - PsaO

The rate of excitation energy transfer between the LHCII Chl *a* 610-611-612 exciton and the PsaO Chl *a*201, and that between the Chl *a* 613-614 LHCII exciton and the PsaO Chl *a*204 were computed using the parameters for Chl *a* in LHCII as described above (Supplementary Tables 7 and 8). The total energy transfer rate from exciton domain M to a single Chl n is the sum of each individual exciton energy transfer rate multiplied by its Boltzmann population within the domain[60]:

$$k_{M\to n} = \sum_{m\in M} k_{m\to n} P_m^{Boltz} \qquad (4)$$

$$P_m^{Boltz} = \frac{e^{-\frac{E_m}{k_B T}}}{\sum_{m\in M} e^{-\frac{E_m}{k_B T}}} \qquad (5)$$

With E_m the energy of exciton m.

PSI-LHCI

Excitation energy transfer rates between Chls in the LHCI belt and the PSI core have been calculated using the FRET framework as presented in Croce & Amerongen (2020)[69]. In this framework the excitation energy transfer rate from chlorin m to n, $k_{m\to n}$ (in ps^{-1}), is given by:

$$k_{m\to n} = C_{DA} \frac{\kappa^2}{R^6} \qquad (6)$$

With C_{DA} a chlorin-type dependent constant, R the center-to-center distance in nm and the orientation factor κ calculated as:

$$\kappa = \hat{\mu}_D \cdot \hat{\mu}_A - 3(\hat{\mu}_D \cdot \hat{R}_{DA})(\hat{\mu}_A \cdot \hat{R}_{DA}) \qquad (7)$$

With $\hat{\mu}_{D/A}$ the unit vector of the transition dipole moment of the donor (D)/acceptor (A), and $\hat{R}_{DA}$ the unit vector of the center-to-center vector. The chlorin transition dipole moment $\hat{\mu}$ is defined as the line connecting the pyrrole nitrogen atom of ring C to that

of ring A, and chlorin centers are defined as the geometric center of all four pyrrole nitrogens. The C_{DA} factor depends on the donor and acceptor chlorins and is 5.59 for Chl *a* → Chl *a* transfer, 0.19 for Chl *a* → Chl *b* transfer, 1.66 for Chl *b* → Chl *a* transfer and 2.50 for Chl *b* → Chl *b* transfer. The pheophytins were considered as Chls *a* with regards to the C_{DA} factor. The FRET rates have been calculated using Python with the algorithm that is available at https://zenodo.org/records/13283796.

LHCII thermalized depopulation rate calculation

Using the calculated average rate matrix, we could compute a total thermalized depopulation rate for each of the LHCII complexes (k_{out}): i.e., we determine per LHCII the Boltzmann equilibria of its excitons, and use these fractional equilibria P_i to weigh the sum of outgoing rates to the all of the excitons that are outside of the LHCII in question:

$$k_{out} = \sum_i^{N_{exc.\,\in LHCII}} P_i \sum_j^{M_{exc.\,\notin LHCII}} k_{i\to j} \qquad (8)$$

Supplementary Figures

Supplementary Fig. 1

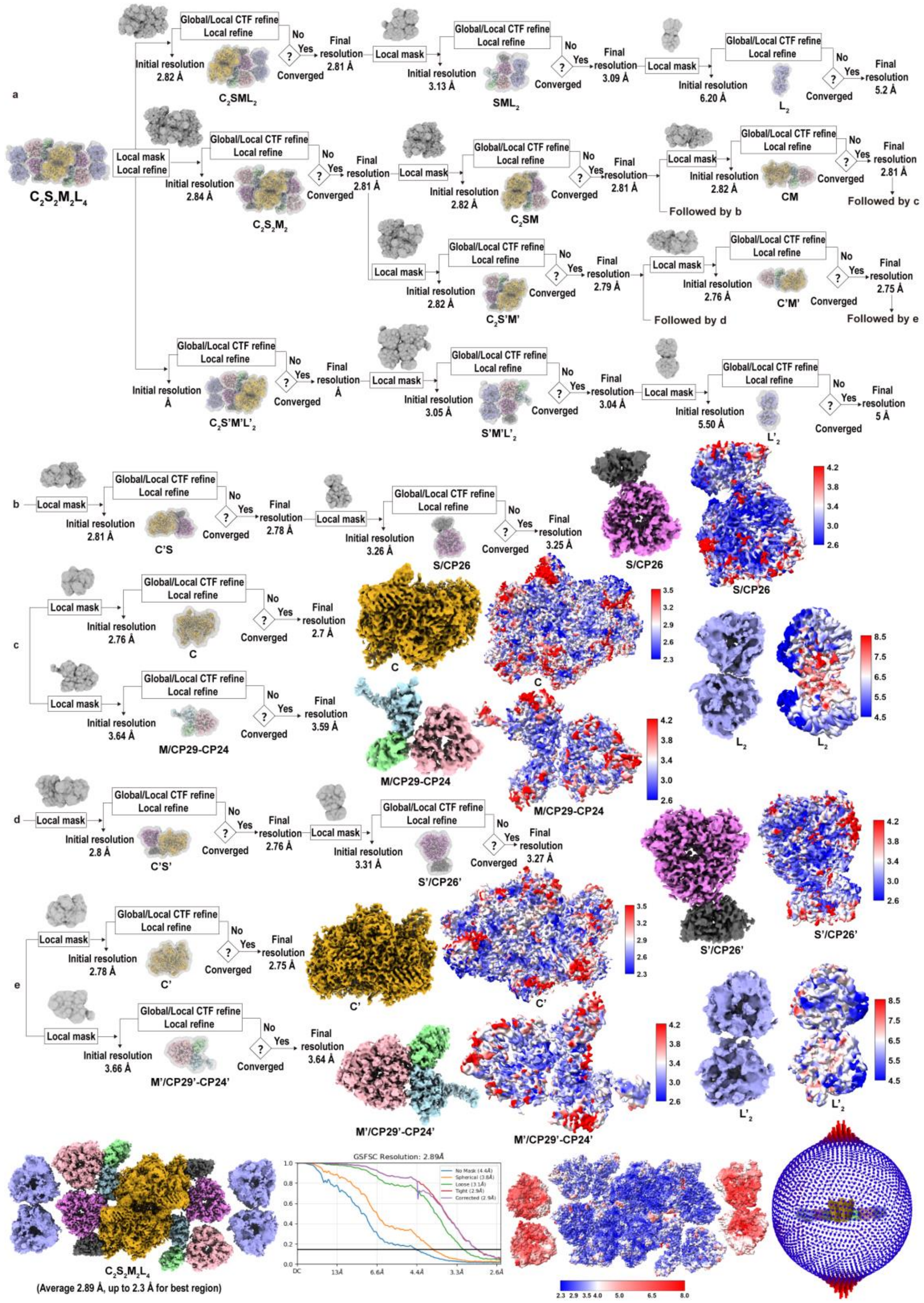

Supplementary Fig. 1 | Schematic workflow for multi-level refinement of the $C_2S_2M_2L_4$ PSII-LHCII supercomplex (EMD-68060).

a, Multi-level local refinement generated density maps of loosely bound LHCII trimers (L_2 and L'_2, shown as light slate blue), core with moderately bound LHCII trimer and CP29-CP24 (CM/CP29-CP24 and C'M'/CP29'-CP24', shown as golden amber, light pink, cyan and pale green respectively), with grey transparent surfaces indicating mask boundaries. The initial $C_2S_2M_2L_4$ PSII-LHCII map was obtained using the method described in Extended Data Fig. 1, with an average resolution of 2.89 Å (reach up to 2.3 Å). A hierarchical masking strategy was implemented following an overall-to-local and large-to-small scale progression for localized refinement of distinct regions. This was followed by sequential procedures including global CTF correction, local CTF refinement, and local refinement iteratively until converged. Final generated L_2, CM/ CP29-CP24, C_2SM/CP26-CP29-CP24, C'M'/CP29'-CP24', C_2S'M'/CP26'-CP29'-CP24' and L'_2 maps at resolutions of 5.2 Å, 2.81 Å, 2.75 Å, 2.79 Å and 5 Å, respectively. L_2 and L'_2 were locally refined using a single mask, whereas CM/ CP29-CP24, C_2SM/CP26-CP29-CP24, C'M'/CP29'-CP24', C_2S'M'/CP26'-CP29'-CP24' were divided into more local regions for further improvement. **b**, Multi-level local refinement of C_2SM/CP26-CP29-CP24, which generated maps in the region of S/CP26 at resolution of 3.25 Å. **c**, Multi-level local refinement of CM/CP29-CP24, which generated maps in the regions of core and M/CP29-CP24 at resolutions of 2.7 Å and 3.59 Å, respectively. **d**, Multi-level local refinement of C_2S'M'/CP26'-CP29'-CP24', which generated maps in the region of S'/CP26' at resolution of 3.27 Å. **e**, Multi-level local refinement of C'M'/CP29'-CP24', which generated maps in the regions of core and M'/CP29'-CP24' at resolutions of 2.75 Å and 3.64 Å, respectively. Gold standard

FSC curve from cryoSPARC. Orientation plot of particles included in the final 3D reconstruction.

Supplementary Fig. 2

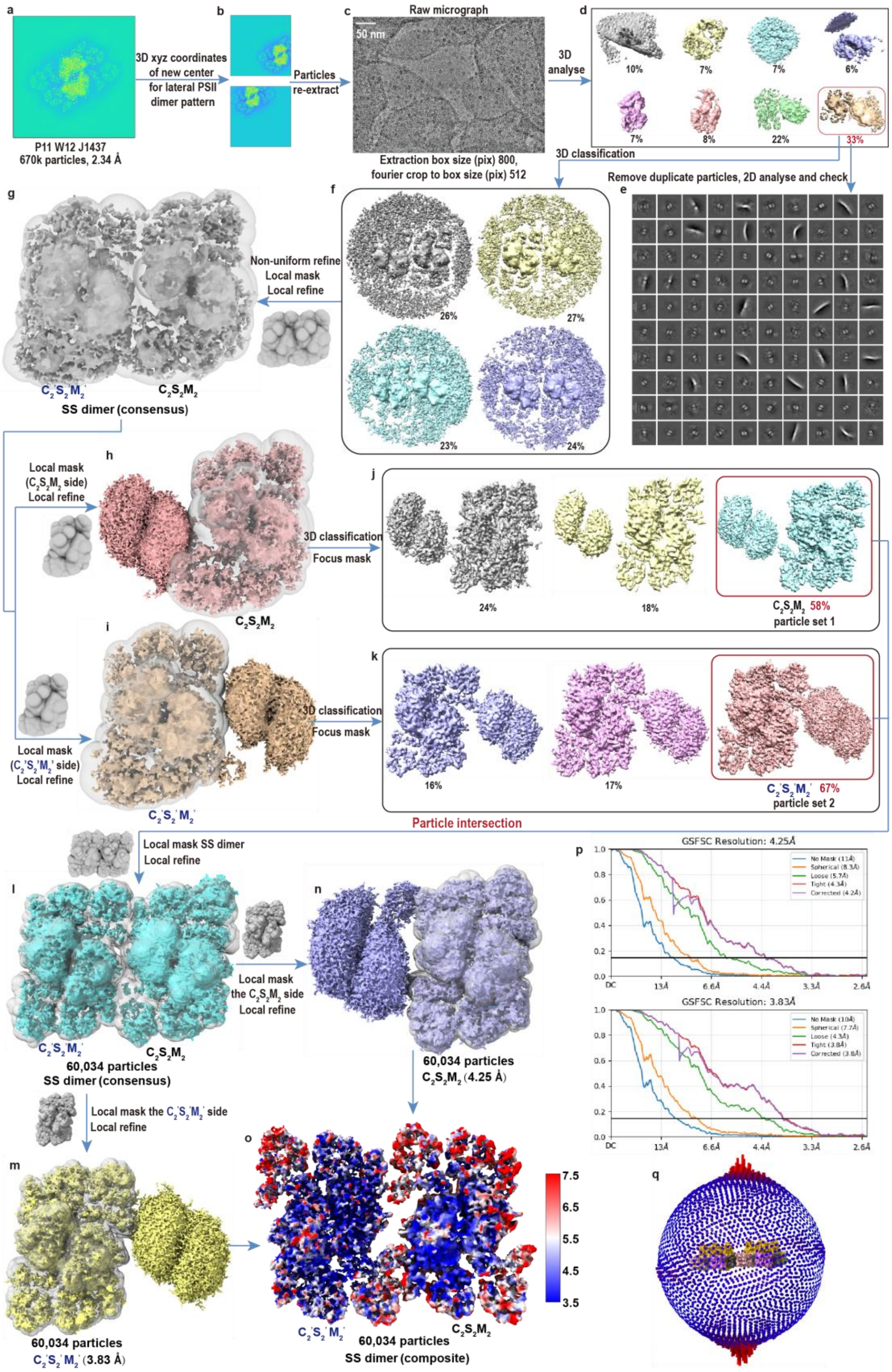

Supplementary Fig. 2 | Schematic workflow used to obtain the map of the SS dimer (EMD-81438).

In both the 2D and 3D maps of $C_2S_2M_2L_4$ PSII-LHCII reconstructed with an 800-pixel box, faint densities resembling an additional lateral PSII were observed. Therefore, subsequent processing was performed using the dataset reconstructed $C_2S_2M_2$, which offered the largest data volume. **a-c**, Based on the spatial location of these additional PSII weak features, a new 3D XYZ coordinate was defined to re-extract particle subset. **d**, Following 3D analysis, a well-defined lateral PSII-LHCII structural model was obtained. **e**, 2D classification was performed to further validate particle homogeneity within this side-by-side PSII-LHCII model, which also revealed structural characteristics consistent with lateral organization. **f**, 3D classification was performed to assess the structural heterogeneity of the sorted SS dimer population. **g**, Non-uniform refinement was then applied, followed by local refinement using a mask focused on the lateral dimer. **h**,**i**, Further density improvement was achieved through local masking of individual PSII units, respectively. When the focus is placed on one PSII-LHCII supercomplex, the other appears blurred. **j**,**k**, 3D classification of each PSII unit yielded particles selected for high quality and enriched for complete populations. **l**, The SS dimer consensus map with balanced sides. Built from the intersection of best particles from each side. Consensus local refinement used a full-dimer mask with alignment resolution limited to 4.5 Å, yielding balanced alignment for both PSII units. **m**,**n**, Focused refinements of individual PSII units. **o**, Local resolution map of the SS dimer (composite). The composite map was generated by aligning the two independently refined submaps multiplied with the mask to remove densities outside the masked region (panels m and n) in the lateral plane based on the overall map (panel l) using the

‘fit in map’ function in UCSF ChimeraX. The alignment was based on their cross-correlated density in the overlapping region. After verification of the precise alignment, the two maps were merged by compositing them into a single map using the ‘vop maximum’ command in UCSF ChimeraX, which takes the maximum density value at each grid point from the two input maps. **p**, Gold standard FSC curves from cryoSPARC. **q**, Orientation plot of particles included in the final 3D reconstruction.

Supplementary Fig. 3

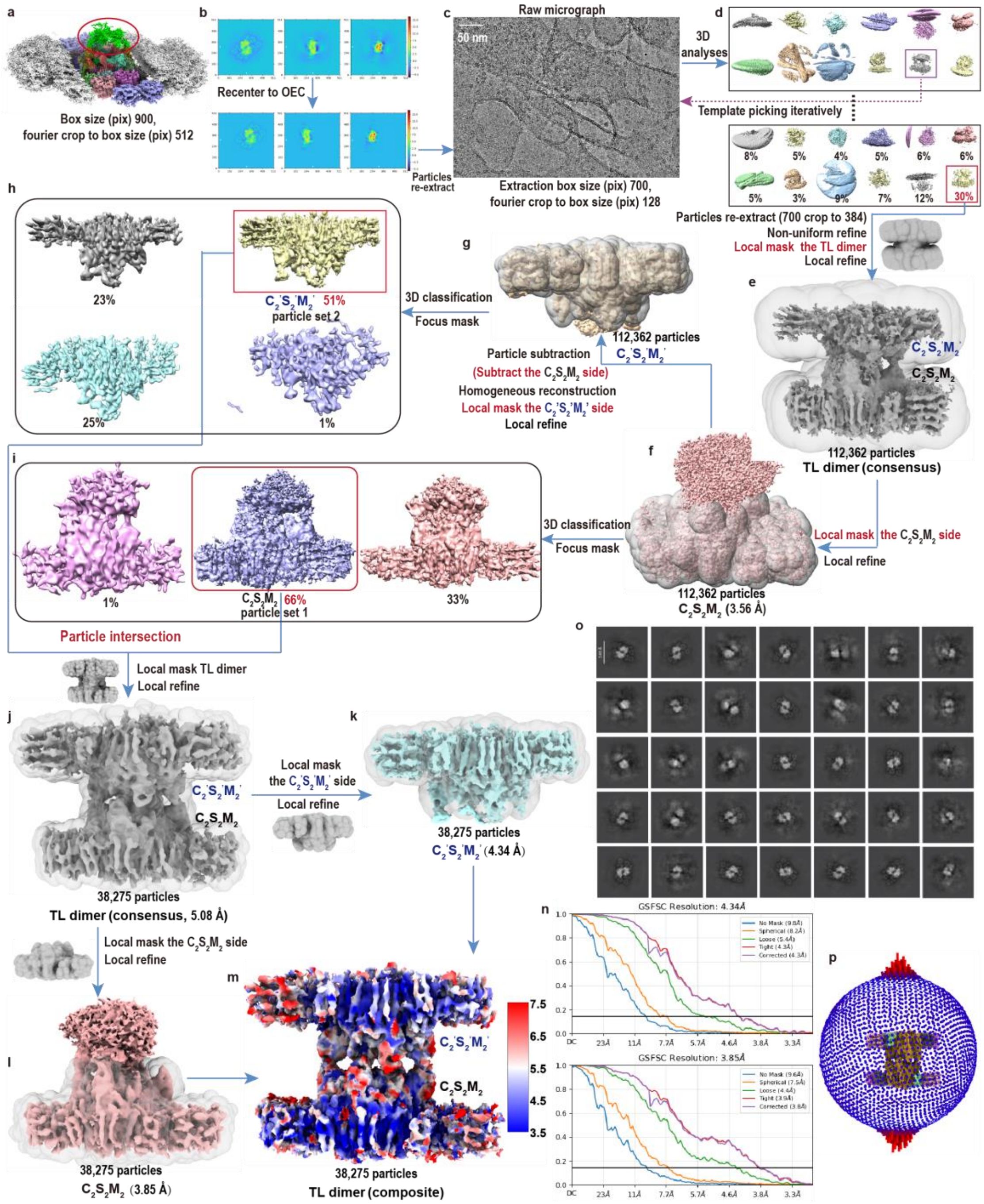

Supplementary Fig. 3 | Schematic workflow used to obtain the map of the TL dimer (EMD-81447).

The TL dimer was refined following a procedure analogous to that employed for the SS dimer. **a**, 3D reconstruction of $C_2S_2M_2L_4$ from an 800-pixel box revealed faint lumenal-side densities resembling an additional PSII. Subsequent processing was

performed using the dataset reconstructed $C_2S_2M_2$, which offered the largest data volume. **b,c**, A new particle subset was re-extracted using 3D coordinates centered on the OEC, based on the location of the extra lumenal-side feature. **d**, Following 3D analysis, a well-defined TL dimer model was obtained. Due to preferential signal alignment toward one side, density was stronger on one side and weaker on the other. **e**, Local refinement was performed using a mask focused on the TL dimer. **f**, Local refinement was applied using a mask on the strong side of the dimer. **g**, Signal from the strong side was computationally subtracted using cryoSPARC, followed by local refinement targeting the weak side. **h**,**i**, 3D classification of each PSII unit yielded particles selected for high quality and enriched for complete populations. **j**, The TL dimer consensus map with balanced sides. Built from the intersection of best particles from each side. It was generated using the same method as described in Supplementary Fig. 2l. **k**,**l**, Focused refinements of individual PSII units. **m**, Local resolution map of the TL dimer (composite). The composite map was generated using the same method as described in Supplementary Fig. 2o. Briefly, it was generated by compositing the independently refined submaps from panels k and l based on their alignment to the overall map (panel j). **n**, Gold standard FSC curves from cryoSPARC. **o**, 2D classification revealed structural features consistent with trans-stromal organization. **p**, Orientation plot of particles included in the final 3D reconstruction.

Supplementary Fig. 4

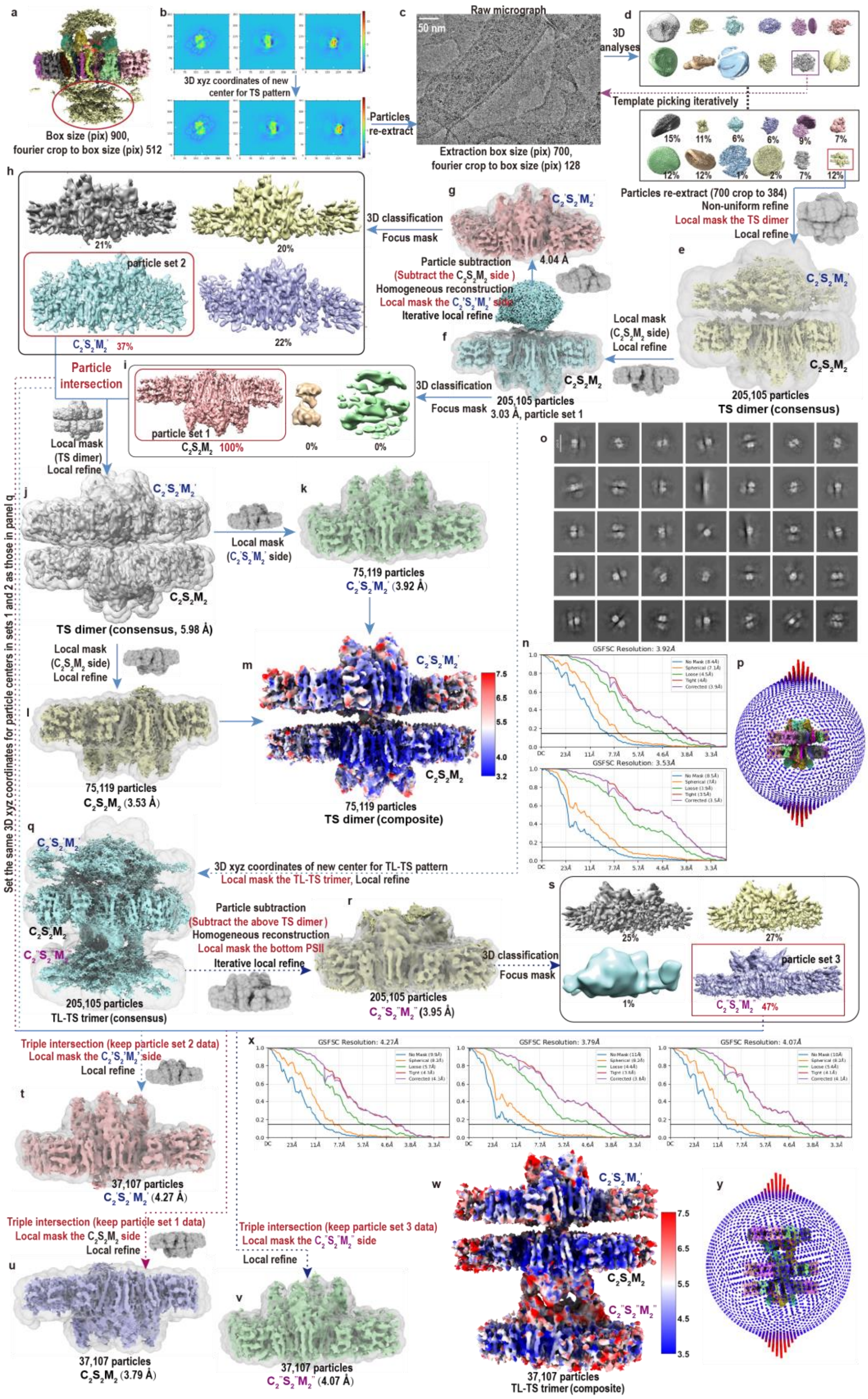

Supplementary Fig. 4 | Schematic workflow used to obtain maps of the TS dimer and the TS-TL trimer (EMD-68049).

The TS dimer and the TS-TL trimer were refined following a procedure analogous to that employed for the SS and TL dimers. **a**, 3D reconstruction of $C_2S_2M_2L_4$ from an 800-pixel box also revealed faint stromal-side densities resembling an additional PSII. Subsequent processing was performed using the dataset reconstructed $C_2S_2M_2$, which offered the largest data volume. **b, c**, A new particle subset was re-extracted with a box size of 700 pixels and subsequently down-scaled to 128 pixels by shifting the center of 3D coordinates to a position between PSII and the additional density feature on the stromal side. **d**, Following iterative 3D analysis, a well-defined structural model of the TS dimer was obtained. Due to preferential signal alignment toward one side, density was stronger on one side and weaker on the other. **e**, Particles corresponding to the well-refined trans-stromal class were re-extracted. Subsequent refinements included a non-uniform refinement and a local refinement employing a mask focused on the TS dimer. **f**, Local refinement using a mask on the strong side ($C_2S_2M_2$) of the dimer. The particle stack used for refinement was designated as set 1. **g**, Signal of the strong side was subtracted by applying a static mask in cryoSPARC, followed by homogeneous reconstruction and iterative local refinements targeting the weak side (C_2'S_2'M_2'). **h**, **i**, 3D classification of each PSII unit yielded high-quality particles enriched for complete populations. For the weak side (C_2'S_2'M_2'), a particle subset was obtained and designated set 2. **j**, The TS dimer consensus map with balanced sides. Built from the intersection of best particles from each side. It was generated using the same method as described in Supplementary Fig. 21l. **k**,**l**, Focused refinements of individual PSII units. **m**, Local resolution map of the TS dimer (composite). The composite map was

generated using the same method as described in Supplementary Fig. 2o. Briefly, it was reconstructed by compositing the independently refined submaps from panels k and l base on their alignment to the overall map (panel j). **n**, Gold standard FSC curves from cryoSPARC. **o**, 2D classification revealed structural features consistent with trans-stromal organization. **p**, Orientation plot of particles included in the final 3D reconstruction (n) for the TS dimer. **q**, To resolve higher-order structural features across both the stromal and lumenal sides, the coordinate origin of particles was repositioned to the center of the trimer. Subsequently, a local refinement was performed targeting the TL-TS trimer using a mask designed from the binding patterns of the previously resolved TL and TS dimers. **r**, The corresponding TS dimer volume, defined above, was aligned to this new origin, and its signal was subtracted from the particle stack using a static mask in cryoSPARC. This was followed by homogeneous reconstruction and iterative local refinements focused on the weak side of TL region (C_2''S_2''M_2''). **s**, 3D classification of the weak side of TL region (C_2''S_2''M_2'') yielded a particle subset (designated set 3) with improved density features within the corresponding region. **t-v,** Particles from panels h (set 2) and i (set 1) were realigned to the same origin established in panel q. Common particles intersecting across the recentered sets 1, 2, and set 3 were selected from their respective parent sets. Focused local refinement was performed separately on each resulting particle subset. **w**, Local resolution map of the TL-TS trimer (composite). The composite map was generated using the same method as described in Supplementary Fig. 2o. Briefly, it was generated by compositing the focused-refined submaps from panels t, u and v based on their alignment to the overall

map (panel q). **x**, Gold standard FSC curves from cryoSPARC. **y**, Orientation plot of particles included in the final 3D reconstruction.

Supplementary Fig. 5

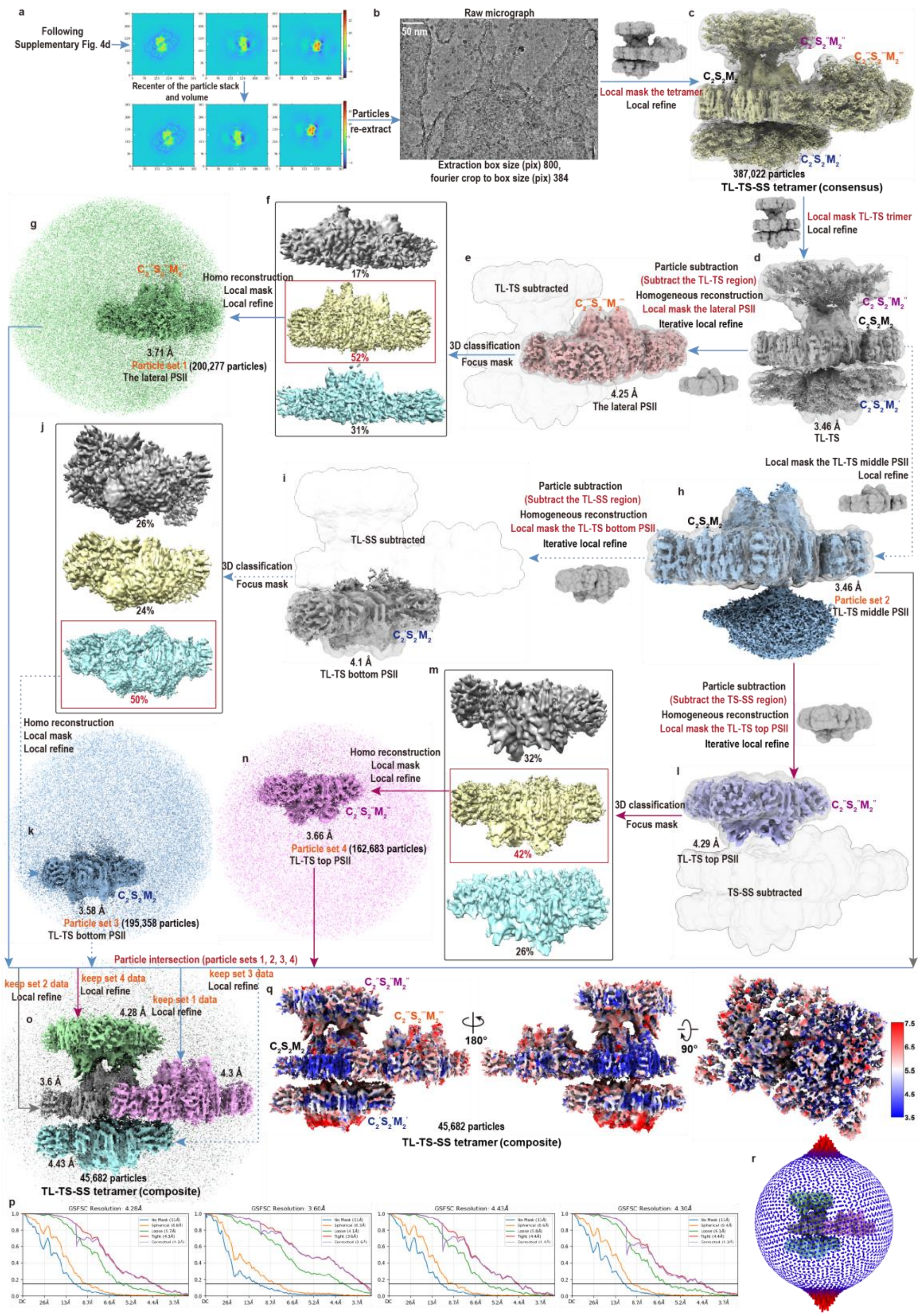

Supplementary Fig. 5 | Schematic workflow used to obtain the TL-TS-SS tetramer (EMD-68047).

a, Following Supplementary Fig. 4d, particles representing the high-quality TL dimer conformation were pooled and deduplicated. The coordinate origin origin for both the particle stack and the reconstructed volume was translated to a position proximal to the edge of the central PSII within the TL-TS trimer. **b**, The particle stack was re-extracted with a box size of 800 pixels and subsequently down-scaled to 384 pixels. **c**, Local refinement was performed using a mask focused on the TL-TS-SS tetramer, which was designed based on the binding patterns derived from the previously resolved structures of the SS, TL, and TS dimers. **d**, Local refinement employing a mask focused on the TL-TS trimer to optimize signal alignment within this region. **e**, Signal of the TL-TS trimer region was subtracted by applying a static mask in cryoSPARC, followed by homogeneous reconstruction and iterative local refinements targeting the attached lateral PSII (C_2'''S_2'''M_2'''). The position of the mask used for the TL-TS trimer is indicated by a translucent cartoon. **f,** 3D classification of the attached lateral PSII yielded a particle subset with improved density features within the corresponding lateral region. **g**, Local refinement further improved the density for the attached lateral PSII (C_2'''S_2'''M_2'''), yielding a map at 3.71 Å resolution. The particle subset used for this reconstruction is designated set 1. The noise within the box were retained to illustrate the relative spatial position of the attached lateral PSII. **h**, Following panel d, local refinement was performed by applying a mask focused on the middle PSII ($C_2S_2M_2$) within the TL-TS trimer. The particle stack subjected to this refinement is designated as set 2. **i**, Signal of the TL-SS region was subtracted by applying a static mask, followed by homogeneous reconstruction and iterative local refinements targeting the

bottom PSII (C_2'S_2'M_2') of the TL-TS trimer. The position of the mask used for the TL-SS trimer is indicated by a translucent cartoon. **j**, 3D classification of the bottom PSII (C_2'S_2'M_2') of TL-TS trimer yielded a particle subset with improved density features within the corresponding bottom region. **k**, Local refinement further improved the density for the bottom PSII (C_2'S_2'M_2') of TL-TS trimer, yielding a map at 3.58 Å resolution. The particle subset used for this reconstruction is designated set 3. **l**, Signal of the TS-SS region was subtracted by applying a static mask, followed by homogeneous reconstruction and iterative local refinements targeting the top PSII (C_2''S_2''M_2'') of the TL-TS trimer. The position of the mask used for the TS-SS trimer is indicated by a translucent cartoon. **m**, 3D classification of the top PSII (C_2''S_2''M_2'') of the TL-TS trimer yielded a particle subset with improved density features within the corresponding top region. **n**, Local refinement further improved the density for the top PSII (C_2''S_2''M_2'') of the TL-TS trimer, yielding a map at 3.66 Å resolution. The particle subset used for this reconstruction is designated set 4. **o**, Common particles intersecting across particle set 1, 2, 3, and 4 were selected from their respective parent sets. Focused local refinement was performed separately on each resulting particle subset. The composite map of the TL-TS-SS tetramer was generated using the same method as described in Supplementary Fig. 2o. Briefly, it was reconstructed by compositing the focused-refined submaps based on their alignment to the overall map (panel c). The local resolutions of the different PSII units within this composite structure are 4.28 Å, 3.6 Å, 4.43 Å and 4.3 Å resolution, respectively. **p**, Gold standard

FSC curves from cryoSPARC. **q**, Local resolution maps. **r**, Orientation plot of particles included in the final 3D reconstruction for the TL-TS-SS tetramer (composite).

Supplementary Fig. 6

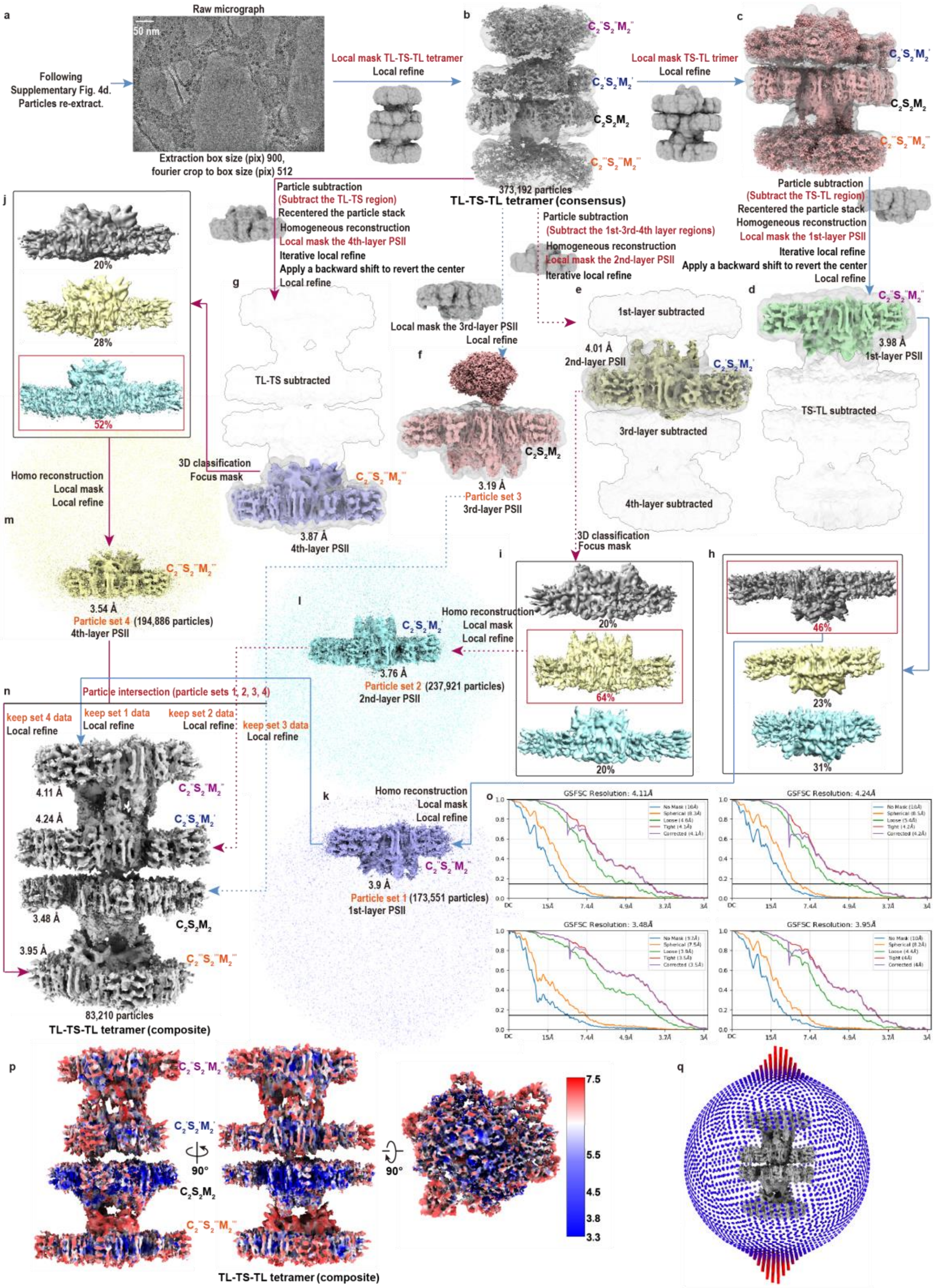

Supplementary Fig. 6 | Schematic workflow used to obtain the TL-TS-TL tetramer (EMD-68043).

The TL-TS-TL tetramer was refined following a procedure analogous to that used for the TL-TS-SS tetramer. **a**, Following Supplementary Fig. 4d, particles representing the high-quality TL dimer conformation were pooled, deduplicated, re-extracted with a box size of 900 pixels and subsequently down-scaled to 512 pixels. **b**, Local refinement was performed using a mask focused on the TL-TS-TL tetramer, which was designed based on the binding patterns derived from the previously resolved structures of the TL and TS dimers. **c**, Local refinement employing a mask focused on the TS-TL trimer region to optimize signal alignment. **d**, Signal of the TS-TL region was subtracted by applying a static mask in cryoSPARC, followed by recentering of the particle stack, homogeneous reconstruction and iterative local refinements targeting the first-layer PSII. A backward shift was then applied to revert the center of the particle stack and volume, followed by local refinement, yielding a map at 3.98 Å resolution for the first-layer PSII (C_2''S_2''M_2''). The position of the mask used for subtraction of the TS-TL trimer is indicated by a translucent cartoon. **e**, Signal of the first, third and fourth layers was subtracted by applying a static mask, followed by homogeneous reconstruction and iterative local refinements targeting the second-layer PSII (C_2'S_2'M_2'), yielding a map at 4.01 Å resolution for the second-layer PSII. The position of the mask used for subtraction of the first, third and fourth layers PSIIs is indicated by a translucent cartoon. **f,** Local refinement further improved the density for the third-layer PSII ($C_2S_2M_2$), which exhibited the strongest initial density, yielding a map at 3.19 Å resolution. The particle subset used for this reconstruction is designated set 3. **g**, The peripheral 4th-layer PSII (C_2'''S_2'''M_2''') was refined following a procedure analogous to that

employed for the first-layer PSII, yielding a map at 3.87 Å resolution for the 4th layer PSII. **h-j**, 3D classification of the PSII within the first (h), second (i), and fourth (j) layers of the TL-TS-TL tetramer separately yielded particle subsets with improved density features in these corresponding regions. **k-m**, Local refinement further improved the density for the first (k), second (l), and fourth (m) layer PSIIs, yielding reconstructions at 3.9 Å, 3.76 Å, and 3.54 Å, respectively. These particle subsets are designated as set 1, set 2, and set 4. **n**, Common particles intersecting across particle set 1, 2, 3, and 4 were selected from their respective parent sets. Focused local refinement was performed separately on each resulting particle subset. The composite map was generated using the same method as described in Supplementary Fig. 2o. Briefly, it was reconstructed by compositing the focused-refined submaps based on their alignment to the overall map (panel b). The local resolutions of the different PSII units within this composite structure are 4.11 Å, 4.24 Å, 3.48 Å and 3.95 Å, respectively. **o**, Gold standard FSC curves from cryoSPARC. **p**, local resolution maps. **q**, Orientation plot of particles included in the final 3D reconstruction for the composite TL-TS-TL tetramer.

Supplementary Fig. 7

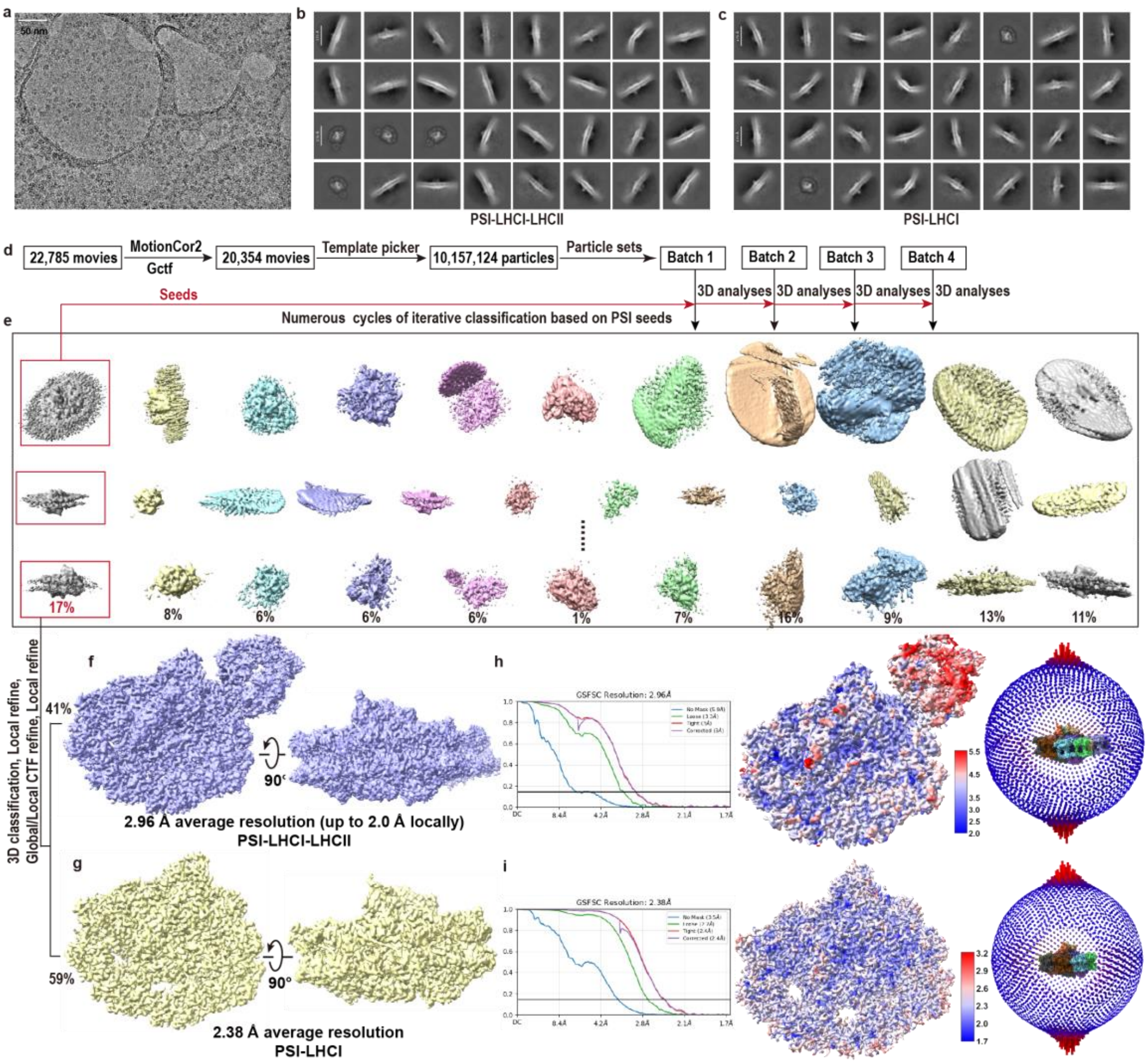

Supplementary Fig. 7 | Schematic workflow used to obtain the maps of the PSI-LHCI-LHCII and PSI-LHCI supercomplexes.

a, Representative cryo-EM micrograph of chloroplasts for single-particle analysis. Scale bar: 50 nm. **b**, **c**, Representative 2D class averages showing the two types of PSI supercomplexes, following 3D classification, are the PSI-LHCI-LHCII (b) and PSI-LHCI (c) supercomplexes, respectively. **d**, Based on the PSI-LHCI model derived from the processing results in Extended Data Fig. 1, further analyses were carried out. Using the PSI-LHCI model as a template, particles were re-extracted from micrographs and divided into four subsets. Each subset underwent independent 3D analysis. Particles

corresponding to well-defined PSI from the first subset were selected as seeds for multiple rounds of iterative 3D analysis applied to all four groups. High-quality particles identified across all groups were subsequently pooled and subjected to an additional round of 3D analysis. **e**, Through 3D classification, two distinct structural classes of PSI were resolved. Each class was individually refined using local refinement, followed by global CTF correction, local CTF refinement, and local refinement iteratively until converged. **f**, **g**, Final generated PSI-LHCI-LHCII (EMD-66932) and PSI-LHCI (EMD-66925) maps at average resolutions of 2.96 Å and 2.38 Å, respectively. **h**,**i**, Gold standard FSC curves from cryoSPARC, local resolution maps, and orientation plot of particles included in the final 3D reconstructions of in situ PSI-LHCI-LHCII (h) and PSI-LHCI (i), respectively.

Supplementary Fig. 8

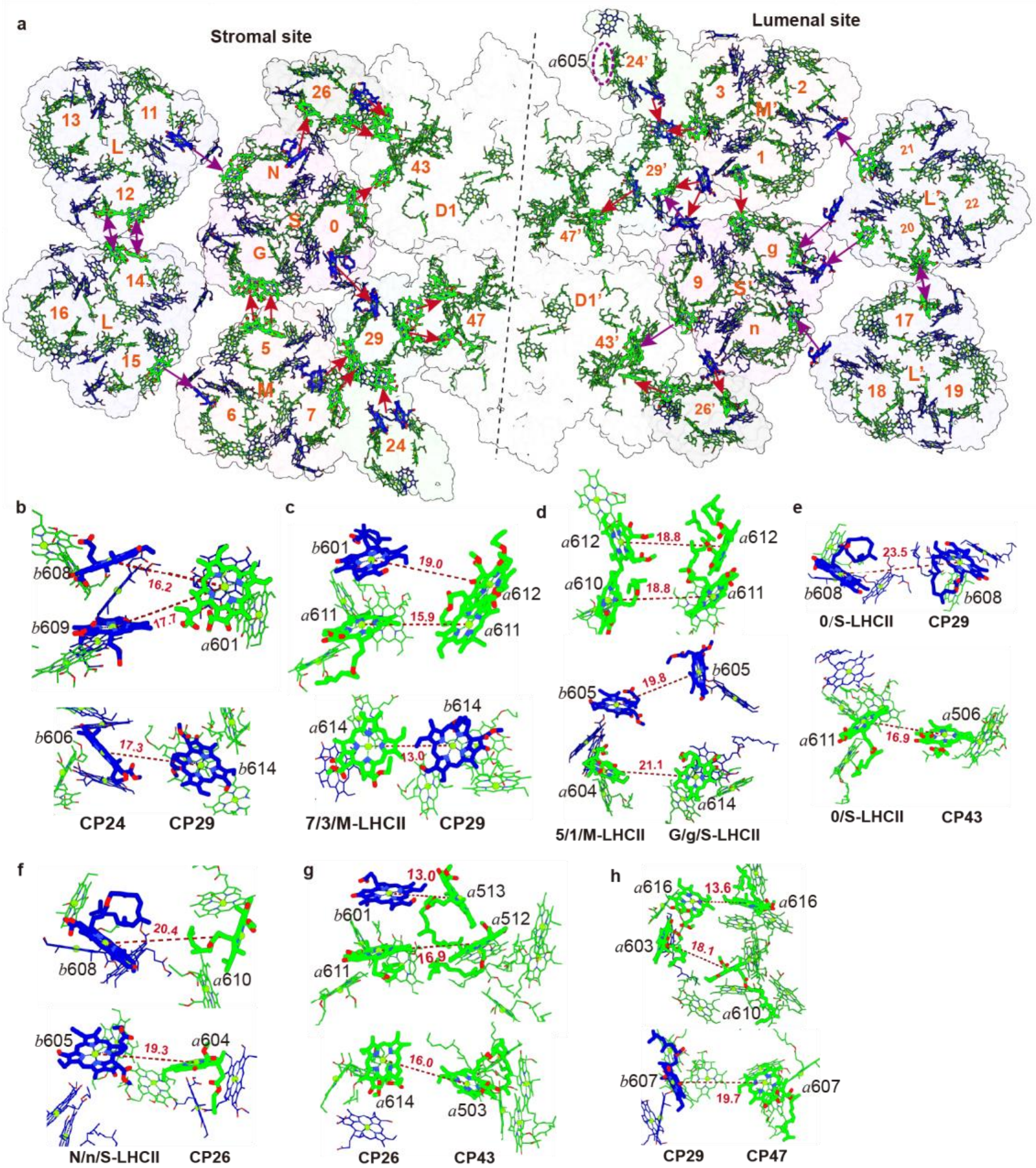

Supplementary Fig. 8 | Pigment organization and energy transfer pathways in the $C_2S_2M_2L_4$ type PSII–LHCII supercomplex.

a, Spatial distribution of chlorophylls across stromal and lumenal sides. The L-LHCII regions were fitted with the LHCII models from the PSI-LHCI-LHCII complex according to their distinct triangular densities. Chlorophylls proposed to mediate inter-subunit energy transfer are highlighted, with those on the stromal side shown to the left of the central black dashed line and their lumenal-side counterparts shown to the right.

Core and peripheral subunits are labeled according to their corresponding chain IDs in the deposited $C_2S_2M_2L_4$ PDB (PDB 21WV) model. Proposed energy transfer pathways from the outermost L-LHCII to adjacent complexes are indicated by red arrows, while other routes are marked by magenta arrows. The location of an additional Chl *a* molecule (Extended Data Fig. 3f), located at CP24 proximal to the core region, is annotated with a medium violet red dashed ellipse. **b–h,** Close-up views of proposed energy transfer pathways. Panels illustrate potential routes: (b) CP24 to CP29; (c) M-LHCII (chains 7 and 3) to CP29; (d) M-LHCII (chains 5 and 1) to S-LHCII (chains G and g); (e) S-LHCII (chain 0) to both CP29 and CP43; (f) S-LHCII (chains N and n) to CP26; (g) CP26 to CP43; (h) CP29 to CP47. Chlorophylls *a* and *b* molecules are rendered as green and blue sticks, respectively. Interface chlorophylls implicated in intermolecular energy transfer are highlighted with thickened sticks and labeled. Mg-to-Mg distances (Å) between adjacent interfacial chlorophylls pairs are annotated with red numbers and connected by dark red dashed lines.

Supplementary Fig. 9

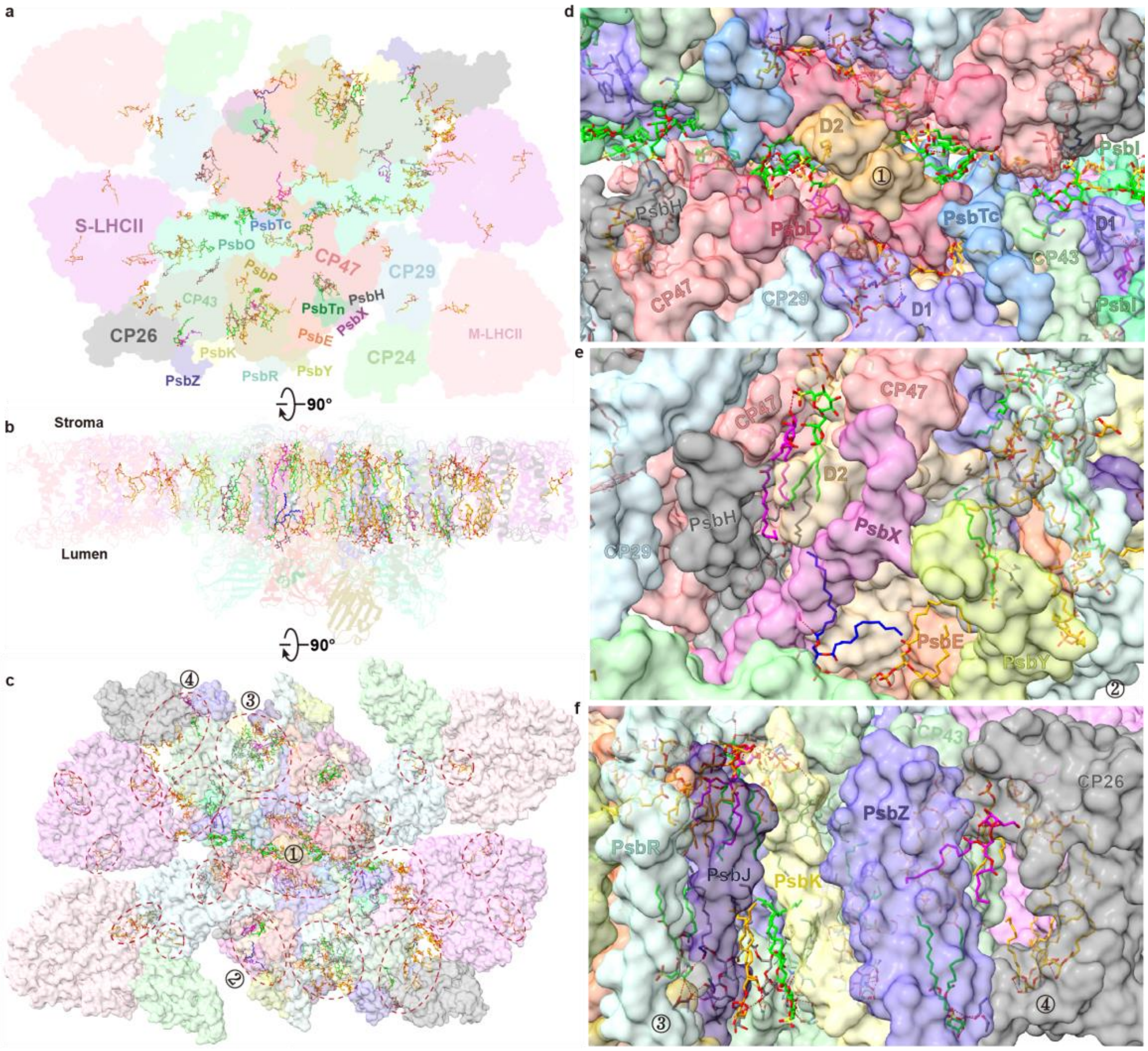

Supplementary Fig. 9 | Native lipid architecture and interaction networks in the $C_2S_2M_2$ region of the PSII-LHCII supercomplex.

a, Structural overview of 111 resolved native lipids in the $C_2S_2M_2$ region (PDB 21WV) viewed from the stromal side, comprising LHG (orange, n=66), SQD (lime, n=23), DGD (gray, n=13), MGE (magenta, n=5), LNL (dim gray, n=3) and 3PH (blue, n=1) molecules. Protein subunits are shown as flat surface representations with coloring consistent with Fig. 2. All lipid molecules are indicated as sticks. **b,** Perpendicular side view of the lipid distribution, with protein subunits depicted as cartoons and lipid representations maintained as in a. **c,** Lumenal-side visualization of lipid-mediated

stabilization networks. Hydrogen-bonding interactions between lipid polar headgroups and protein residues/cofactors are shown with red dashed lines, with participating amino acids and cofactors depicted as sticks colored by their parent subunits. Protein subunits are shown as surface with 60% transparency. Regions comprising the protein–lipid–protein network are highlighted with dashed red ellipses. **e-f**, Magnified views of regions ①, ② and ③+④ in c, respectively. Endogenous lipid molecules are observed to mediate extensive protein–lipid–protein interactions through hydrogen-bonding networks. These lipids accumulate near chains PsbY, D2 and PsbX, as well as around PsbR, PsbE, PsbF and PsbJ, creating localized hydrophobic and dynamic environments that facilitate the exchange of Q_B and Q_C.

Supplementary Fig. 10

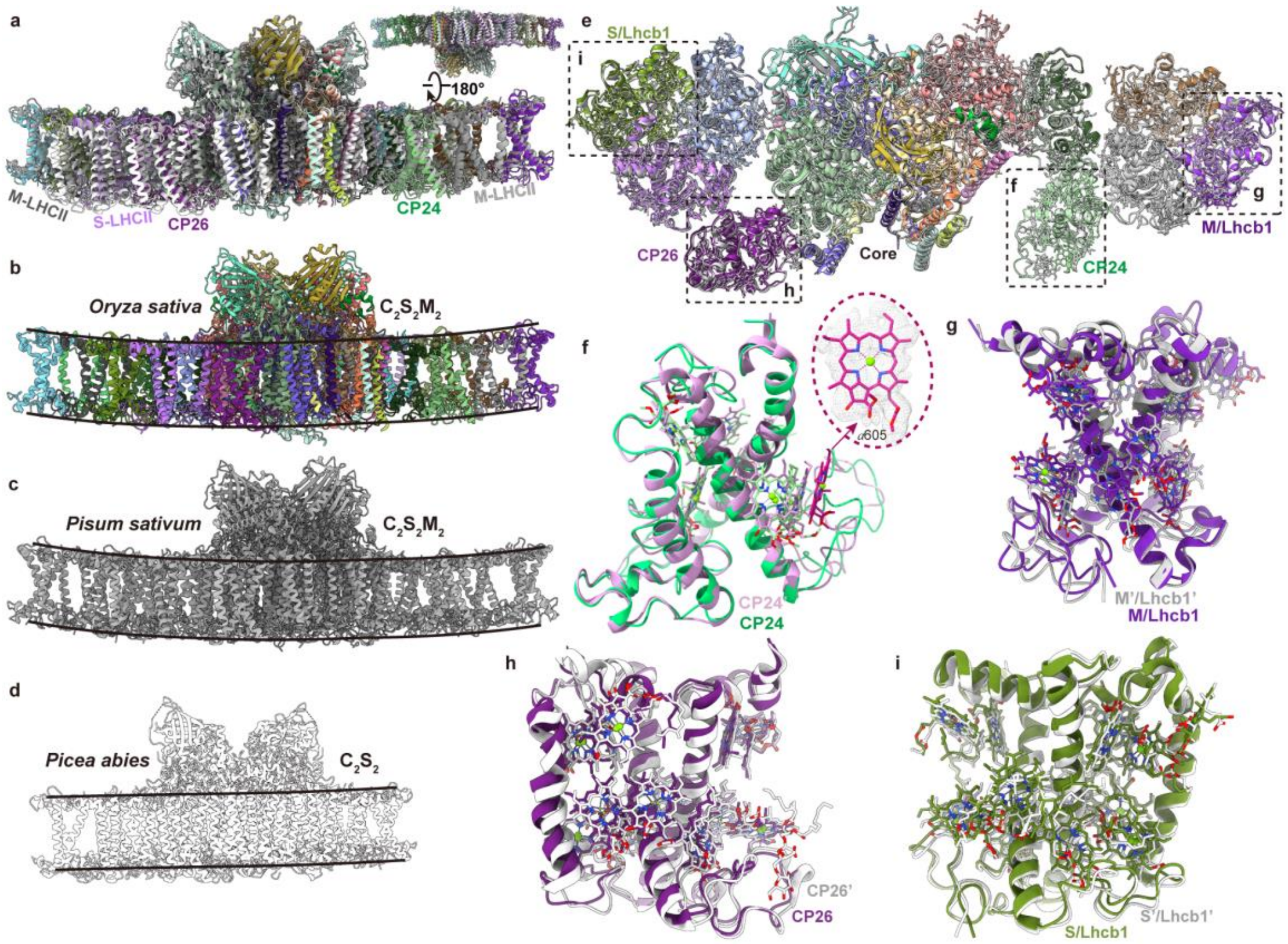

Supplementary Fig. 10 | Structural features of native thylakoid membrane curvature and pseudo-symmetry in PSII–LHCII supercomplexes. a, Side-view structural alignment of the in situ $C_2S_2M_2L_4$-type supercomplex (PDB 21WV) from *O. sativa* (coloured) with the purified $C_2S_2M_2$-type supercomplex from *P. sativum* (PDB 5XNL, dark gray) and the C_2S_2-type supercomplex from *P. abies* (PDB 8C29, white), aligned based on the D1 at one side. An overview from D1 alignment side is shown in the upper-right inset. **b**–**d**, Membrane curvature profiles in *O. sativa* (b), *P. sativum* (c), and *P. abies* (d), represented by contour lines tracing transmembrane helix positions. **e**, Structural superposition of the two half of $C_2S_2M_2L_4$-type supercomplex (PDB 21WV) following D1-based alignment, revealing a pseudo-two-fold symmetry with notable conformational divergence. **f**–**i**, Asymmetric structural features between monomers:

CP24 (f), the peripherally located Lhcb1 monomer in M-LHCII (g), CP26 (h), and the peripherally located Lhcb1 monomer in S-LHCII (i), showing differences in subunit conformation and ligand binding. In panel f, an additional Chl *a* molecule (medium violet red) is positioned at the CP24’ (plum) proximal to the core region, with its corresponding density map indicated by a dashed medium violet red ellipse.

Supplementary Fig. 11

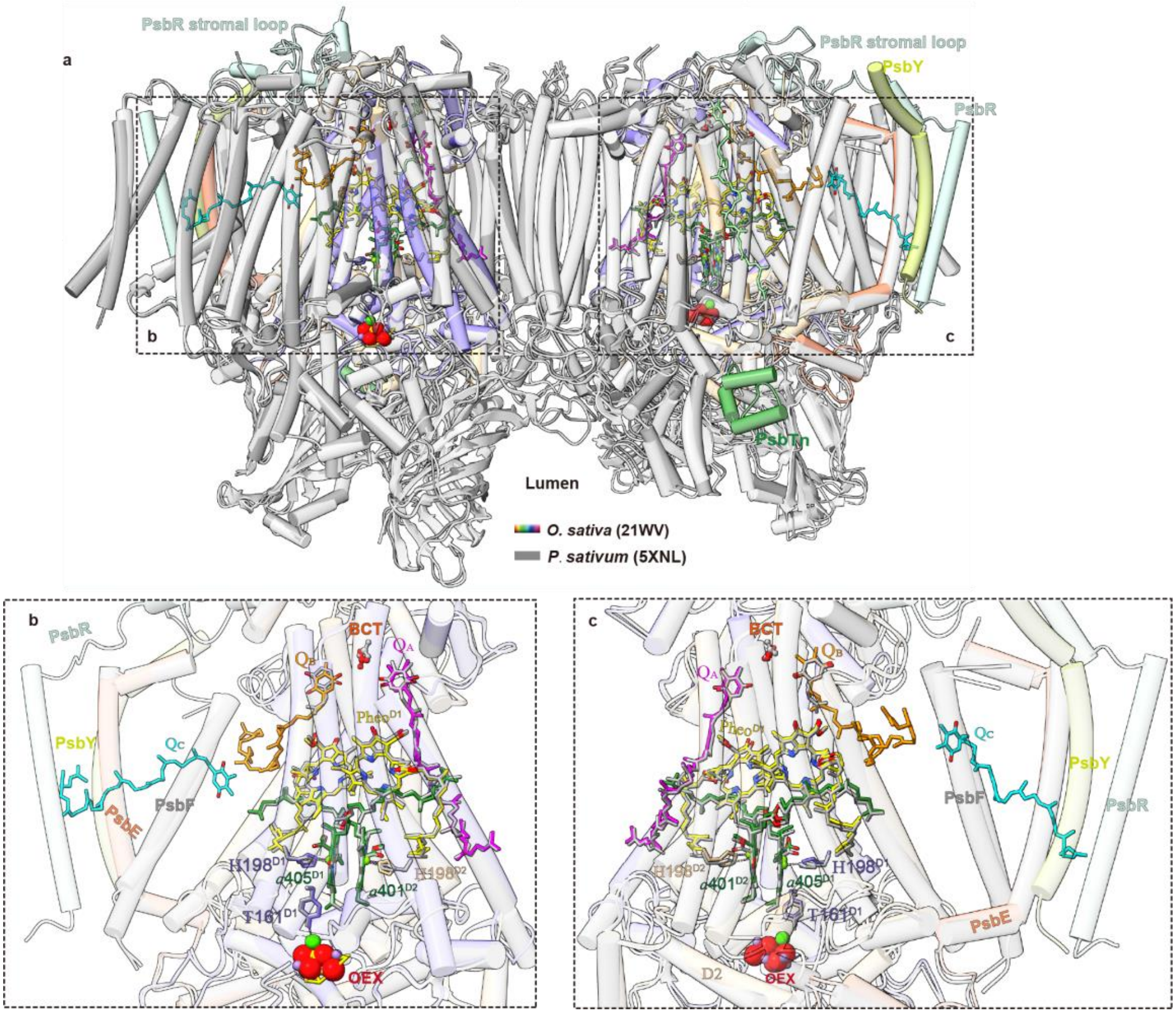

Supplementary Fig. 11 | Structural comparison of the electron transfer cofactor organizations of PSII–LHCII supercomplexes from *O. sativa* and *P. sativum*. a, The organization of electron transfer cofactors within the core of the *O. sativa* supercomplex (PDB 21WV, coloured) and their corresponding counterparts in the *P. sativum* structure (PDB 5XNL, light gray). As in Extended Data Fig. 3a,b, the alignment is based on one D1 subunit, **b**, **c**, Magnified views of structural features and cofactors involved in charge separation and electron transfer on the aligned side (b) and the opposite side (c). The corresponding regions are indicated by black dashed boxes in panle a.

Supplementary Fig. 12

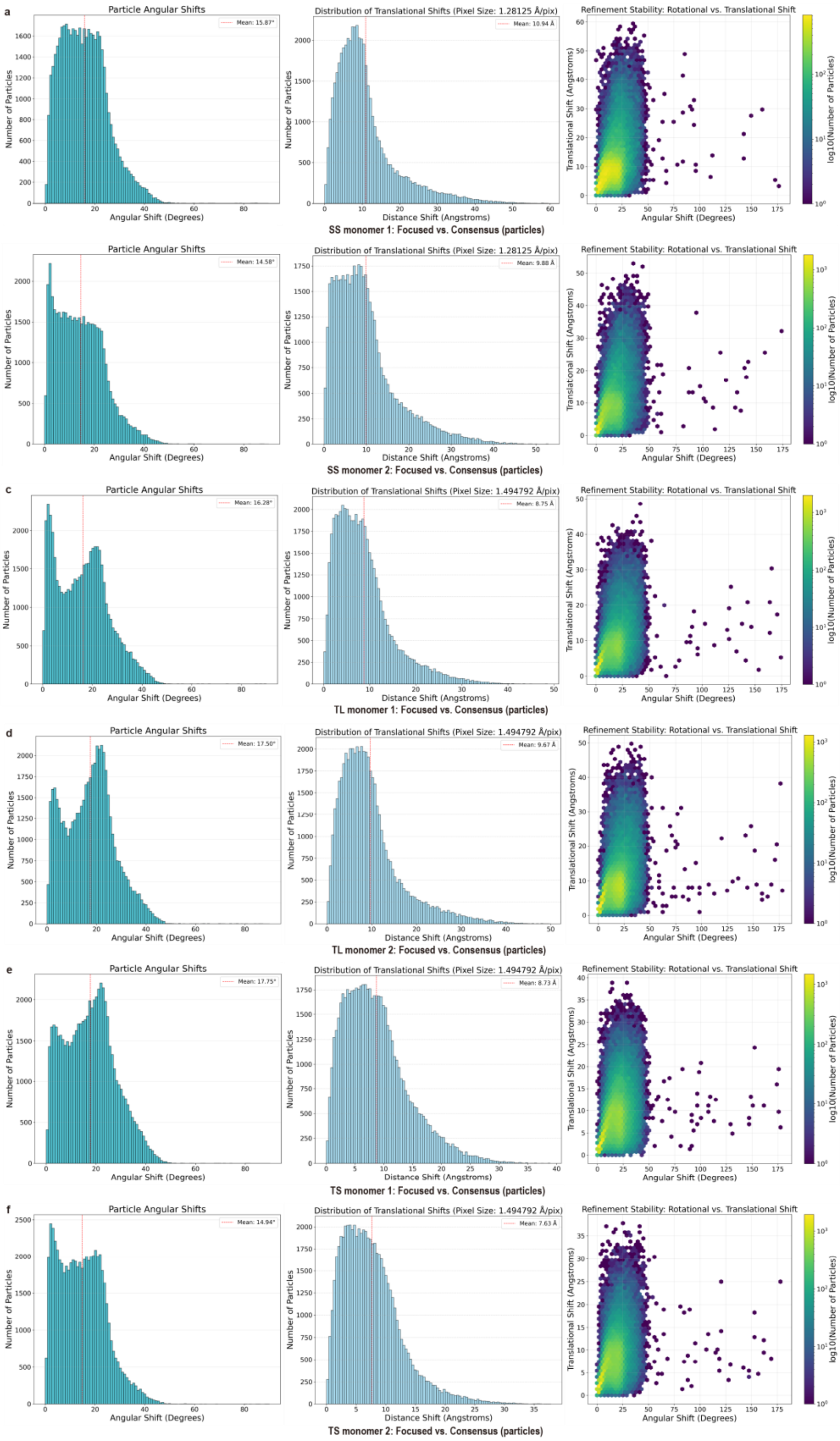

Supplementary Fig. 12 | Conformational heterogeneity of individual PSII monomers within dimeric assemblies.

Each panel shows the angular and translational shifts per particle between focused refinement of an individual PSII monomer and consensus refinement of the entire dimer. **a**, Comparison between focused refinement particles of SS dimer monomer 1 and consensus refinement particles of the SS dimer (n=48,475 particles). Mean angular shift, 15.87°; mean translational shift, 10.94 Å. **b**, Comparison between focused refinement particles of SS dimer monomer 2 and consensus refinement particles of the SS dimer (n=48,475 particles). Mean angular shift, 14.58°; mean translational shift, 9.88 Å. **c**, Comparison between focused refinement particles of TL dimer monomer 1 and consensus refinement particles of the TL dimer (n=52,794 particles). Mean angular shift, 16.28°; mean translational shift, 8.75 Å. **d**, Comparison between focused refinement particles of TL dimer monomer 2 and consensus refinement particles of the TL dimer (n=52,794 particles). Mean angular shift, 17.50°; mean translational shift, 9.67 Å. **e**, Comparison between focused refinement particles of TS dimer monomer 1 and consensus refinement particles of the TS dimer (n=61,120 particles). Mean angular shift, 17.75°; mean translational shift, 8.73 Å. **f**, Comparison between focused refinement particles of TS dimer monomer 2 and consensus refinement particles of the TS dimer (n=61,120 particles). Mean angular shift, 14.94°; mean translational shift, 7.63 Å. Angular shifts (teal histograms) were calculated as the geodesic distance between orientations; translational shifts (skyblue histograms) were calculated as the Euclidean distance between translation vectors. Red dashed lines indicate mean values. All histograms use 100 bins.

Supplementary Fig. 13

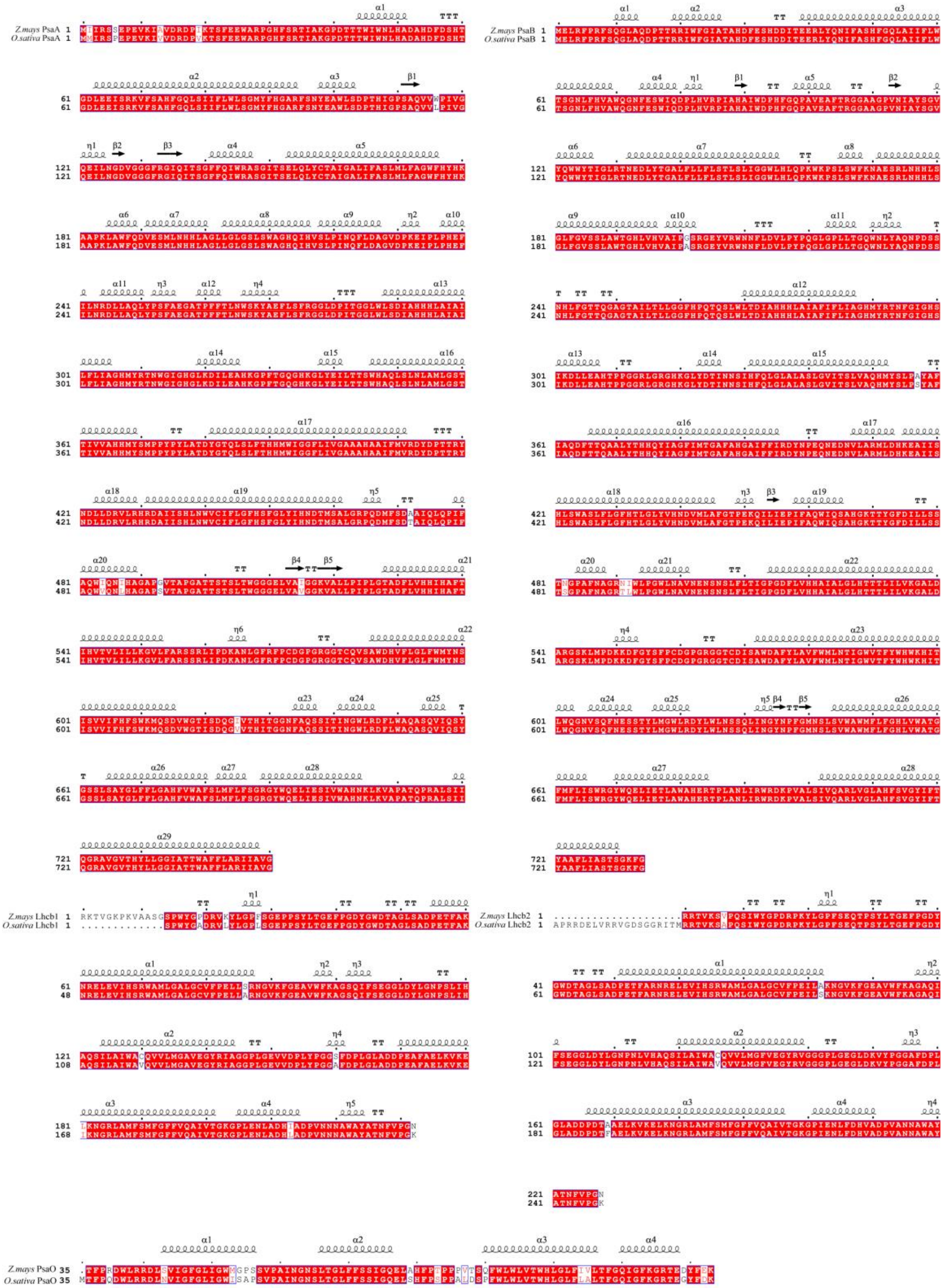

Supplementary Fig. 13 | Conserved sequences of photosynthetic complexes in *Oryza sativa* and *Zea mays*.

Sequence alignments of the PSI-LHCI-LHCII supercomplex core subunits subunits (PsaA, PsaB, and PsaO) and the LHCII trimer from *Oryza sativa* (below) and *Zea mays* (above) show high conservation between the two species, indicating that the observed conformational differences are not due to species-specific sequence variations.

Supplementary Fig. 14

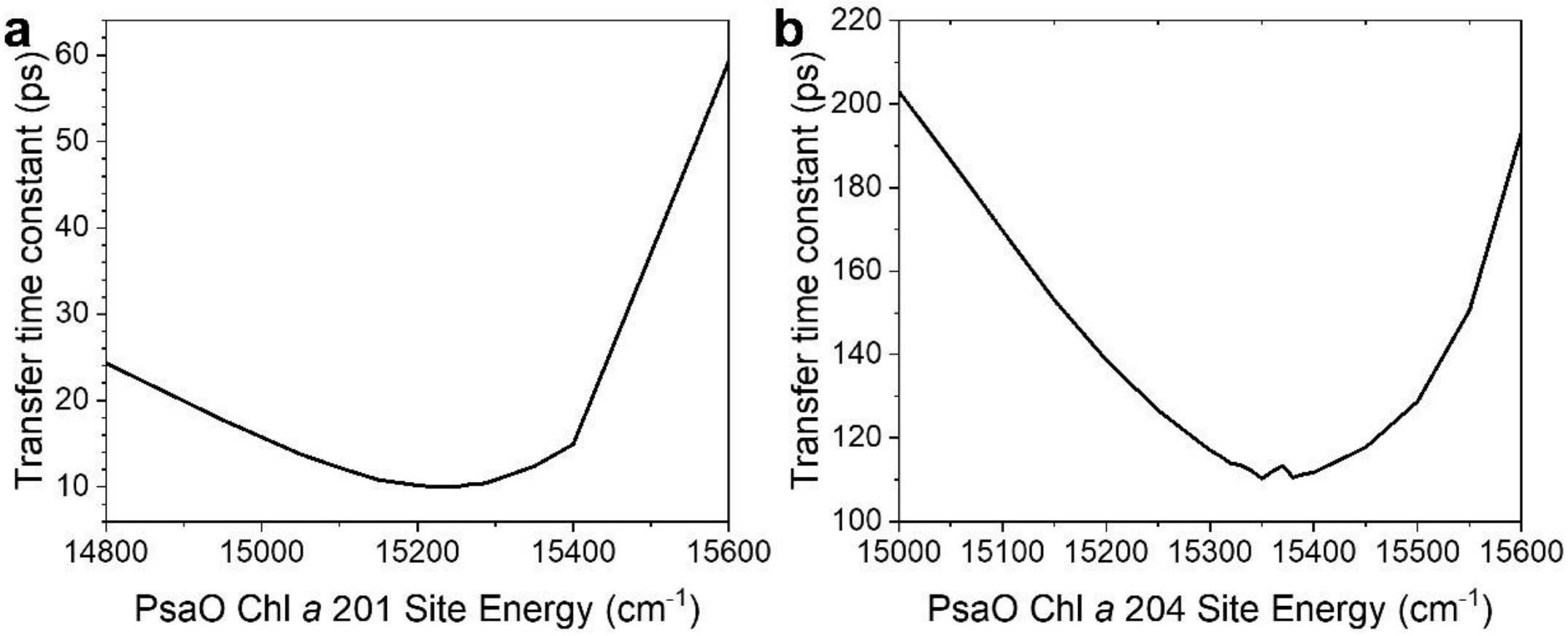

Supplementary Fig. 14 | Main excitation energy transfer time constants for transfer from LHCII to PSI.

a, EET time constant for transfer from the LHCII Chl *a* 610-611-612 cluster to PsaO Chl *a*201 as a function of the site energy of the latter Chl, **b** and for the transfer of the LHCII Chl *a* 613-614 cluster to PsaO Chl *a*204.

Supplementary Fig. 15

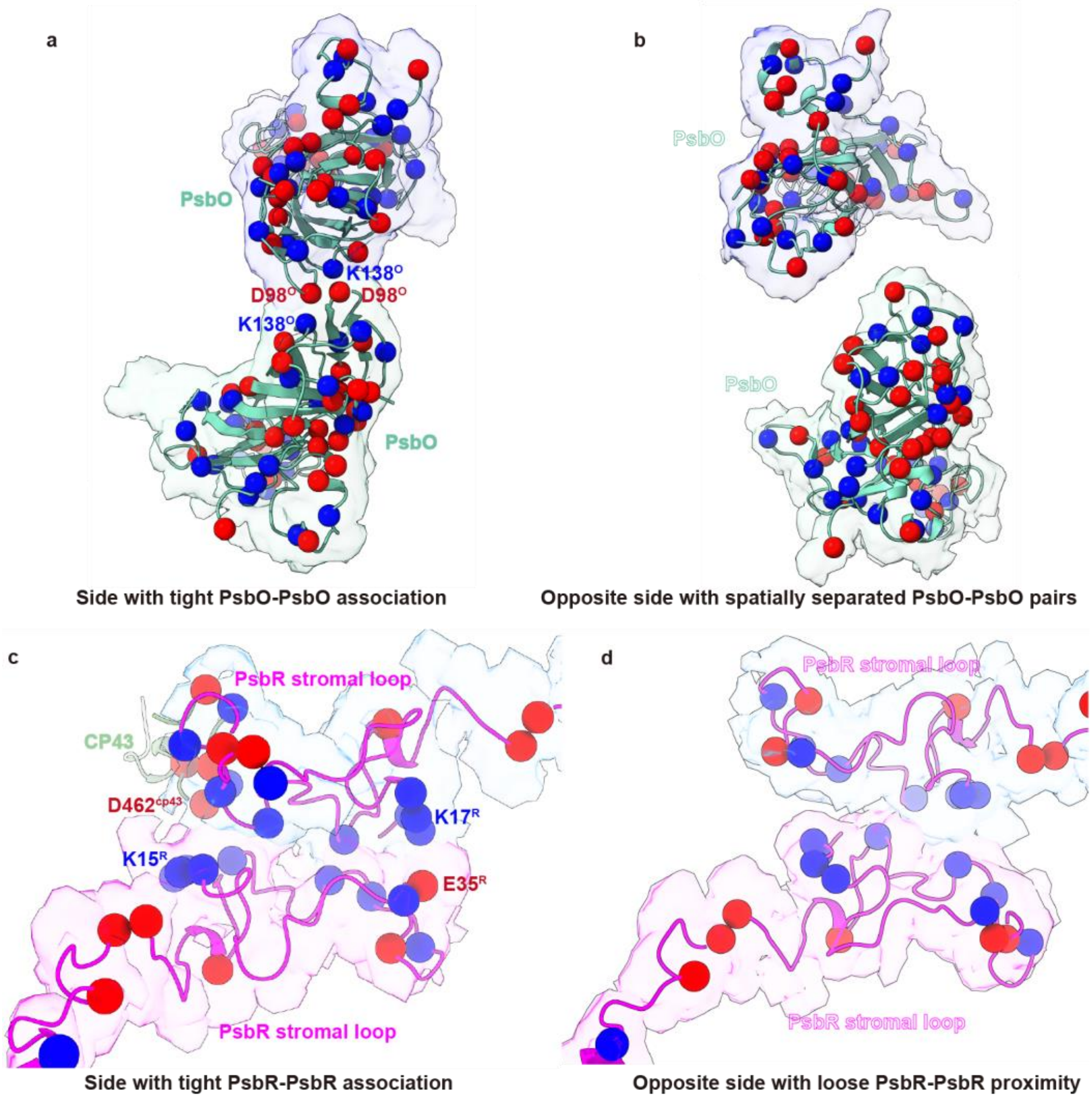

Supplementary Fig. 15 | Interfaces of TL and TS dimers.

a, The interface of the side with tight PsbO-PsbO association in the TL dimer. PsbO subunits from the two PSII units that form the TL dimer are shown as turquoise cartoon on turquoise and light purple backgrounds, respectively. Cα atoms of positively charged basic residues (lysine and arginine) and negatively charged acidic residues (aspartate and glutamate) on the PsbO subunits are shown as blue and red spheres, respectively. Spatially proximal charged residues with potential electrostatic interactions, K138^{O} and D98^{O}, are labelled in blue and red, respectively. **b**, The interface of the opposite side with spatially separated PsbO-PsbO pairs in the TL dimer. Representation as in panel

a. **c**, The interface of the side with tight PsbR-PsbR association in the TS dimer. PsbR subunits from the two PSII units that form the TS dimer are shown as magenta cartoon on magenta and light blue backgrounds, respectively. Cα atoms of positively charged basic residues (lysine and arginine) and negatively charged acidic residues (aspartate and glutamate) on the PsbR subunits are shown as blue and red spheres, respectively. Spatially proximal charged residues with potential electrostatic interactions are labelled in blue and red. **d**, The interface of the opposite side with loose PsbR-PsbR proximity in the TS dimer. Representation as in panel c.

Supplementary Tables

Supplementary Table 1. The protein and cofactor components in the final structural model of *Oryza sativa* $C_2S_2M_2L_4$-type PSII-LHCII supercomplex.

Gene	Chain name	Range of amino acid residues traced	Chlorophylls *	Carotenoids *	Lipids *	Others *
D1	1A,1a	11-344	8 Chl *a*<br>4 PHO	2 BCR	6 SQD<br>4 LHG<br>1 MGE	2 FE2<br>4 CL<br>2 OEX<br>3 PL9
CP47	1B,1b	2-504	32 Chl *a*	6 BCR	8 SQD<br>6 LHG<br>1 DGD<br>1 MGE	2 PL9
CP43	1C,1c	24-473	26 Chl *a*	8 BCR	4 SQD<br>3 LNL<br>7 DGD<br>11 LHG<br>1 MGE	
D2	1D,1d	13-353	4 Chl *a*	2 BCR	3 SQD<br>7 LHG<br>1 3PH<br>1 MGE	2 BCT<br>3 PL9
PsbE	1E,1e	8-82			1 LHG	
PsbF	1F,1f	4-39				2 HEM
PsbH	1H,1h	13-72		2 BCR	2 DGD	
PsbI	1I,1i	1-34			1 LHG<br>1 SQD	
PsbJ	1J,1j	6-40			3 LHG	
PsbK	1K,1k	25-61				
PsbL	1L,1l	2-38			2 LHG	
PsbM	1M,1m	1-33				
PsbO	1O,1o	1-248				
PsbP	1P,1p	1-186			1 LHG	
CP29	1R,1r	11-244	20 Chl *a*<br>8 Chl *b*	2 LUT<br>2 XAT	2 DGD<br>4 LHG	
CP26	1S,1s	19-236	18 Chl *a*<br>8 Chl *b*	4 LUT	1 DGD<br>5 LHG	
CP24	04, 08	5-209	13 Chl *a*<br>10 Chl *b*	1 BCR		
PsbTc	1T,1t	1-32		2 BCR		

PsbTn	1U,1u	107-133			
PsbR	1V,1v	7-111			2 LHG
PsbW	1W,1w	1-54			4 LHG<br>1 SQD
PsbX	1X,1x	45-83			
PsbY	1Y,1y	2-37			
PsbZ	1Z,1z	1-62			1 MGE
S-LHCII	00,09,1N,1n	13-231	32 Chl *a*<br>22 Chl *b*	6 LUT<br>2 XAT<br>2 VIV	12 LHG
	1G,1g	13-231	16 Chl *a*<br>12 Chl *b*	4 LUT<br>2 XAT	2 LHG
M-LHCII	01,02,05,06	13-231	29 Chl *a*<br>22 Chl *b*	3 LUT	
	03,07	23-242	15 Chl *a*<br>10 Chl *b*	2 LUT	
L-LHCII	11,13,14,16,17,19<br>20,22	14-231			
	12,15,18,21	7-228			

* The values for chlorophylls, carotenoids, lipids, and other ligands are the combined sum for the two subunits within the supercomplex. The two subunits are not completely symmetrical in composition.

Supplementary Table 2. The protein and cofactor components in the final structural model of *Oryza sativa* PSI-LHCI-LHCII supercomplex.

Gene	Chain name	Range of amino acid residues traced	Chlorophylls	Carotenoids	Lipids	Others
PsaA	A	7-750	44 Chl *a*<br>1 Cl0	7 BCR	5 LHG<br>1 MGE	1 SF4<br>1 PQN
PsaB	B	1-734	41 Chl *a*	8 BCR	3 LHG<br>2 DGD	1 PQN
PsaC	C	1-81				2 SF4
PsaD	D	63-205				
PsaE	E	69-136				
PsaF	F	6778-224	3 Chl *a*	2 BCR	2 MGE	
PsaG	G	49-145	3 Chl *a*	1 BCR	2 LHG<br>1 MGE	
PsaH	H	48-142	1 Chl *a*	1 BCR		
PsaI	I	5-34		1 BCR		
PsaJ	J	1-44	1 Chl *a*	1 BCR	1 DGD	
PsaK	K	46-134	4 Chl *a*	1 BCR		
PsaL	L	47-211	3 Chl *a*	2 BCR		
PsaN	N	62-145	1 Chl *a*			
PsaO	O	35-127	4 Chl *a*	2 BCR	2 LHG	
PsaG	G	45-141	3 Chl *a*	1 BCR	2 LHG<br>1 MGE	
Lhcb1	X	14-231	8 Chl *a*<br>6 Chl *b*		1 LHG	
Lhcb2	Y	7-228	8 Chl *a*<br>6 Chl *b*			
Lhcb1	Z	14-231	8 Chl *a*<br>6 Chl *b*			
Lhca1	1	38-237	12 Chl *a*<br>2 Chl *b*	1 BCR<br>1 LUT<br>1 XAT	1 LHG	
Lhca2	2	59-267	10 Chl *a*<br>5 Chl *b*	1 BCR<br>1 LUT<br>1 XAT	6 LHG	
Lhca3	3	53-275	13 Chl *a*<br>1 Chl *b*	1 BCR<br>1 LUT<br>1 XAT	1 LHG<br>2 MGE	
Lhca4	4	54-252	13 Chl *a*<br>4 Chl *b*	1 BCR<br>2 LUT<br>1 XAT	2 LHG	

Supplementary Table 3. Cryo-EM data collection, refinement and validation statistics for $C_2S_2M_2L_4$ PSII-LHCII and three types of PSII-LHCII dimers.

	$C_2S_2M_2L_4$ (EMD-68060) (PDB 21WV)	SS dimer (EMD-81438) (PDB 27UR)	TL dimer (EMD-81447) (PDB 27UT)	TS dimer (EMD-68048) (PDB 21WH)
**Data collection and processing**				
Magnification	105,000×	105,000×	105,000×	105,000×
Voltage (kV)	300	300	300	300
Electron exposure ($e^-/Å^2$)	55	55	55	55
Defocus range (μm)	−1.4 to −2.2	−1.4 to −2.2	−1.4 to −2.2	−1.4 to −2.2
Pixel size (Å)	0.82	1.28	1.49	1.49
Symmetry imposed	C1	C1	C1	C1
Initial particle images (no.)	1,623,088	349,186	521,036	240,082
Final particle images (no.)	172,752	60,034	38,275	75,119
Map resolution (Å)	2.89*	4.25, 3.83*	3.93, 3.84*	3.92, 3.53*
FSC threshold	0.143	0.143	0.143	0.143
**Refinement**				
Initial model used	PDB 5XNL	PDB 21WV	PDB 21WV	PDB 21WV
Model resolution (Å)	2.70	2.89	2.89	2.89
FSC threshold	0.143	0.143	0.143	0.143
Map sharpening B factor ($Å^2$)	45.7	55.8	21.2	27.5
Model composition				
Nonhydrogen atoms	94721	-	-	-
Protein residues	9388	-	-	-
Ligands	492	-	-	-
B factors ($Å^2$)				
Protein	44.69	-	-	-
Ligand	47.83	-	-	-
R.M.S. deviations				
Bond lengths (Å)	0.010	-	-	-
Bond angles (°)	5.658	-	-	-
**Validation**				
MolProbity score	2.12	-	-	-
Clashscore	12.44	-	-	-
Poor rotamers (%)	0.78	-	-	-

Ramachandran plot				
Favored (%)	91.30	-	-	-
Allowed (%)	8.27	-	-	-
Disallowed (%)	0.43	-	-	-

*Determined with cryoSPARC. For the L4 region of the $C_2S_2M_2L_4$ model, the LHCII models from PSI-LHCI-LHCII (side chains and ligands removed) were used to fit the distinct triangular densities. PSII-LHCII dimers were obtained by fitting the refined $C_2S_2M_2$ model (side chains and ligands removed) into the densities of individual PSII units.

Supplementary Table 4. Cryo-EM data collection, refinement and validation statistics for TL-TS PSII-LHCII trimer and TL-TS-SS and TL-TS-TL PSII-LHCII tetramers.

	TL-TS trimer (EMD-68049) (PDB 21WI)	TL-TS-SS tetramer (EMD-68047) (PDB 21WG)	TL-TS-TL tetramer (EMD-68043) (PDB-21WD)
**Data collection and processing**			
Magnification	105,000×	105,000×	105,000×
Voltage (kV)	300	300	300
Electron exposure ($e^-/Å^2$)	55	55	55
Defocus range (μm)	−1.4 to −2.2	−1.4 to −2.2	−1.4 to −2.2
Pixel size (Å)	1.49	1.70	1.44
Symmetry imposed	C1	C1	C1
Initial particle images (no.)	240,082	387,022	373,192
Final particle images (no.)	37,107	45,682	83,210
Map resolution (Å)	4.27, 3.79, 4.07*	4.28, 3.6, 4.43, 4.3*	4.11, 4.24, 3.48, 3.95*
FSC threshold	0.143	0.143	0.143
**Refinement**			
Initial model used	PDB 21WV	PDB 21WV	PDB 21WV
Model resolution (Å)	2.89	2.89	2.89
FSC threshold	0.143	0.143	0.143
Map sharpening B factor ($Å^2$)	28.4	42.3	40.0
Model composition			
Nonhydrogen atoms	-	-	-
Protein residues	-	-	-
Ligands	-	-	-
B factors ($Å^2$)			
Protein	-	-	-
Ligand	-	-	-
R.M.S. deviations			
Bond lengths (Å)	-	-	-
Bond angles (°)	-	-	-
**Validation**			
MolProbity score	-	-	-
Clashscore	-	-	-
Poor rotamers (%)	-	-	-

Ramachandran plot			
Favored (%)	-	-	-
Allowed (%)	-	-	-
Disallowed (%)	-	-	-

*Determined with cryoSPARC. PSII assemblies (trimers and tetramers) were obtained by fitting the refined $C_2S_2M_2$ model (side chains and ligands removed) into the densities of individual PSII units.

Supplementary Table 5. Cryo-EM data collection, refinement and validation statistics for $C_2S_2M_2L_4$ PSII-LHCII composite.

	Core (promoter 1 and 2) (EMD-66946,66947) (PDB 9XK3,9XK4)	S-LHCII/CP26 (promoter 1 and 2) (EMD-66949,66950) (PDB 9XK6,9XK7)	M-LHCII/CP29-CP24 (promoter 1 and 2) (EMD-66951,66952) (PDB 9XK8,9XK9)	L-LHCII (promoter 1 and 2) (EMD-66971,66974)
**Data collection and processing**				
Magnification	105,000×	105,000×	105,000×	105,000×
Voltage (kV)	300	300	300	300
Electron exposure ($e^-/Å^2$)	55	55	55	55
Defocus range (μm)	−1.4 to −2.2	−1.4 to −2.2	−1.4 to −2.2	−1.4 to −2.2
Pixel size (Å)	0.82	0.82	0.82	1.28
Symmetry imposed	C1	C1	C1	C1
Initial particle images (no.)	1,623,088	1,623,088	1,623,088	1,623,088
Final particle images (no.)	173,752	172,752	172,752	172,752
Map resolution (Å)	2.7*, 2.78*	3.27*, 3.25*	3.59*, 3.64*	5.23*, 5.04*
FSC threshold	0.143	0.143	0.143	0.143
**Refinement**				
Initial model used	PDB 5XNL	PDB 5XNL	PDB 5XNL	
Model resolution (Å)	2.70	2.70	2.70	
FSC threshold	0.143	0.143	0.143	
Map sharpening B factor ($Å^2$)	52.0, 48.3	75.9, 70.0	90.4, 93.0	
Model composition				
Nonhydrogen atoms	26177, 25635	10178, 9951	12015, 10982	-
Protein residues	2751, 2725	875, 874,	1071, 1076	-
Ligands	103, 97	76, 71	77, 68	-
B factors ($Å^2$)				
Protein	80.71, 74.48	57.11, 50.65,	87.41, 90.31	-
Ligand	78.35, 72.13	52.58, 46.30,	82.18, 84.96	-

R.M.S. deviations				
Bond lengths (Å)	0.007, 0.007	0.011, 0.011,	0.014, 0.014	-
Bond angles (°)	4.181, 4.210	6.837, 6.911,	7.025, 6.997	-
**Validation**				
MolProbity score	1.90, 1.85	2.15, 2.12,	2.34, 2.17	-
Clashscore	8.77, 8.19	12.56, 11.21,	11.24, 10.75	-
Poor rotamers (%)	0.36, 0.36	0.45, 0.75,	1.47, 0.77	-
Ramachandran plot				
Favored (%)	93.34, 94.03	90.40, 89.93,	85.71, 87.19	-
Allowed (%)	6.44, 5.71	9.13, 9.72,	13.34, 11.77	-
Disallowed (%)	0.22, 0.26	0.46, 0.35,	0.95, 1.04	-

*Determined with cryoSPARC.

Supplementary Table 6. Cryo-EM data collection, refinement and validation statistics for PSI supercomplexes.

	**PSI-LHCI-LHCII (EMD-66932) (PDB 9XJ9)**	**PSI-LHCI (EMD-66925) (PDB 9XJ1)**
**Data collection and processing**		
Magnification	105,000×	105,000×
Voltage (kV)	300	300
Electron exposure ($e^-/Å^2$)	55	55
Defocus range (μm)	−1.4 to −2.2	−1.4 to −2.2
Pixel size (Å)	0.82	0.82
Symmetry imposed	C1	C1
Initial particle images (no.)	531,557	531,557
Final particle images (no.)	124,514	182,666
Map resolution (Å)	2.96*	2.38*
FSC threshold	0.143	0.143
**Refinement**		
Initial model used	PDB 5ZJI	PDB 6YEZ
Model resolution (Å)	3.30	2.70
FSC threshold	0.143	0.143
Map sharpening B factor ($Å^2$)	45.5	53.6
Model composition		
Nonhydrogen atoms	43661	39155
Protein residues	4096	3440
Ligands	284	244
B factors ($Å^2$)		
Protein	81.60	8.90
Ligand	85.01	8.80
R.M.S. deviations		
Bond lengths (Å)	0.012	0.010
Bond angles (°)	7.104	6.891
**Validation**		
MolProbity score	1.96	2.08
Clashscore	12.17	11.97
Poor rotamers (%)	0.77	1.82
Ramachandran plot		
Favored (%)	94.67	95.83
Allowed (%)	5.11	3.88
Disallowed (%)	0.22	0.29

*Determined with cryoSPARC.

Supplementary Table 7. Simulation Parameters.

	RC[a]		CP43[b]/CP47[c]	CP29[d]		LHCII/CP26/CP24[e]	
**Pigment type**	Chl *a*	Pheo	Chl *a*	Chl *a*	Chl *b*	Chl *a*	Chl *b*
**TDM[f]**	4.4	3.5	4.4	3.74	3.18	4.0	3.4
$\boldsymbol{\sigma_{inhom}}$[g]	200	120	180	90	108	80	96
**SD type[h]**	a	a	b	c	d	e	f

[a] Raszewski et al. (2005, 2008a), adapted by Bennett et al. (2013).

[b] Müh et al. (2012), adapted by Bennett et al. (2013).

[c] Raszewski et al. (2008b), adapted by Bennett et al. (2013).

[d] Mascoli et al. (2020), adapted by Leonardo et al. (2024).

[e] Novoderezhkin et al. (2011), adapted by Bennett et al. (2013).

[f] Transition Dipole Moment magnitude in Debye.

[g] Inhomogeneous broadening full-width half maximum in cm^{-1}.

[h] Spectral density type (see Supplementary Table 8).

Supplementary Table 8. Spectral Density Parameters.

SD type	a	b	c	d	e	f
S_0	0.65	0.5				
s_1	0.8	0.8				
s_2	0.5	0.5				
ω_1 (cm^{-1})	0.532	0.532				
ω_2 (cm^{-1})	1.94	1.94				
λ_0 (cm^{-1})			40	48	37	48
γ_0 (cm^{-1})			40	40	30	30
γ_{vib} (cm^{-1})			3	3	3	3
ΣS_j [a]			0.56	0.67	0.70	0.91

[a] Total Huang-Rhys factor used for each spectral density, see equation 2. The frequencies ω_j and corresponding Huang-Rhys factors S_j for SD type e are reported in [20]. The same frequencies ω_j are used for the SD types c, d and f, but their Huang-Rhys factors S_j have been rescaled to match the reported ΣS_j.

Supplementary Videos legends

Supplementary Video 1. In situ structure of the $C_2S_2M_2L_4$-type PSII-LHCII supercomplex.

Supplementary Video 2. In situ structure of the PSI-LHCI-LHCII supercomplex.

Supplementary Video 3. Structural characterizations of three distinct in situ PSII-LHCII dimers.

Supplementary Video 4. Proposed schematic model of grana architecture shaped by $C_2S_2M_2L_4$ supercomplexes.

56 Guskov, A. *et al.* Cyanobacterial photosystem II at 2.9-A resolution and the role of quinones, lipids, channels and chloride. *Nat Struct Mol Biol* **16**, 334-342, doi:10.1038/nsmb.1559 (2009).

57 Goddard, T. D. *et al.* UCSF ChimeraX: Meeting modern challenges in visualization and analysis. *Protein Sci* **27**, 14-25, doi:10.1002/pro.3235 (2018).

58 Raszewski, G., Saenger, W. & Renger, T. Theory of optical spectra of photosystem II reaction centers: location of the triplet state and the identity of the primary electron donor. *Biophys J* **88**, 986-998, doi:10.1529/biophysj.104.050294 (2005).

59 Raszewski, G., Diner, B. A., Schlodder, E. & Renger, T. Spectroscopic properties of reaction center pigments in photosystem II core complexes: revision of the multimer model. *Biophys J* **95**, 105-119, doi:10.1529/biophysj.107.123935 (2008).

60 Raszewski, G. & Renger, T. Light harvesting in photosystem II core complexes is limited by the transfer to the trap: can the core complex turn into a photoprotective mode? *J Am Chem Soc* **130**, 4431-4446, doi:10.1021/ja7099826 (2008).

61 Novoderezhkin, V., Marin, A. & van Grondelle, R. Intra- and inter-monomeric transfers in the light harvesting LHCII complex: the Redfield-Förster picture. *Phys Chem Chem Phys* **13**, 17093-17103, doi:10.1039/c1cp21079c (2011).

62 Müh, F., Madjet Mel, A. & Renger, T. Structure-based simulation of linear optical spectra of the CP43 core antenna of photosystem II. *Photosynth Res* **111**, 87-101, doi:10.1007/s11120-011-9675-8 (2012).

63 Mascoli, V., Novoderezhkin, V., Liguori, N., Xu, P. & Croce, R. Design principles of solar light harvesting in plants: Functional architecture of the monomeric antenna CP29. *Biochim Biophys Acta Bioenerg* **1861**, 148156, doi:10.1016/j.bbabio.2020.148156 (2020).

64 Madjet, M. E., Abdurahman, A. & Renger, T. Intermolecular coulomb couplings from ab initio electrostatic potentials: application to optical transitions of strongly coupled pigments in photosynthetic antennae and reaction centers. *J Phys Chem B* **110**, 17268-17281, doi:10.1021/jp0615398 (2006).

65 Seidl, F. Parametrization of Exciton Hamiltonian of Light-Harvesting Antennae CP24, CP26 and CP29 in Photosystem II/Author Florian Seidl, BSc MSc. (2024).

66 Renger, T. *et al.* Refinement of a structural model of a pigment-protein complex by accurate optical line shape theory and experiments. *J Phys Chem B* **111**, 10487-10501, doi:10.1021/jp0717241 (2007).

67 Chmeliov, J., Trinkunas, G., van Amerongen, H. & Valkunas, L. Light harvesting in a fluctuating antenna. *J Am Chem Soc* **136**, 8963-8972, doi:10.1021/ja5027858 (2014).

68 Belgio, E., Johnson, M. P., Jurić, S. & Ruban, A. V. Higher plant photosystem II light-harvesting antenna, not the reaction center, determines the excited-state lifetime-both the maximum and the nonphotochemically quenched. *Biophys J* **102**, 2761-2771, doi:10.1016/j.bpj.2012.05.004 (2012).

69 Novoderezhkin, V. I., Palacios, M. A., Van Amerongen, H. & Van Grondelle, R. Energy-transfer dynamics in the LHCII complex of higher plants: modified redfield approach. *J Phys Chem B* **108**, 10363-10375, doi:10.1021/jp0496001 (2004).
