# Supplementary material for: In situ structures of plant photosystem supercomplexes
Source: Nature. 2026 Jul 29;657(8133):1094–103. doi: 10.1038/s41586-026-10847-3 (PMC13600941; doi:10.1038/s41586-026-10847-3)
Supplement: Supplementary file 2 [file 41586_2026_10847_MOESM2_ESM.pdf]

nature portfolio

Corresponding author(s): Jiapeng Zhu; Roberta Croce; Kai zhang; Jiao Li

Last updated by author(s): Jun 12, 2026

Reporting Summary

Nature Portfolio wishes to improve the reproducibility of the work that we publish. This form provides structure for consistency and transparency in reporting. For further information on Nature Portfolio policies, see our Editorial Policies and the Editorial Policy Checklist.

Statistics

For all statistical analyses, confirm that the following items are present in the figure legend, table legend, main text, or Methods section.

n/a	Confirmed	
☐	☒	The exact sample size (*n*) for each experimental group/condition, given as a discrete number and unit of measurement
☐	☒	A statement on whether measurements were taken from distinct samples or whether the same sample was measured repeatedly
☒	☐	The statistical test(s) used AND whether they are one- or two-sided<br>*Only common tests should be described solely by name; describe more complex techniques in the Methods section.*
☒	☐	A description of all covariates tested
☒	☐	A description of any assumptions or corrections, such as tests of normality and adjustment for multiple comparisons
☒	☐	A full description of the statistical parameters including central tendency (e.g. means) or other basic estimates (e.g. regression coefficient) AND variation (e.g. standard deviation) or associated estimates of uncertainty (e.g. confidence intervals)
☒	☐	For null hypothesis testing, the test statistic (e.g. F, t, r) with confidence intervals, effect sizes, degrees of freedom and P value noted<br>*Give P values as exact values whenever suitable.*
☒	☐	For Bayesian analysis, information on the choice of priors and Markov chain Monte Carlo settings
☒	☐	For hierarchical and complex designs, identification of the appropriate level for tests and full reporting of outcomes
☒	☐	Estimates of effect sizes (e.g. Cohen's d, Pearson's r), indicating how they were calculated

Our web collection on statistics for biologists contains articles on many of the points above.

Software and code

Policy information about availability of computer code

Data collection	EPU 2.7. This software is also mentioned in the manuscript as well.
Data analysis	CryoSPARC 4.7.0, Gautomatch 0.60, Coot 0.9.8.1, MolProbity 4.5.2, ChimeraX 1.6.1, PyMOL 2.5.4, Phenix 1.20.1, IMOD 4.9.12, Movavi Video Editor (26.17.0), Python 3.12 (script for calculating the simplified Förster energy transfer rate matrix is available at https://zenodo.org/records/13283796), MatlabR2024b (script for the calculation of the modified Redfield/generalized Förster energy transfer rate matrix is available at https://zenodo.org/records/13346121), Scripts for quantifing PSII dimer structural heterogeneity (analyze_refine.py and calcu_angles.py) are available at https://github.com/JackZhang-Lab/EM-scripts. These softwares/codes are also mentioned in the manuscript as well.

For manuscripts utilizing custom algorithms or software that are central to the research but not yet described in published literature, software must be made available to editors and reviewers. We strongly encourage code deposition in a community repository (e.g. GitHub). See the Nature Portfolio guidelines for submitting code & software for further information.

Data

Policy information about availability of data

All manuscripts must include a data availability statement. This statement should provide the following information, where applicable:

- Accession codes, unique identifiers, or web links for publicly available datasets
- A description of any restrictions on data availability
- For clinical datasets or third party data, please ensure that the statement adheres to our policy

The coordinates and cryo-EM density maps generated in this study have been deposited in the Protein Data Bank (PDB) and Electron Microscopy Data Bank (EMDB), respectively. Accession codes are as follows: In situ of the C2S2M2L4-type PSII–LHCII supercomplex (composite): 21WV, EMD-68060; C2S2M2L4-type PSII–LHCII core (protomer 1/2): 9XK3/9XK4, EMD-66946/66947; S-LHCII trimer with CP26 (protomer 1/2): 9XK6/9XK7, EMD-66949/66950; M-LHCII trimer with CP29/CP24 (protomer 1/2): 9XK8/9XK9, EMD-66951/66952; L-LHCII trimers (one side, protomer 1/2): EMD-66971/66974; In situ structure of side-by-side PSII-LHCII dimer (SS dimer): 27UR, EMD-81438; In situ structure of the trans-lumenal PSII-LHCII dimer (TL dimer): 27UT, EMD-81447; In situ structure of the trans-stromal PSII-LHCII dimer (TS dimer): 21WH, EMD-68048; In situ structure of the trans-lumenal/trans-stromal PSII-LHCII trimer (TL-TS trimer): 21WI, EMD-68049; In situ structure of the mixed trans-lumenal/trans-stromal and side-by-side PSII-LHCII tetramer (TL-TS-SS tetramer): 21WG, EMD-68047; In situ structure of the bis-trans-lumenal/trans-stromal PSII-LHCII tetramer (TL-TS-TL tetramer): 21WD, EMD-68043; In situ structure of PSI–LHCI–LHCII supercomplex: 9XJ9, EMD-66932; In situ structure of PSI–LHCI supercomplex: 9XJ1, EMD-66925. All these databases/datasets used in the study along with accession-codes are mentioned in the manuscript under the "Data availability".
All structural models used as initial templates for model building and for comparison with our in situ structures were obtained from the Protein Data Bank (PDB). The following accession codes were used: P. sativum C2S2M2 PSII-LHCII (5XNL), L. oleraceus PSI-LHCI (6YEZ), P. abies C2S2 PSII-LHCII (8C29), C. ohadii PSII (8BD3), and Z. mays PSI-LHCI-LHCII (5ZJI). All are publicly accessible via https://www.rcsb.org and are also mentioned in the manuscript under the "Data availability" as well.

Research involving human participants, their data, or biological material

Policy information about studies with human participants or human data. See also policy information about sex, gender (identity/presentation), and sexual orientation and race, ethnicity and racism.

Reporting on sex and gender	We have not performed research involving human participants.
Reporting on race, ethnicity, or other socially relevant groupings	We have not performed research involving human participants.
Population characteristics	We have not performed research involving human participants.
Recruitment	We have not performed research involving human participants.
Ethics oversight	We have not performed research involving human participants.

Note that full information on the approval of the study protocol must also be provided in the manuscript.

Field-specific reporting

Please select the one below that is the best fit for your research. If you are not sure, read the appropriate sections before making your selection.

☒ Life sciences ☐ Behavioural & social sciences ☐ Ecological, evolutionary & environmental sciences

For a reference copy of the document with all sections, see nature.com/documents/nr-reporting-summary-flat.pdf

Life sciences study design

All studies must disclose on these points even when the disclosure is negative.

Sample size	For cryo-EM data collection, n = 22,785 movies were collected from purified O. sativa chloroplasts. We chose this sample size to achieve local resolutions better than ~2.5 Å for the C2S2M2L4 PSII-LHCII map and better than ~2.0 Å for the PSI-LHCI map. More than half a million high-quality particles were included in the final classification and reconstructions of the C2S2M2L4 PSII-LHCII supercomplex and various types of PSII dimer, trimer, and tetramer supercomplexes. Similarly, more than half a million high-quality particles were used for the final classification and reconstructions of the PSI-LHCI and PSI-LHCI-LHCII supercomplexes.
Data exclusions	All micrographs were manually inspected. Micrographs were excluded based on criteria including excessive drift, unsuitable ice thickness, defocus outside the target range, or poor estimated information content from contrast transfer function (CTF) fitting. Particle projections were subsequently excluded based on poor quality in 2D class averages and outcomes from 3D classification and refinement procedures in CryoSPARC.
Replication	We prepared over twenty cryo-EM grids from multiple batches of naturally grown rice leaves. All grids exhibited consistent imaging quality. Data processing followed a standardized pipeline: micrographs and particle projections were excluded based on objective quality metrics (e.g., ice thickness, drift, defocus, CTF fit, and 2D class appearance). The classified particles were reprocessed to generate reference-free 2D

class averages, which remained consistent across multiple classification cycles, confirming the robustness of our approach. This standardized workflow from sample preparation to structure determination ensured the high replicability of our findings.

Randomization	The particle sets were randomly split into two independent halves to estimate the resolution using the Fourier Shell Correlation (FSC) 0.143 criterion.
Blinding	Blinding was not relevant to this study as no populations were pre-assigned to experimental groups.

Reporting for specific materials, systems and methods

We require information from authors about some types of materials, experimental systems and methods used in many studies. Here, indicate whether each material, system or method listed is relevant to your study. If you are not sure if a list item applies to your research, read the appropriate section before selecting a response.

Materials & experimental systems

n/a	Involved in the study
☒	☐ Antibodies
☒	☐ Eukaryotic cell lines
☒	☐ Palaeontology and archaeology
☒	☐ Animals and other organisms
☒	☐ Clinical data
☒	☐ Dual use research of concern
☐	☒ Plants

Methods

n/a	Involved in the study
☒	☐ ChIP-seq
☒	☐ Flow cytometry
☒	☐ MRI-based neuroimaging

Plants

Seed stocks	The seeds used in this study were of the nationally approved, two-line hybrid indica rice cultivar 'Huiliangyou Yuehe Simiao' (National Variety Approval No. 20206106). Seed stocks were sourced from the certified breeder, ensuring traceability and genetic purity.
Novel plant genotypes	No novel plant genotypes were generated. The material used is a nationally registered and commercially available hybrid rice cultivar.
Authentication	The genetic identity and purity of the plant material are established by its unique National Variety Approval Number (20206106), the paramount government-issued certification for crop cultivars in China. This certification is contingent upon the successful completion of multi-year Distinctness, Uniformity, and Stability (DUS) testing. Furthermore, the focus of this study on the conserved architecture of photosynthetic complexes renders the analysis insensitive to potential minor genetic heterogeneity within a certified pure cultivar.
