# Supplementary material for: In situ structures of plant photosystem supercomplexes
Source: Nature. 2026 Jul 29;657(8133):1094–103. doi: 10.1038/s41586-026-10847-3 (PMC13600941; doi:10.1038/s41586-026-10847-3)
Supplement: Supplementary file 3 [file 41586_2026_10847_MOESM3_ESM.pdf]

Peer Review File

In situ structures of plant photosystem supercomplexes

Corresponding Author: Professor Jiapeng Zhu

Any redactions in this file are there to maintain patient confidentiality, the confidentiality of unpublished data, or to remove third-party material.

Parts of this Peer Review File have been redacted as indicated to remove third-party material.

Attachments originally included by the reviewers as part of their assessment can be found at the end of this file.

Version 0:

Reviewer comments:

Referee #1

(Remarks to the Author)
The manuscript by Li et al describes the in-situ structures of photosystem I and II from higher plants and the ensuing chloroplast architecture. Achieving high resolution structural information from “close to native” state tissues is an exciting development and certainly merits high impact publication. I see two benefits in the work, imaging these systems in their native state and discovering the overall structural motifs of grana membranes.
With regards to imaging the native states of PSI and PSII I have two issues. First is the overall quality of the maps, the authors used standard techniques to estimate the resolution in their reconstructed maps and from the respect I don’t think their claims are exaggerated. However, I do see clear issues in map quality, like noticeable anisotropy and details which according to my experience reflect very noisy data and possibly optical correction issues. This is true even in the focused reconstructed maps which should minimize the effects of heterogeneity. At the very least the authors should acknowledge these issues in the text and provide some reasons. Second, I find the authors molecular modeling contains many errors which should be corrected (detailed below).
With regards to the overall structure of grana stacks, I think the authors addressed some long-standing questions in the field and the findings are exciting and relevant. A technical issue is that typically with large and complicated particles a consensus maps is shown next to the combined focused maps. It’s difficult to follow the authors processing pipeline, but they should address the possibility that these large assemblies contain a patchwork of separated, smaller, PSII assemblies. Another point is the measure of the structural heterogeneity in these large assemblies. I did not notice any attempt to address this using current tools. This issue has implications for both the energy transfer modeling and the overall grana organization. As a general note, the manuscript contains multiple submissions of maps and models. For each figure the pdb/emd codes used in its preparation should be noted or else its impossible to follow the authors work.
Specific comments -
Line 344 – “By contrast, trans-lumenal and trans-stromal dimers adopt precisely regulated “close yet separate” geometries stabilized by complementary electrostatic interactions (Extended Data Fig. 4e,f) and localized hydrogen-bond networks involving PsbO and PsbR.” – looking over Extended Data Fig. 4 I could not see any demonstrated complementarity in electrostatics across the different layers.
Line 356 – “Together, these observations indicate that the stacking interactions do not involve direct contacts between stromal antenna regions but instead involve interactions between PSII–LHCII supercomplex cores.” – The TS dimer has been isolated before with similar conclusions, this work should be cited, and it’s not clear if the dimensions of the current dimer fit with previous findings from plants and green algae.
Data processing – I have commented below on missing details in each of the supplementary images. In addition to those, the authors mentioned global and local ctf refinement, they should specify which parameters were refined in their method section. Many of the maps appear to suffer from some sort of anisotropic distortion which appear to “stretch” the membrane region, the authors should note and address this issue in the text. With regards to PSII oligomers I have two issues. First, it is not possible to determine the differential occupancy in each PSII position from the presented data, this is a critical issue, since when the authors describe what are essentially PSII tetramers at the center of thylakoid architecture, but each of those can potentially be a patchwork of dimers, monomers and trimers. Some of their conclusions may hold, but placing this structure at the center of grana architecture requires a higher level of proof. Second, even if each of the PSII assemblies

described in the authors composite maps represent only complete particles, not enough information is given about the heterogeneity in these particles, from the authors data it's clear that the orientations between each PSII complexes vary over a wide range. I acknowledge that a complete description of this heterogeneity is challenging, but even a rough estimation of these in the particle population will go a long way to improve the work and the picture that emerges of grana architecture. Finally, the same argument holds for the L position LHCII, their location with regards to PSII must be quite variable, the authors should make an effort to describe this and incorporate this data into their modeling.
The authors try and measure the shifts across PSI-LHCI and PSI-LHCI-LHCII, comparing across models and reaching the conclusion that LHCII binding induces shifts in LHCI and PSI core. It's not clear to me why they used 5ZJI here, this comparison includes additional experimental and map quality differences that are difficult to estimate, why not compare the authors two models and see if there is a shift upon LHCII binding.
Molecular modeling – model refinement is unfortunately not up to current standards (pdb validation reports were not provided for review but they cannot be good). The most noticeable measure is the high clash score of all the models. A badly refined model into a 3 Å map should have a clashscore of about 10 using modern tools, the authors models score close to 40, this suggests an underlying issue with refinement. Going over the PSI-LHCI model, I encountered multiple issues. Lipid placement should be a lot more conservative, especially when this is one of the new features of the work. If only the head group and a few atoms of the fatty acids are visible, the lipid should be truncated to fit the map. I think the authors might have turned on automatic linking in phenix.refine which can also cause issues in refinement of pigment protein complexes. Some lipids are placed in a way which I can only described as fitting noise (the same can be said on the N terminus of chain 1v in their PSII model) and those should be completely removed from the models.
Lipid identification, especially in the case of SDQ, most of the sulfur atoms do not seem to be covered by the map at any level (I looked at EMD-68060 which I think offered the best map for lipids and Q). Same is true for many LHG's, since this is a native sample, I would expect more MGDG and DGDG. In addition, the PSI-LHCI-LHCII contains one detergent molecule…… Many of the fatty acids are overbuilt and should be truncated. The same is true for Qc. If the authors think this site exist, they should provide map samples for this site which clearly shows quinone density next to additional protein features. For Qd, there is clear density, but how did the authors assign a quinone to this density? They should clearly describe their justification. In addition, it seems that not enough manual fitting was carried out, and some side chains are found in orientations which are clearly wrong, while this is acceptable in some map regions, it is not acceptable in map regions which are considered to be very good, such as the PSI core. I would suggest that the authors will refine high quality models into their composite maps of PSI and PSII and use less detailed models docked into densities for their larger scale maps of PSII. This is at their discretion, but the model should reflect the level of details found in the map, and so, side chains, chlorophyll tails and lipids should be removed whenever not visible, regardless of the reported resolution.
Line 114 – "extrinsic subunit PsbQ but contains PsbTn2" what does PsbTn2 represents ?
Page 3 of Supplementary Information "This process involved successive rounds of 2D particle picking"
Right after "LHCII – PsaO" in Supplementary Information - "The rate of excitation energy between"
Supplementary Fig. 2 – The micrograph image should be supplied as a size and resolution that will allow the reader to visualize at least some particles. Class distributions should be shown in part 'd' to assess how common this arrangement is. The authors must show that they have excluded most PSII monomers from each side of the SS dimer using classification/focused classification. The tool/procedure to construct the composite map is not described.
Supplementary Fig. 3 – The micrograph image should be supplied as a size and resolution that will allow the reader to visualize at least some particles. Class distribution and particle numbers should be shown for part 'd' (possibly only the bottom part) and 'g'. The final number of particles in the dimeric data set should be noted. The tool/procedure to construct the composite map is not described.
Supplementary Fig. 4 – The micrograph image should be supplied as a size and resolution that will allow the reader to visualize at least some particles. It is very difficult to follow the authors pipeline for this map, I suggest that they will assign distinct colors for each PSII level, that might help. What should be presented very clearly (and is currently unknown) is how many particles contain all three PSII complexes and what is the overlap between sets 1, 2 and 3. Whenever the authors classify their dataset, they should give a measure of the proportion of each class in the total population and specify the number of particles in their final dataset.
Supplementary Fig. 5 and 6 - The micrograph image should be supplied as a size and resolution that will allow the reader to visualize at least some particles. The same comments that were mentioned for S. fig. 4 apply. The authors should provide total number of particles whenever a classification step is carried out, and a clear description of how many particles contained the four PSII complexes in the final composite map, as well as how many complexes are present in each distinct location based on classification or another measure.
Supplementary Fig. 7 - The micrograph image should be supplied as a size and resolution that will allow the reader to visualize at least some particles.

Referee #2

(Remarks to the Author)
This is an exciting, impactful, and technically impressive paper that resolves several long-standing questions in the field of photosynthesis particularly around chloroplast architecture. The use of in situ cryo-EM structural analysis has revealed several aspects of chloroplast organization – including resolving the protein contacts responsible for grana stacking, curvature of the membrane, quinone positions, and in vivo stoichiometry of the supercomplexes. These are important contributions and I recommend that the manuscript be accepted for publication.

The excitation energy calculations have the potential to connect these structural findings to functional aspects of light harvesting, increasing the impact of the work. However, in their current form, these calculations are primarily a control

confirming agreement between the supercomplex structures reported here and previously work on in vitro supercomplexes, and so would be more appropriate for the supplementary information. The authors should clarify the insights from energy transfer calculations of the structures through improvements to the methodology and analysis.

1. Side-by side dimer: the authors conclude energy transfer between PSII supercomplexes in the side-by-side structure is unlikely. However, they used a simplified FRET model in which the energies of all the chlorophyll a (or chlorophyll b) are equivalent. In such a model, photoenergy does not localize due to lower energy sites, and so the energy transfer pathways can change with the flatter energetic landscape. It is for this reason that more sophisticated methods have been developed and are now typically used in the field (such as the one the authors themselves used for the PSII supercomplexes). Thus, while I agree that energy transfer between side-by-side dimers may be relatively unlikely, the conclusion is not well justified without a more realistic energy landscape.
2. Trans-stromal dimer: the authors conclude there is no energy transfer between dimers owing to a distance between chlorophylls of ~5 nm. However, the authors also state (173ff): “All types of dimers show substantial variability in angles and distances…” What is the magnitude of this variability? A small change might bring the chlorophylls close enough to enable energy transfer. Can the authors recalculate the rates across the range of observed variability?
3. The authors state: “The weak connection of L-LHCII may facilitate their dynamic association with the C2S2M2 complex, facilitating antenna size regulation, albeit at the cost of a reduced energy transfer efficiency.” The authors should better justify this statement – where would the isolated LHCII go? How is it dynamically associated?
4. The authors calculate the predicted lifetimes for supercomplexes of different sizes, which is compared to other measurements in Fig 5b. Can the authors further analyze this data to make any conclusions about the likely size of supercomplexes in other species/growth conditions?

Referee #3

(Remarks to the Author)
This manuscript describes an in situ structural analysis of PSII and PSI from chloroplast of O. sativa by cro-EM. It was found that PSII-LHCII exists not only in a C2S2M2L4 state, but also organizes into three distinct higher ordered forms of (1) side-by-side dimer; (2) trans-lumenal (TL) dimer; and (3) trans-stromal (TS) dimer. In addition, both PSI-LHCI and PSI-LHCI-LHCII forms are found in the native membrane. While some of these results are new and worth publication, I have the following concerns that need the authors to consider.

Major points
The authors showed that the membrane stacking is due to TL and TS dimer connections, which are mediated by PsbO and PsbR subunits, respectively, both are PSII core subunits. This shows that the membrane stacking is not related with LHCII proteins. However, there are many previous studies indicating that lacking of LHCII subunits (either by deleting LHCII genes or Chl b synthesizing genes) led to unstacking of the membrane in higher plants. How do the authors explain these previous results?

It is known that electron transfer goes through PSII → cytochrome b6/f → PSI. The authors showed the PSII and PSI structures separately, but do not show their connectivity nor the structure of cytochrome b6/f complex. If the membrane is intact, as suggested by the authors (because they are using intact chloroplast), it is expected to see the connectivity of PSII and PSI, as well as the cytochrome b6/f complex. Can the authors discuss on this point?

Page 9, line 179-, and Fig. 4d: It is stated that “Our high-resolution structures reveal that PSII-LHCII supercomplexes assemble into extensive, higher-order structures that form a PSII-LHCII-based stacking network within the thylakoid membranes”. However, there are no evidence showing the higher-order structures of the membrane. Fig. 4d is only a model of the membrane. Please clarify and comment on this.

Minor points
A C2S2M2L2 form of PSII-LHCII has previously been observed in green algae; this should be mentioned and cited (Sheng X. et al., Nat Plants. 2019; Suga M. et al., Nat Plants. 2019; Shen L. et al., Proc Natl Acad Sci U S A. 2019).

Lines 49: “…photosynthetic bacteria (10-14)”. Refs. 10, 11, 12 and 14 cited here are not related with cryo-EM structures of photosynthetic bacteria, instead, two of them are XFEL structural studies of PSII from cyanobacteria, and two are cryo-EM structural studies of cyanobacteria. There are a number of cryo-EM studies regarding the photosynthetic bacterial photosystems, for example:

Qian P, Siebert CA, Wang P, Canniffe DP, Hunter CN., Cryo-EM structure of the Blastochloris viridis LH1-RC complex at 2.9 A. Nature. 2018 Apr;556(7700):203-208. doi: 10.1038/s41586-018-0014-5.

Dong S, Huang G, Wang C, Wang J, Sui SF, Qin X., Structure of the Acidobacteria homodimeric reaction center bound with cytochrome c. Nat Commun. 2022 Dec 14;13(1):7745. doi: 10.1038/s41467-022-35460-6.

Fig. 1, In the C2S2M2L2 structure, the L-trimer seems to have no direct interactions with other trimers. How do they bind specifically? In panel k: It should be “PSI core”, not “PSII core”. Panels h and l could be moved to Extended Data Figs. to reduce the space for the main text.

Legend to Fig. 2: “…with indicated the subunits of the antenna”. Need to rephrase. Not only the antenna subunits but also

the core subunits are labeled in the figure.

Fig. 3f, i: The interactions between neighboring complexes are shown, but the resolution of the maps are low and not sufficient to identify these interactions. How the authors assigned these interactions?

From the bottom of page 5 to the beginning of page 6: The assignment of four PQ9 was described here and refers to Fig. 9, but PQ9 was not clearly visible in Fig. 9.

Supplementary Information, page 2, lines 7-8: “In our structure reveals…”. Need rephrase.

Page 8, line 145: “apposition” → “association”?

Page 9, lines 165-166: “Extended Data Fig. 5f” → may be “Extended Data Fig. 4f”? In Extended Data Fig. 4f, a PsbV subunit is labeled, but this is a subunit found in cyanobacterial PSII and not present in higher plants. Please clarify.

Page 13, line 259: “…five β-carotenes, five Chl a molecules…”. In Extended Data Fig. 6a, b, 4 β-carotenes and five Chl a molecules were shown, but in the legend, it says “4 β-carotenes, 4 Chl a molecules”. Please clarify.

Version 1:

Reviewer comments:

Referee #1

(Remarks to the Author)
The authors have answered my critique regarding the possibility of their larger assemblies being composed of a mosaic of partial assemblies. In my opinion, the use of “high resolution” in the title is unjustified and should be replaced with something that fits the findings of the paper better.
Minor issues - The quality of the modeling is significantly improved. Given the claim for an in-vivo structure, I still find issues with some of the lipid assignments (for example, most/all the SDQ assignments in their PSII model are not supported by the data).
given that these issues are resolved i support the publication of the manuscript.

(Remarks on code availability)

Referee #2

(Remarks to the Author)
The authors have adequately addressed my concerns.

(Remarks on code availability)

Referee #3

(Remarks to the Author)
The authors have addressed my concerns adeauately, and I have no further questions.

(Remarks on code availability)

Open Access This Peer Review File is licensed under a Creative Commons Attribution 4.0 International License, which permits use, sharing, adaptation, distribution and reproduction in any medium or format, as long as you give appropriate credit to the original author(s) and the source, provide a link to the Creative Commons license, and indicate if changes were made.
In cases where reviewers are anonymous, credit should be given to 'Anonymous Referee' and the source.
The images or other third party material in this Peer Review File are included in the article's Creative Commons license, unless indicated otherwise in a credit line to the material. If material is not included in the article's Creative Commons license and your intended use is not permitted by statutory regulation or exceeds the permitted use, you will need to obtain permission directly from the copyright holder.
To view a copy of this license, visit https://creativecommons.org/licenses/by/4.0/

Referee #1 (Remarks to the Author):

The manuscript by Li et al describes the in-situ structures of photosystem I and II from higher plants and the ensuing chloroplast architecture. Achieving high resolution structural information from "close to native" state tissues is an exciting development and certainly merits high impact publication. I see two benefits in the work, imaging these systems in their native state and discovering the overall structural motifs of grana membranes.

With regards to imaging the native states of PSI and PSII I have two issues. First is the overall quality of the maps, the authors used standard techniques to estimate the resolution in their reconstructed maps and from the respect I don't think their claims are exaggerated. However, I do see clear issues in map quality, like noticeable anisotropy and details which according to my experience reflect very noisy data and possibly optical correction issues. This is true even in the focused reconstructed maps which should minimize the effects of heterogeneity. At the very least the authors should acknowledge these issues in the text and provide some reasons. Second, I find the authors molecular modeling contains many errors which should be corrected (detailed below).

1. Regarding the map quality:

We thank the reviewer for this insightful observation regarding the map quality. We agree with the assessment that our reconstructions, specifically for the PSII-LHCII supercomplexes, exhibit anisotropy and local noise, resulting in local map qualities lower than the nominal gold-standard FSC (FSC=0.143) estimates might suggest. We attribute these challenges to the inherent limitations of sample preparation and cryo-EM imaging within the native thylakoid environment, as described below:

***Preferred orientation and sample geometry*:** PSII is predominantly localized within the tightly appressed, planar regions of the grana. This rigid, parallel membrane

architecture within our chloroplast samples imposes a significant constraint on the Euler angle distribution, as particles are predominantly "locked" in a fixed orientation relative to the membrane normal. Furthermore, during cryo-EM grid preparation, the chloroplasts are likely constrained by the thin aqueous layer within the grid holes. These cause the grana stacks to preferentially orient parallel to the grid surface, leading to sampling bias and the observed anisotropy in the final reconstruction.

Background noise**:** The vertical resolution primarily depends on side views, which are quite thick due to the special organization of grana membrane structures and stacking of multiple, protein-dense layers. As a result, images from those side views display a higher background noise level (lower SNR) compared to top views, thus impairing the final reconstruction along the vertical axis.

Conformational heterogeneity**:** The PSII-LHCII supercomplex is known for its structural flexibility, particularly involving the peripheral antenna proteins. This inherent heterogeneity likely contributes to the local density blurring observed in the distal regions of the map, even after focused refinement. By contrast, the core region of this supercomplex was well-resolved.

To minimize these issues during data processing, the results presented in our primary manuscript were obtained after performing extensive 3D classification on the initially acquired data to enrich for homogeneous particle populations. Furthermore, we utilized the “Rebalance Orientations” tool in CryoSPARC to mitigate the over-contribution of preferred orientation particles and achieve a more isotropic sampling of the final map.

Following the reviewer's suggestion, we have acknowledged these limitations and provided potential explanations in the second paragraph of the "Initial 3D Reconstruction of PSI-LHCI and PSII-LHCII" subsection in the Supplementary Information Methods. We have also described strategies to minimize these issues during data processing. Please refer to the Supplementary Information, page 5, lines 102–106.

2. Regarding the molecular modeling errors:

We highly appreciate the reviewer's suggestion. The specific errors in the PDB models have been corrected. The revised models have been uploaded to the Sharepoint folder named "Revised models and TL dimer map". The coordinates are updated in the Protein Data Bank (PDB) upon resubmission.

With regards to the overall structure of grana stacks, I think the authors addressed some long-standing questions in the field and the findings are exciting and relevant. A technical issue is that typically with large and complicated particles a consensus maps is shown next to the combined focused maps. It's difficult to follow the authors processing pipeline, but they should address the possibility that these large assemblies contain a patchwork of separated, smaller, PSII assemblies. Another point is the measure of the structural heterogeneity in these large assemblies. I did not notice any attempt to address this using current tools. This issue has implications for both the energy transfer modeling and the overall grana organization.

We thank the reviewer for this comment. We address each point below.

1. Regarding the processing pipeline and the display of consensus maps:

We have revised the presentation of the processing workflows for PSII dimer, trimer and tetramer structures in Supplementary Figs. 2–6. To facilitate tracking through the classification and reconstruction steps, the PSII units that form these structures are labeled according to their oligomeric state: for dimers, $C_2S_2M_2$ (black) and $C_2'S_2'M_2'$ (blue); for trimers, $C_2S_2M_2$ (black), $C_2'S_2'M_2'$ (blue) and $C_2''S_2''M_2''$ (magenta); and for tetramers, all four labels (including $C_2'''S_2'''M_2'''$ in orange), These labels are also indicated adjacent to the corresponding densities in the final composite map to help readers follow the processing workflow.

In addition, the consensus maps of the side-by-side (SS), trans-lumenal (TL) and trans-stromal (TS) PSII-LHCII dimer are now shown in Supplementary Figs. 2l, 3j and 4j,

respectively, and are explicitly labeled beneath the corresponding panels. These maps serve as reference frameworks for the composite maps generated after focused refinements.

For the process for generating composite maps of higher-order macroscopic stacking assemblies (TL-TS trimers, TL-TS-SS, and TL-TS-TL tetramers) presented in Supplementary Figs. 4q–y, 5, and 6, signal subtraction was applied to remove interference from adjacent complexes in non-focused regions, while masking was employed for the focused components to enhance local resolution. The focused-refined components were multiplied with the mask to remove densities outside the masked region, then aligned back to the original consensus maps to preserve the spatial relationships between PSII units and obtain a higher-resolution composite map. We have added previously omitted consensus maps in Supplementary Figs. 4q, 5c and 6b. Each PSII assembly is colour-coded and labelled to facilitate following the workflow. These assemblies are exceptionally large, and their consensus maps are of limited quality; focused refinements improved the nominal resolution and map quality as well.

Please refer to the revised Supplementary Figs. 2–6 and their corresponding legends in the Supplementary Information.

2. Regarding the possibility of a "patchwork of separated, smaller PSII assemblies":

The reviewer raises an important point. We minimized this possibility through the following process. First, we performed independent 3D classification on each constituent of the assembly. This consistently yielded a major class with improved resolution compared to the initial unclassified consensus map, suggesting enrichment for a discrete, homogeneous population. To further ensure compositional integrity, we used metadata intersection (particle UIDs) to select only those particles that belonged to the high-resolution, structurally complete classes for every constituent simultaneously. This helps filter out partial or fragmented assemblies. Following the

reviewer's suggestion, we have added a description of this approach in the fourth paragraph of the "Cross-classification of multiple complexes" subsection in the Supplementary Information Methods.

3. Regarding the measurement of structural heterogeneity:

To measure the relative movement of individual constituents, we employed a hierarchical refinement approach for the dimeric assemblies. First, a consensus local refinement was performed using a mask covering the entire dimer, with the alignment resolution limited to 4.5 Å. This strategy prevents the alignment from being dominated by one side which achieves high resolution first and then dominates the alignment; by limiting the resolution, we ensure a balanced global alignment for both sides of the dimers based on the overall assembly architecture. Subsequently, independent focused refinements were performed for each constituent PSII-LHCII supercomplex using localized masks to capture their specific, high-resolution orientations.

By extracting particle metadata (Euler angles and translation vectors) from both refinement stages, we quantified the rotational and translational variance of individual PSII monomers at the particle level. Our measurements suggest that each constituent can be described as wobbling and shearing within a defined "conformational envelope". The detailed method is described in the newly added "Quantification of structural heterogeneity of PSII dimers" subsection in the Supplementary Information Methods, and the results are presented in new Supplementary Fig. 12. All codes used to quantify structural heterogeneity of PSII dimers (analyze_refine.py and calcu_angles.py) are available at https://github.com/JackZhang-Lab/EM-scripts. Please refer to the revised Supplementary Information Methods.

As a general note, the manuscript contains multiple submissions of maps and models. For each figure the pdb/emd codes used in its preparation should be noted or else its impossible to follow the authors work.

We agree that clearly linking each figure to the corresponding structural data is essential for readers to follow our work. Accordingly, we have carefully reviewed all figures in the main manuscript and the Supplementary Information and have added the relevant PDB and EMDB accession codes to each figure legend where applicable. All modifications have been incorporated into the revised manuscript.

Specific comments –

Line 344 – "By contrast, trans-lumenal and trans-stromal dimers adopt precisely regulated "close yet separate" geometries stabilized by complementary electrostatic interactions (Extended Data Fig. 4e,f) and localized hydrogen-bond networks involving PsbO and PsbR." – looking over Extended Data Fig. 4 I could not see any demonstrated complementarity in electrostatics across the different layers.

Upon re-examination of the trans-stromal and trans-lumenal dimer interfaces (Extended Data Fig. 4e,f and new Supplementary Fig. 15), the stromal and lumenal surfaces of PSII contain both positively and negatively charged residues that are likely to form electrostatic interactions when they come into close proximity. We propose that the electrostatic interaction and hydrogen-bond networks involve distinct proteins, as shown in the corresponding main text figures: PsbO (Fig. 3f) and PsbR (Fig. 3i). We have now corrected the text accordingly, and the revised sentence is as follows:

"By contrast, trans-lumenal and trans-stromal dimers adopt precisely regulated "close yet separate" geometries stabilized by electrostatic interaction and localized hydrogen-bond networks involving PsbO, CP43 and PsbR (Fig. 3f,i, Extended Data Fig. 4e,f, Supplementary Fig. 15)."

Line 356 – "Together, these observations indicate that the stacking interactions do not involve direct contacts between stromal antenna regions but instead involve interactions between PSII–LHCII supercomplex cores." – The TS dimer has been isolated before with similar conclusions, this work should be cited, and it's not clear if the dimensions

of the current dimer fit with previous findings from plants and green algae.

1. Regarding citations of previous work on TS dimers:

As the reviewer noted, we have indeed cited the relevant studies that reported TS dimers and proposed models for grana stacking. At the beginning of this paragraph, we cited two key papers: Albanese *et al.*, *Nat Commun* 2020, 11, 1361, and Albanese *et al.*, *Sci Rep* 2017, 7, 10067. These are all from purified complexes and their conclusions are that the stacking is mediated by the antennae (Albanese *et al.* 2017, but very low resolution; Albanese *et al.* 2020, which is mainly cross-linking MS; and Caspy *et al.*, *eLife* 2023, 12, e81150 for *Dunaliella*, in which they do not observe direct interactions). However, these conclusions are not the same as those indicated here.

These studies proposed that LHCII and other antenna components (e.g., CP29) play central roles in the molecular basis of grana stacking. However, the in situ structures reported in this study provide direct visualization of the trans-stromal interactions and reveal a different picture. Specifically, we observe no direct interactions across the stromal gap involving LHCII or CP29. This conclusion is supported by the following key observations from our structural analysis: As shown in Fig. 3g-i, Extended Data Fig. 4f and Supplementary Fig. 15c,d, our result regarding the interaction mediated by PsbR, which forms a stromal gap of approximately 3 nm, is consistent with the cryo-ET data (reference 31 in the main text). Additionally, the antenna components all have small extra-membrane parts, and if stacking were mediated by the antennae, the stromal gap would be too small.

Importantly, our findings are consistent with and provide molecular model validation for the recent cross-linking experiments by Shan et al. (*Sci Adv* 2024, 10, eadq9967; reference 32 in the main text). This study demonstrated that the stromal-side interactions observed involve the stromal loop of the PsbR subunit and are compatible with cross-linking experiments performed in native membranes. We have cited this work in the relevant context with the statement: "In contrast, the stromal-side

interactions observed here involve the stromal loop of the PsbR subunit and are compatible with cross-linking experiments performed in native membranes[32]" to support our conclusion.

2. Regarding comparison of dimer dimensions with previous findings:

As discussed above, the stromal gap would be too small if stacking were mediated by antenna proteins; therefore, the dimensions of the current dimer would not fit previous findings.

Data processing – I have commented below on missing details in each of the supplementary images. In addition to those, the authors mentioned global and local ctf refinement, they should specify which parameters were refined in their method section. Many of the maps appear to suffer from some sort of anisotropic distortion which appear to "stretch" the membrane region, the authors should note and address this issue in the text. With regards to PSII oligomers I have two issues. First, it is not possible to determine the differential occupancy in each PSII position from the presented data, this is a critical issue, since when the authors describe what are essentially PSII tetramers at the center of thylakoid architecture, but each of those can potentially be a patchwork of dimers, monomers and trimers. Some of their conclusions may hold, but placing this structure at the center of grana architecture requires a higher level of proof. Second, even if each of the PSII assemblies described in the authors composite maps represent only complete particles, not enough information is given about the heterogeneity in these particles, from the authors data it's clear that the orientations between each PSII complexes vary over a wide range. I acknowledge that a complete description of this heterogeneity is challenging, but even a rough estimation of these in the particle population will go a long way to improve the work and the picture that emerges of grana architecture. Finally, the same argument holds for the L position LHCII, their location with regards to PSII must be quite variable, the authors should make an effort to describe this and incorporate this data into their modeling.

1. Regarding the missing details in each of the supplementary images:

We thank the reviewer for the detailed comments. We have made corresponding revisions in the manuscript and provided responses in the relevant comments below.

2. Regarding the CTF refinement parameters:

We agree that the specific parameters refined during global and local CTF refinement should be clearly stated. In our workflow, global and local CTF refinement were performed using the default parameter settings in cryoSPARC. Global CTF refinement refined defocus (value and astigmatism), phase shift, tilt, and trefoil over one iteration, with a minimum fit resolution of 10 Å. Local CTF refinement additionally refined per-particle defocus with a minimum fit resolution of 20 Å and a defocus search range of ±2000 Å. Spherical aberration, tetrafoil, anisotropic magnification, and EWS curvature were not refined during either step.

We have now added this clarification to the end of the first paragraph of the "Initial 3D Reconstruction of PSI-LHCI and PSII-LHCII" subsection in the revised Methods section (please refer to the Supplementary Information, page 5, lines 89–90).

3. Regarding the anisotropic distortion ("stretching" of membrane regions):

We acknowledge the presence of anisotropic distortion in membrane regions of our maps, particularly for the higher-order PSII assemblies. These likely arise from preferential particle orientation inherent to the in situ sample preparation, relative movements among different components, and intrinsic membrane flexibility (as discussed in our response to the reviewer's earlier comment). In the revised manuscript, we have added a discussion of this limitation in the Discussion section (main text page 19, lines 373–380). Additionally, we have described the anisotropic correction applied during reconstruction in the second paragraph of the "Initial 3D Reconstruction of PSI-LHCI and PSII-LHCII" subsection in the Supplementary Information Methods.

4. Regarding differential occupancy and the 'patchwork' concern for PSII oligomers:

We thank the reviewer for raising this important point. To address the possibility that higher-order PSII assemblies (including the PSII tetramers mentioned by the reviewer) might represent a patchwork of smaller, independent units rather than fully occupied complexes, we performed additional focused 3D classifications on each PSII position within these assemblies, as now described in the fourth paragraph of the "Cross-classification of multiple complexes" subsection in the Supplementary Information Methods. For each assembly type, soft masks were generated around individual PSII positions and 3D classification without alignment was performed to assess occupancy independently. The process minimized the possibility that the particles used for the final reconstructions were derived from smaller or incompletely occupied assemblies. These occupancy analysis results have been added as Supplementary Figs. 2j, 2k, 3h, 4h, 4i, 4s, 5f, 5j,5m, 6h-j.

We note that our extensive 3D classification steps likely selected for fully occupied assemblies, whereas the conclusions regarding the tetrameric architecture apply only to the subset of particles that yielded well-behaved reconstructions and may not necessarily represent a configuration universally present between grana thylakoid membranes.

5. Regarding the heterogeneity in PSII assemblies and its measurement:

We thank the reviewer for this important comment. This point has been addressed in our response to the earlier comment on the measurement of structural heterogeneity (please see above for details). The method is also described in the "Quantification of structural heterogeneity of PSII dimers" subsection of the Supplementary Information Methods, and the results are presented in new Supplementary Fig. 12.

6. Regarding the L position LHCII:

As noted by the reviewer, the LHCII trimers at the L position exhibit substantial flexibility, as reflected by the weaker density and lower local resolution in these regions relative to the PSII core, S-LCHII and M-LHCII regions. In the revised Discussion, we have added a description of this inherent flexibility (main text page 17, lines 333–343), noting that these peripheral antenna subunits are not rigidly fixed, and we discuss potential contributing factors, including: under physiological conditions, L-LHCII may be inherently dynamic, participating in state transitions or light adaptation processes to regulate excitation energy distribution. Although an average position for these LHCII trimers was defined from the composite maps, their precise orientations and distances are expected to vary in the native membrane. Under the in situ sample conditions employed in this study, a detailed characterization of the conformational heterogeneity of these peripheral subunits is inherently limited—their association is relatively weak and highly dynamic, making it difficult to capture a stable, single conformation during cryo-sample preparation and image processing. Forcing classification or refinement under such conditions could lead to overfitting or misleading results. Therefore, such detailed characterization falls beyond the scope of the current study. This limitation is now acknowledged in the first paragraph of the revised Discussion section. However, the calculated lifetime of in particular the $C_2S_2M_2L_4$ particle matches well with the measured lifetime in intact membranes, which indicates that although some heterogeneity can be expected, this organization is representative of what is present in the membrane.

The authors try and measure the shifts across PSI-LHCI and PSI-LHCI-LHCII, comparing across models and reaching the conclusion that LHCII binding induces shifts in LHCI and PSI core. It's not clear to me why they used 5ZJI here, this comparison includes additional experimental and map quality differences that are difficult to estimate, why not compare the authors two models and see if there is a shift upon LHCII binding.

1. Regarding the use of 5ZJI as a reference:

The reviewer raises a valid point about why we included the *Z. mays* PSI-LHCI-LHCII structure (PDB 5ZJI) in our comparison. Our rationale is as follows: the 5ZJI structure currently represents the largest known complex formed between PSI and its peripheral antennae in higher plants. We employed it as a reference specifically to highlight the differences between our in situ structures and purified, detergent-solubilized counterparts. As shown in Fig. 6h–i and Extended Data Fig. 6, these comparisons reveal substantial differences in both composition (e.g., additional cofactors preserved in our in situ structures) and local subunit conformations, which we attribute to the more native lipid environment maintained in our preparation. The 5ZJI comparison was therefore intended to illustrate the effects of biochemical isolation—not to serve as the primary evidence for LHCII-induced conformational changes.

2. Regarding the direct comparison of our two in situ PSI models:

We have indeed performed a direct comparison between our two in situ structures—PSI-LHCI and PSI-LHCI-LHCII—to assess the specific effects of LHCII binding. These results are presented in Fig. 6e–g, where we show that LHCII association induces asymmetric dynamic movements of the LHCI belt and peripheral subunits. This direct comparison, derived from structures determined within the same study under consistent experimental conditions, provides the most rigorous and controlled assessment of LHCII-induced conformational changes.

In summary, the two comparisons serve complementary purposes:

The PSI-LHCI versus PSI-LHCI-LHCII comparison (Fig. 6e–g) directly addresses LHCII-induced conformational changes, as suggested by the reviewer.

The in situ PSI-LHCI-LHCI versus 5ZJI comparison (Fig. 6h–i, Extended Data Fig. 6) highlights the differences between native and purified states, underscoring the value of in situ structural determination.

We hope this clarifies our rationale and confirms that both requested comparisons are

already present in the manuscript. We thank the reviewer for the opportunity to elaborate on this point.

Molecular modeling – model refinement is unfortunately not up to current standards (pdb validation reports were not provided for review but they cannot be good). The most noticeable measure is the high clash score of all the models. A badly refined model into a 3 Å map should have a clashscore of about 10 using modern tools, the authors models score close to 40, this suggests an underlying issue with refinement. Going over the PSI-LHCI model, I encountered multiple issues. Lipid placement should be a lot more conservative, especially when this is one of the new features of the work. If only the head group and a few atoms of the fatty acids are visible, the lipid should be truncated to fit the map. I think the authors might have turned on automatic linking in phenix.refine which can also cause issues in refinement of pigment protein complexes. Some lipids are placed in a way which I can only described as fitting noise (the same can be said on the N terminus of chain 1v in their PSII model) and those should be completely removed from the models.

Lipid identification, especially in the case of SDQ, most of the sulfur atoms do not seem to be covered by the map at any level (I looked at EMD-68060 which I think offered the best map for lipids and Q). Same is true for many LHG's, since this is a native sample, I would expect more MGDG and DGDG. In addition, the PSI-LHCI-LHCII contains one detergent molecule…… Many of the fatty acids are overbuilt and should be truncated. The same is true for Qc. If the authors think this site exist, they should provide map samples for this site which clearly shows quinone density next to additional protein features. For Qd, there is clear density, but how did the authors assign a quinone to this density? They should clearly describe their justification. In addition, it seems that not enough manual fitting was carried out, and some side chains are found in orientations which are clearly wrong, while this is acceptable in some map regions, it is not acceptable in map regions which are considered to be very good, such as the PSI core. I would suggest that the authors will refine high quality models into their

composite maps of PSI and PSII and use less detailed models docked into densities for their larger scale maps of PSII. This is at their discretion, but the model should reflect the level of details found in the map, and so, side chains, chlorophyll tails and lipids should be removed whenever not visible, regardless of the reported resolution.

We sincerely thank the reviewer for the meticulous and professional review of our modeling and refinement. We have thoroughly revised all models in response.

We agree that the clash scores in our original submission were higher than desirable. Following the reviewer's suggestions, we carefully re-examined the fit of the $C_2S_2M_2$ region model against the corresponding map, addressing side chains, chlorophyll tails, lipids, Q_C, lipid-type assignments and the N-terminus of chain 1v. We similarly re-examined the PSII-LHCI and PSI-LHCI models against their respective maps, including the identification and correction of a detergent molecule (LMU) in the PSI-LHCI-LHCII model noted by the reviewer. All incorrectly placed side chains have been manually adjusted. Chlorophyll tails have been truncated where density is not visible. Lipids have been truncated or removed where density is not visible, and several LHGs have been re-assigned as MGDG (MGE in the model). We then performed multiple rounds of manual adjustment and correction in Coot guided by the bad clashes information from the refinement results, and removed side chains at sites with very poor fit. The revised PSII and PSI models show substantially improved clash scores (see updated Supplementary Tables 3–6).

Regarding Q_C in PSII, weak long tail density is observable on one side, whereas the head region is more clearly defined on the other, indicating substantial local flexibility or lower occupancy. We have now addressed this point in the main text (page 7, lines 134–135). We have retained the density-supported region in the model.
Please refer to the newly uploaded "6_Revised_800_pixel_box_reconstruction_C2S2M2L4_21WV.pdb" for its fit with the corresponding map "4_Composite_800_pixel_box_reconstruction_C2S2M2L4_EMD-68060.mrc".

For Q_D, we have clarified in the main text (page 7, lines 137–139) that its assignment as a plastoquinone is based on its membrane-spanning length, lack of polypeptide-chain characteristics and excellent fit of a plastoquinone model into the corresponding density.

The higher-order PSII assemblies (dimers, trimers and tetramers) were obtained by fitting the refined $C_2S_2M_2$ models, with side chains and ligands removed, into the densities of individual PSII units. For the L4 region of the $C_2S_2M_2L_4$ model, we used the LHCII model from PSI-LHC-LHCII, with side chains and ligands removed, to fit the distinct triangular densities. These procedures are now described in the "Model building, refinement, and validation" subsection of the Supplementary Information Methods.

The revised models have been uploaded to the Sharepoint folder named "Revised models and TL dimer map". All supporting validation statistics are provided in Supplementary Tables 3–6.

Line 114 – "extrinsic subunit PsbQ but contains PsbTn2" what does PsbTn2 represents?

PsbTn is a well-characterized extrinsic subunit of PSII. Previous structural studies have reported variability in the presence of the extrinsic subunits PsbQ and PsbTn across different plant species. For example, Wei et al. (*Nature* 2016, 534, 69-74) identified both PsbTn and PsbQ in spinach C_2S_2, and Su et al. (*Science* 2017, 357, 815-820) reported both subunits in *Pisum sativum* $C_2S_2M_2$. By contrast, Opatíková et al. (*Nat Plants* 2023, 9, 1359-1369) observed PsbTn but not PsbQ in spruce C_2S_2. Our in situ structure of *O. sativa* $C_2S_2M_2L_4$ similarly contains PsbTn but lacks PsbQ, consistent with the findings in spruce. Reference 2, cited next to "PsbTn", corresponds to Opatíková et al. (*Nat Plants* 2023, 9, 1359-1369) and supports our observation. The absence of PsbQ may reflect species-specific differences or physiological conditions, and further investigation will be needed to clarify the factors influencing its retention in PSII supercomplexes.

Page 3 of Supplementary Information "This process involved successive rounds of 2D particle picking"

We thank the reviewer for the careful reading. The phrase has been corrected to "successive rounds of particle picking" in the revised Supplementary Information to accurately reflect the iterative particle selection workflow. This correction has been made in the updated version.

Right after "LHCII – PsaO" in Supplementary Information - "The rate of excitation energy between"

We thank the reviewer for pointing out this grammatical issue. The phrase has been corrected to "the rate of excitation energy transfer between" in the revised Supplementary Information to accurately describe the energy transfer process. This correction has been made in the updated version.

Supplementary Fig. 2 – The micrograph image should be supplied as a size and resolution that will allow the reader to visualize at least some particles. Class distributions should be shown in part 'd' to assess how common this arrangement is. The authors must show that they have excluded most PSII monomers from each side of the SS dimer using classification/focused classification. The tool/procedure to construct the composite map is not described.

We have revised the figure and the corresponding text as follows：

1. Regarding the micrograph image:

We agree that a clearer micrograph is necessary. The original micrograph in Supplementary Fig. 2c has been replaced with a larger, higher-resolution version. Please note that the image, although of high resolution in the original Word file, is subject to clarity loss upon PDF conversion.

2. Regarding the class distributions:

As suggested, we have now included in Supplementary Fig. 2d the percentage of particles contributing to each major 3D class average. Among the classified populations, the side-by-side PSII arrangement represents the largest class, accounting for approximately 33% of the selected particles.

3. Regarding the exclusion of PSII monomers from each side of the SS dimer:

To demonstrate that most PSII monomers were excluded, we performed focused classification on each side of the SS dimer using separate masks. The resulting class distributions are now presented in the updated Supplementary Fig. 2j, k. In addition, a detailed description of the focused classification strategy—used to remove particles containing PSII monomers—has been added to the Methods section, and the figure legend for Supplementary Fig. 2 has been updated accordingly

4. Regarding the composite map construction procedure:

We agree that a more comprehensive description of the composite map construction is needed. The legend for Supplementary Fig. 2o has now been expanded accordingly and reads as follows:

"o, Local resolution map of the SS dimer (composite). The composite map was generated by aligning the two independently refined submaps multiplied with the mask to remove densities outside the masked region (panels m and n) in the lateral plane based on the overall map (panel l) using the 'fit in map' function in UCSF ChimeraX. The alignment was based on their cross-correlated density in the overlapping region. After verification of the precise alignment, the two maps were merged by compositing them into a single map using the 'vop maximum' command in UCSF ChimeraX, which takes the maximum density value at each grid point from the two input maps."

Supplementary Fig. 3 – The micrograph image should be supplied as a size and

resolution that will allow the reader to visualize at least some particles. Class distribution and particle numbers should be shown for part ‘d’ (possibly only the bottom part) and ‘g’. The final number of particles in the dimeric data set should be noted. The tool/procedure to construct the composite map is not described.

We have revised the Supplementary Fig. 3 according to the reviewer’s suggestion and the corresponding text as follows.

1. Regarding the micrograph image:

The original micrograph in Supplementary Fig. 3c has been replaced with a larger, higher-resolution version. Please note that the image, although of high resolution in the original Word file, is subject to clarity loss upon PDF conversion.

2. Regarding class distributions and particle numbers:

As suggested, we have now included the class distributions in Supplementary Figs. 3d (bottom panel), 3h (Supplementary Fig. 3g in the original manuscript) and 3i. The final number of particles used for the dimeric dataset has also been indicated in the Supplementary Fig. 3.

3. Regarding the composite map construction procedure:

A detailed description of the composite map construction has been added to the legend for Supplementary Fig. 3m. The revised legend now reads as follows:

“m, Local resolution map of the TL dimer (composite). The composite map was generated using the same method as described in Supplementary Fig. 2o. Briefly, it was generated by compositing the independently refined submaps from panels k and l based on their alignment to the overall map (panel j).”

Supplementary Fig. 4 – The micrograph image should be supplied as a size and resolution that will allow the reader to visualize at least some particles. It is very

difficult to follow the authors pipeline for this map, I suggest that they will assign distinct colors for each PSII level, that might help. What should be presented very clearly (and is currently unknown) is how many particles contain all three PSII complexes and what is the overlap between sets 1, 2 and 3. Whenever the authors classify their dataset, they should give a measure of the proportion of each class in the total population and specify the number of particles in their final dataset.

We have revised the figure and the corresponding text as follows:

1. Regarding the micrograph image

The original micrograph in Supplementary Fig. 4c has been replaced with a larger, higher-resolution version. Please note that the image, although of high resolution in the original Word file, is subject to clarity loss upon PDF conversion.

2. Regarding the visualization of the pipeline:

We have labeled each PSII unit as $C_2S_2M_2$ in black, $C_2'S_2'M_2'$ in blue and $C_2''S_2''M_2''$ in magenta, and illustrated the workflow for obtaining the TL-TS trimer using dashed lines in three distinct colours to distinguish the processing steps for each PSII level. Together, these facilitate tracking through the classification and reconstruction steps. In the final composite map, these labels are also indicated adjacent to the corresponding densities to help readers follow the processing workflow. We hope this approach also addresses the reviewer's concern about following the pipeline.

3. Regarding the overlap between sets 1, 2 and 3, and particle numbers:

As suggested, we have now added information on how many particles contain all three PSII complexes. The overlap between sets 1, 2 and 3 is illustrated in the updated Supplementary Fig. 4, and the corresponding particle numbers are provided. For each classification step, we have also included the proportion of each class relative to the total population and specified the number of particles used for the final reconstructions.

These details have been added to the figure panels.

Supplementary Fig. 5 and 6 - The micrograph image should be supplied as a size and resolution that will allow the reader to visualize at least some particles. The same comments that were mentioned for S. fig. 4 apply. The authors should provide total number of particles whenever a classification step is carried out, and a clear description of how many particles contained the four PSII complexes in the final composite map, as well as how many complexes are present in each distinct location based on classification or another measure.

We have revised Supplementary Figs. 5 and 6 following the same approach as for Supplementary Fig. 4, and have updated the corresponding text as follows.

1. **Regarding the micrograph image:**

The original micrographs in Supplementary Figs. 5b and 6a have been replaced with a larger, higher-resolution version. Please note that the image, although of high resolution in the original Word file, is subject to clarity loss upon PDF conversion.

2. **Regarding class distributions and particle numbers:**

As suggested, we have now provided the total number of particles at each classification step, as well as the number of particles that contributed to the final composite map for each assembly type. For the tetrameric assemblies shown in Supplementary Figs. 5 and 6, the number of particles containing all four PSII complexes has been indicated. Additionally, the occupancy of each distinct PSII position has been assessed through focused classification, and the corresponding particle numbers are now included in the figure panels.

Supplementary Fig. 7 - The micrograph image should be supplied as a size and resolution that will allow the reader to visualize at least some particles.

The original micrograph in Supplementary Fig. 7a has been replaced with a larger, higher-resolution version. Please note that the image, although of high resolution in the original Word file, is subject to clarity loss upon PDF conversion.

Referee #2 (Remarks to the Author):

This is an exciting, impactful, and technically impressive paper that resolves several long-standing questions in the field of photosynthesis particularly around chloroplast architecture. The use of in situ cryo-EM structural analysis has revealed several aspects of chloroplast organization – including resolving the protein contacts responsible for grana stacking, curvature of the membrane, quinone positions, and in vivo stoichiometry of the supercomplexes. These are important contributions and I recommend that the manuscript be accepted for publication.

The excitation energy calculations have the potential to connect these structural findings to functional aspects of light harvesting, increasing the impact of the work. However, in their current form, these calculations are primarily a control confirming agreement between the supercomplex structures reported here and previously work on in vitro supercomplexes, and so would be more appropriate for the supplementary information. The authors should clarify the insights from energy transfer calculations of the structures through improvements to the methodology and analysis.

We thank the reviewer for this comment, which made us realize that the link between our excitation energy transfer calculations and in vivo functional measurements was not sufficiently clear in the previous version. In the revised version we have clarified that the excitation energy transfer calculations establish a direct connection between the newly resolved $C_2S_2M_2L_4$ supercomplex and functional measurements performed in vivo, showing that for plants grown in low light conditions it is the most dominant form of PSII, likely across higher plant species. Therefore, the excitation energy transfer calculations are central to the manuscript, as they demonstrate the physiological relevance of this newly identified structural organization.

1. Side-by side dimer: the authors conclude energy transfer between PSII supercomplexes in the side-by-side structure is unlikely. However, they used a

simplified FRET model in which the energies of all the chlorophyll a (or chlorophyll b) are equivalent. In such a model, photoenergy does not localize due to lower energy sites, and so the energy transfer pathways can change with the flatter energetic landscape. It is for this reason that more sophisticated methods have been developed and are now typically used in the field (such as the one the authors themselves used for the PSII supercomplexes). Thus, while I agree that energy transfer between side-by-side dimers may be relatively unlikely, the conclusion is not well justified without a more realistic energy landscape.

We thank the reviewer for this comment. We agree that the simplified FRET model used in the original version does not capture the effects of energetic heterogeneity that can influence excitation energy transfer pathways. Following the suggestion of the reviewer we have now calculated the excitation energy transfer dynamics also for the $C_4S_4M_4L_8$ side-by-side dimer using the high-level modified Redfield/Generalized Förster approach, consistently with the methodology applied to the PSII supercomplex. This approach incorporates a realistic energy landscape and accounts for site-energy variations and localization effects. The new results are now included in the revised manuscript (Extended Data Fig. 5). Importantly, they support our original conclusion that excitation energy transfer between PSII supercomplexes in the side-by-side arrangement is relatively unlikely, now on a more rigorous theoretical basis.

2. Trans-stromal dimer: the authors conclude there is no energy transfer between dimers owing to a distance between chlorophylls of ~5 nm. However, the authors also state (173ff): "All types of dimers show substantial variability in angles and distances…" What is the magnitude of this variability? A small change might bring the chlorophylls close enough to enable energy transfer. Can the authors recalculate the rates across the range of observed variability?

We thank the reviewer for this insightful question regarding the relationship between structural heterogeneity and potential energy transfer. As described in our response above and in the "Quantification of structural heterogeneity of PSII dimers" subsection

of the Supplementary Information Methods, we employed a hierarchical refinement approach to measure the relative movement of individual PSII monomers within the dimeric assemblies. Our measurements reveal that each constituent occupies a defined "conformational envelope" characterized by wobbling and shearing. The conformational heterogeneity of individual PSII monomers within dimeric assemblies, quantified as the angular and translational shifts per particle between focused refinement of an individual PSII monomer and consensus refinement of the entire dimer, is now shown in new Supplementary Fig. 12. Nevertheless, the structural variability is mainly described by wobbling and shearing motions under the constraints of the overall parallel membranes. Critically, these membrane constraints prevent the opposing dimers from being brought sufficiently close together across the stromal gap to allow for excitation energy transfer across them. However, at this stage, it is beyond the current cryo-EM data processing algorithms to obtain reliable atomic models (PDB files) that fully capture the continuum of structural heterogeneity observed in the particle population, which would be required for accurate assessment of its impact on energy transfer dynamics. Nevertheless, we believe that our structural characterization of these dimeric assemblies and the observed variability represent a meaningful advance in the field.

3. The authors state: "The weak connection of L-LHCII may facilitate their dynamic association with the C2S2M2 complex, facilitating antenna size regulation, albeit at the cost of a reduced energy transfer efficiency." The authors should better justify this statement – where would the isolated LHCII go? How is it dynamically associated?

We acknowledge that the word "dynamic" in the original version might have been misleading. We mean that upon acclimation to different light conditions, which can take minutes/hours/days, the PSII antenna size in the membrane adapts by changing the amount of L-LHCII. During short-term acclimation, L-LHCII participates in state transitions, moving from PSII to PSII. During long-term acclimation, instead, plants regulate the expression and degradation of Lhcb1-2 subunits (Ballottari et al., 2007),

modulating the overall abundance of L-LHCII in the membrane. We have revised the manuscript both in the results and discussion sections to clarify this point and to better explain how variations in L-LHCII content relate to structural organization as well as to excitation energy transfer and trapping properties.

4. The authors calculate the predicted lifetimes for supercomplexes of different sizes, which is compared to other measurements in Fig 5b. Can the authors further analyze this data to make any conclusions about the likely size of supercomplexes in other species/growth conditions?

Following the suggestion of the reviewer we have now also compared our simulations to the study by Wientjes et al. (2013b), where PSII lifetimes were reported for plants acclimated to different light conditions containing an average of 2.4 and 3.1 LHCII trimers per PSII reaction center. To this end, we simulated decay kinetics assuming a heterogeneous population of PSII supercomplexes ranging from $C_2S_2M_2$ to $C_2S_2M_2L_4$. The resulting ensemble-averaged lifetimes show very good agreement with the experimental observations, further supporting the validity of our modelling approach and supporting the notion that the L-LHCII trimers are the main components that are regulated under long-term light acclimation across higher plants.

Referee #3 (Remarks to the Author):

This manuscript describes an in situ structural analysis of PSII and PSI from chloroplast of O. sativa by cro-EM. It was found that PSII-LHCII exists not only in a C2S2M2L4 state, but also organizes into three distinct higher ordered forms of (1) side-by-side dimer; (2) trans-lumenal (TL) dimer; and (3) trans-stromal (TS) dimer. In addition, both PSI-LHCI and PSI-LHCI-LHCII forms are found in the native membrane. While some of these results are new and worth publication, I have the following concerns that need the authors to consider.

Major points

The authors showed that the membrane stacking is due to TL and TS dimer connections, which are mediated by PsbO and PsbR subunits, respectively, both are PSII core subunits. This shows that the membrane stacking is not related with LHCII proteins. However, there are many previous studies indicating that lacking of LHCII subunits (either by deleting LHCII genes or Chl b synthesizing genes) led to unstacking of the membrane in higher plants. How do the authors explain these previous results?

We thank the reviewer for this comment. As previously reported, even in the absence of LHCII, thylakoid membranes remain partially stacked, although the number and size of grana per unit area are reduced (Andersson et al., 2003; Nicol et al., 2019; Guardini et al., 2022). Unstacking is observed only in plants completely depleted of antenna proteins; however, these lines also exhibit dramatically reduced chlorophyll content, a strong decrease in overall membrane protein density, and a substantial reduction in thylakoid membrane abundance. These observations suggest that the structural alterations may largely reflect changes in membrane protein density and organization, rather than the specific loss of LHCII–LHCII stromal interactions alone.

It is known that electron transfer goes through PSII → cytochrome b6/f → PSI. The authors showed the PSII and PSI structures separately, but do not show their

connectivity nor the structure of cytochrome b6/f complex. If the membrane is intact, as suggested by the authors (because they are using intact chloroplast), it is expected to see the connectivity of PSII and PSI, as well as the cytochrome b6/f complex. Can the authors discuss on this point?

We thank the reviewer fort this insightful comment. We address this below:

1. PSII, PSI and Cyt b_6f may not form stable supercomplexes

It is well established from previous studies that photosynthetic complexes are not uniformly distributed across thylakoid membranes. PSII is predominantly localized in the stacked grana regions, while PSI and ATP synthase are enriched in the unstacked stromal lamellae and grana margins (Rantala et al., *Photochem. Photobiol. Sci.* 2020, 19, 604-619; Andersson et al., *Biochim. Biophys. Acta.* 1980, 593, 427-440). Cyt b_6f is more evenly distributed but is thought to diffuse laterally between these regions (Kirchhoff H, *Biochim. Biophys. Acta.* 2014, 1837, 495-502).

2. Limitation of the in situ method

The thylakoid membrane is crowded with numerous proteins. High-resolution in situ cryo-EM is highly challenging in most cases and usually requires target complexes of sufficient size, stability and abundance to enable accurate alignment and classification. Because PSII, PSI, and cyt b_6f are spatially separated, they are less likely to form stable supercomplexes in high abundance. Moreover, the *O. sativa* cyt b_6f complex, with an estimated molecular weight of approximately 220–230 kDa in its dimeric form, is relatively small with diffusive conformations, and we hope to resolve its high-resolution structure in the future along with methods development.

Page 9, line 179-, and Fig. 4d: It is stated that "Our high-resolution structures reveal that PSII-LHCII supercomplexes assemble into extensive, higher-order structures that form a PSII-LHCII-based stacking network within the thylakoid membranes". However, there are no evidence showing the higher-order structures of the membrane. Fig. 4d is

only a model of the membrane. Please clarify and comment on this.

We thank the referee for allowing us to clarify our terminology and the experimental basis for the reported higher-order PSII-LHCII assemblies.

1. Regarding the higher-order structures of the membrane

Unlike proteins, the membrane around PSII lacks a homogeneous 3D structure, causing its signal to cancel out during averaging in 3D reconstruction. Thus, membrane density is too weak to be seen in the map. Importantly, our direct freezing of chloroplasts on the grid retains native lipids and weakly bound subunits that are lost with detergent use, confirming that PSII is within a membrane environment. Therefore, we used the assembled PSII-LHCII supercomplexes to propose a higher-order membrane-protein structure.

2. Fig. 4e as a proposed schematic model of grana architecture shaped by PSII supercomplexes

Based on the higher-order PSII assemblies and the PsbO/PsbR-mediated stacking network, Fig. 4e (the reviewer's mention of Fig. 4d is assumed to be a typographical error) presents a schematic model illustrating how these interactions might organize stacked grana membranes. The model directly derives from our experimentally determined structures and represents possible assembly modes within grana stacks.

3. Revised statement in the main text

To avoid confusion, we have revised the sentence to read: “Our structures reveal that PSII-LHCII supercomplexes within stacked grana membranes can assemble into higher-order structures, which may form a PSII LHCII based stacking network and thereby contribute to grana stacking.”

We hope these clarifications address the reviewer's concern.

Minor points

A C2S2M2L2 form of PSII-LHCII has previously been observed in green algae; this should be mentioned and cited (Sheng X. et al., Nat Plants. 2019; Suga M. et al., Nat Plants. 2019; Shen L. et al., Proc Natl Acad Sci U S A. 2019).

We thank the reviewer for this valuable comment. We have now added the three suggested references (Sheng et al., 2019; Suga et al., 2019; Shen et al., 2019) at the beginning of the second paragraph of the Introduction, following the sentence "…have resolved the supramolecular organization of PSII and PSI from various plants[2-5], algae[6-11]" These references are now cited as references 9-11.

Lines 49: "…photosynthetic bacteria (10-14)". Refs. 10, 11, 12 and 14 cited here are not related with cryo-EM structures of photosynthetic bacteria, instead, two of them are XFEL structural studies of PSII from cyanobacteria, and two are cryo-EM structural studies of cyanobacteria. There are a number of cryo-EM studies regarding the photosynthetic bacterial photosystems, for example:

Qian P, Siebert CA, Wang P, Canniffe DP, Hunter CN., Cryo-EM structure of the Blastochloris viridis LH1-RC complex at 2.9 A. Nature. 2018 Apr;556(7700):203-208. doi: 10.1038/s41586-018-0014-5.

Dong S, Huang G, Wang C, Wang J, Sui SF, Qin X., Structure of the Acidobacteria homodimeric reaction center bound with cytochrome c. Nat Commun. 2022 Dec 14;13(1):7745. doi: 10.1038/s41467-022-35460-6.

We thank the reviewer for the careful correction and for providing the relevant references. We have revised the text and references accordingly. The sentence now reads:

"Recent cryo-electron microscopy (cryo-EM) and X-ray free electron laser (XFEL) structural studies using isolated purified complexes have resolved the supramolecular organization of PSII and PSI from various plants[2-5], algae[6-11], and photosynthetic

bacteria[12-18]."

The original references 10-14, which include XFEL and cryo-EM studies on cyanobacterial photosystems, have been renumbered as 12-16. In addition, the two cryo-EM studies on photosynthetic bacterial photosystems suggested by the reviewer have been added as references 17 and 18.

Fig. 1, In the C2S2M2L2 structure, the L-trimer seems to have no direct interactions with other trimers. How do they bind specifically? In panel k: It should be "PSI core", not "PSII core". Panels h and l could be moved to Extended Data Figs. to reduce the space for the main text.

We thank the reviewer for this comment. We address each point below.

1. Regarding the binding mode of the L-trimer:

We agree that in our current model of $C_2S_2M_2L_4$, the L-trimer does not form direct protein–protein interactions with the adjacent M- and S-trimers. Notably, the density for the L-trimer region is overall weaker than that for the M- and S-trimers (Supplementary Fig. 1), suggesting that the L-trimer exhibits considerable flexibility and dynamics within the membrane plane. We propose that the interaction may be mediated by lipids, which likely explains why the L-trimer is difficult to retain in conventionally purified samples and could only be partially resolved through our in situ approach. Given the current density quality, we are unable to model the precise binding interface at atomic resolution. As stated in the "Model building, refinement, and validation" subsection in the Supplementary Information Methods, we fitted the distinct triangular density with the trimeric LHCII model from the PSI-LHCI-LHCII complex.

2. Regarding the labeling error in panel k:

We thank the reviewer for pointing out this typographical error. The label in Fig. 1k

has been corrected to “PSI core”.

3. Regarding the relocation of panels h and l:

Fig. 1h and Fig. 1l highlight the map quality of in situ $C_2S_2M_2L_4$ and PSI-LHCI-LHCII, respectively, by showing representative densities for endogenous ligands and amino acid residues. We would prefer to retain these panels in the main text if space permits, as they serve to demonstrate the resolution achieved in our reconstructions. Should space constraints require, we are happy to move them to Extended Data Figs.

Legend to Fig. 2: “…with indicated the subunits of the antenna”. Need to rephrase. Not only the antenna subunits but also the core subunits are labeled in the figure.

We thank the reviewer for the careful reading. The Fig. 2 legend has been revised accordingly and now reads: “…with antenna and core subunits indicated.”

Fig. 3f, i: The interactions between neighboring complexes are shown, but the resolution of the maps are low and not sufficient to identify these interactions. How the authors assigned these interactions?

We thank the reviewer for this constructive critique regarding the interfacial resolution in Fig. 3f and 3i. We agree that at the current local resolution, assigning specific atomic interactions directly from the density map alone is challenging.

To address this, we employed a docking-based approach that minimized the possibility of arbitrary assignment. By fitting high-resolution structural models into the cryo-EM density as rigid bodies, the spatial positioning of the subunits was constrained by the overall map envelope. The well-resolved overall architectures of the trans-lumenal and trans-stromal dimers obtained here, particularly in the core regions, provide strict positional constraints for this rigid-body docking, enabling the establishment of reliable models for subsequent interface analysis.

Upon examining these docked interfaces, we observed a consistent enrichment of basic and acidic residues (Extended Data Fig. 4e,f and new Supplementary Fig. 15). Although the local density does not permit the definitive tracing of every individual side-chain rotamer, the close proximity of these charged clusters strongly suggests the formation of a network of electrostatic interactions and hydrogen bonds.

Accordingly, we have revised the legends of Fig. 3f and 3i to clarify that these modeled interactions are representative of the favorable physicochemical environment at the interface, rather than a uniquely determined atomic coordinate.

From the bottom of page 5 to the beginning of page 6: The assignment of four PQ9 was described here and refers to Fig. 9, but PQ9 was not clearly visible in Fig. 9.

We thank the reviewer for their careful reading. We have re-examined the text and the corresponding figure citations, and we recognize that the description may have caused confusion. We clarify as follows:

The sentence describing the cofactors—including "four plastoquinone (PQ) 9 (PL9) molecules, four on each side, assigned as Q_A, Q_B, Q_C and Q_D"—refers to Fig. 2d, e and Supplementary Table 1, where the binding positions and assembly patterns of these cofactors are illustrated. In the subsequent sentence, "Additionally, we resolved 114 functionally associated native lipids (64 LHG, 26 SQD, 17 DGD, 6 LNL and one 3PH molecule, depicted in Fig. 2f and Supplementary Fig. 9a,b)," Fig. 2f shows the relative spatial positions of the four PQ9 (Q_A, Q_B, Q_C and Q_D) and their surrounding lipids within the supercomplex. Supplementary Fig. 9 further provides detailed views of lipid distribution at protein–protein interfaces.

We have reviewed the figures and confirm that the PQ9 molecules are clearly indicated in the panels cited. We hope this clarifies the intended figure references.

Supplementary Information, page 2, lines 7-8: "In our structure reveals…". Need rephrase.

The sentence in the Supplementary Information has been revised and now reads: “Our structure reveals a weak, horizontally oriented density at the Q_C position, suggesting that Q_C may indeed be present.”

Page 8, line 145: “apposition” → “association” ?

We thank the reviewer for the suggestion. The term has been revised to “association” in the main text. The sentence now reads: “On one side, tight association occurs between CP26/PsbZ of one monomer and CP24/Lhcb1 within M-LHCII (M/Lhcb1) of the adjacent monomer”.

Page 9, lines 165-166: “Extended Data Fig. 5f” → may be “Extended Data Fig. 4f” ? In Extended Data Fig. 4f, a PsbV subunit is labeled, but this is a subunit found in cyanobacterial PSII and not present in higher plants. Please clarify.

We thank the reviewer for the careful reading and for pointing out these issues.

The figure callout has been corrected to Extended Data Fig. 4f.

Regarding the labeling in the central panel of Extended Data Fig. 4f, we confirm that the magenta cartoon representations depict PsbR subunits throughout. Specifically, the pair of PsbR that creates a bridging interaction is labeled with a solid magenta outline, and the second pair that approaches each other without direct contact is labeled with a hollow magenta outline. As the reviewer noted, the label for the bridging pair in the central panel was indeed a typographical error, which has now been corrected.

Page 13, line 259: “…five β-carotenes, five Chl a molecules…”. In Extended Data Fig. 6a, b, 4 β-carotenes and five Chl a molecules were shown, but in the legend, it says “4 β-carotenes, 4 Chl a molecules”. Please clarify.

We thank the reviewer for pointing this out. Upon re-examination, we confirm that the correct numbers are four β-carotenes, five Chl *a* molecules, and one DGD, as shown in

Extended Data Fig. 6a,b. These assignments are consistent with the deposited PDB and map data.

The following corrections have been made in the revised manuscript:

Main text (page 14, line 271): Revised to read: "…four β-carotenes, five Chl *a* molecules…"

Extended Data Fig. 6a, b legend: Revised to reflect these corrected numbers.

We thank the reviewer again for helping us improve the accuracy of the manuscript.

Sincerely,

Jiapeng Zhu
